# Supplementary figures and images for: Interleukin-33 regulates the endoplasmic reticulum stress of human myometrium via an influx of calcium during initiation of labor (part 1 of 2)
Source: eLife. 2022 Aug 23;11:e75072. doi: 10.7554/eLife.75072 (PMC9398448; doi:10.7554/eLife.75072)

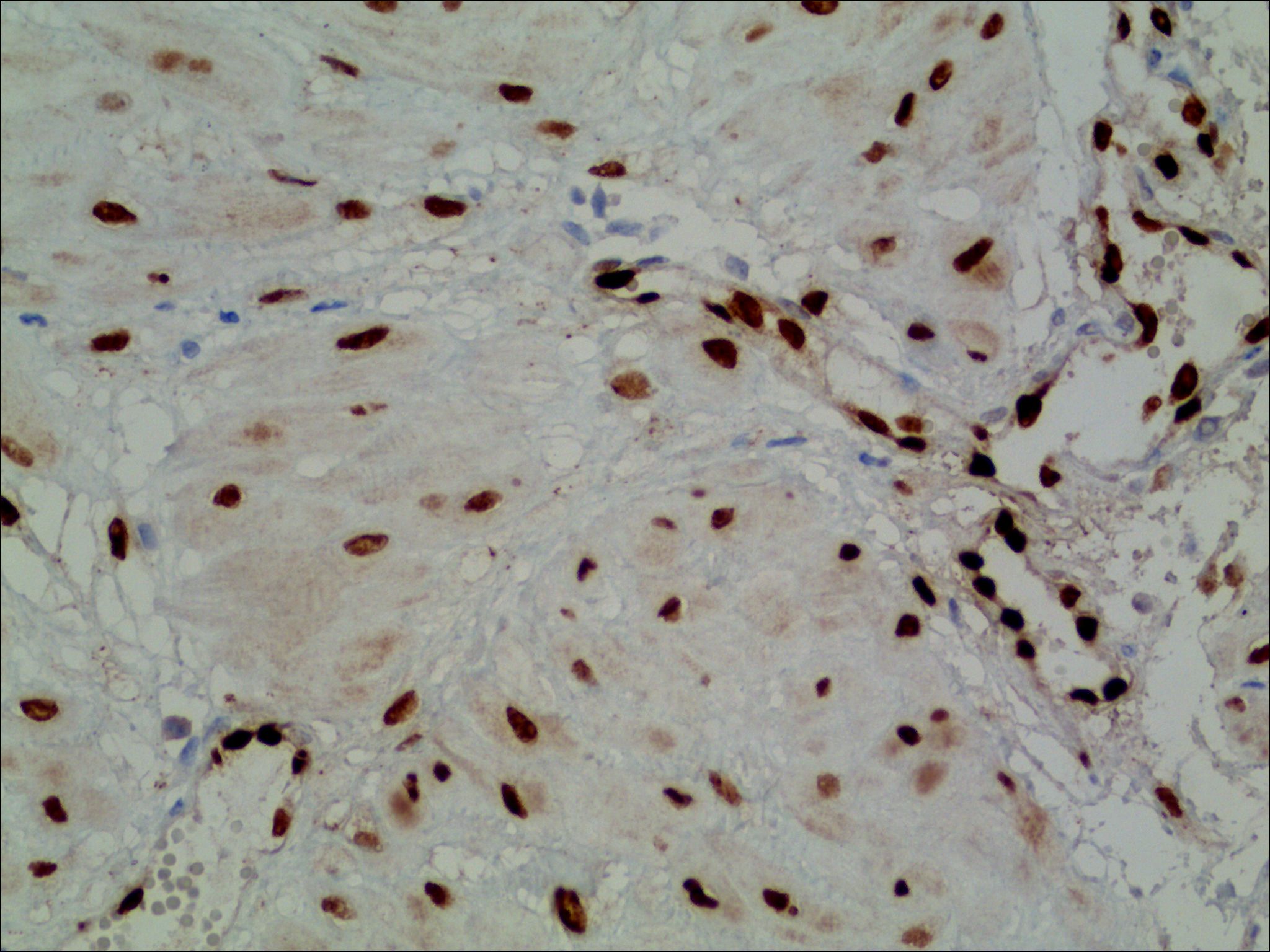

Supplement: Figure 1—source data 1. [file elife-75072-fig1-data1.zip › Figure 1-source data/figure1A/PNL.jpg]

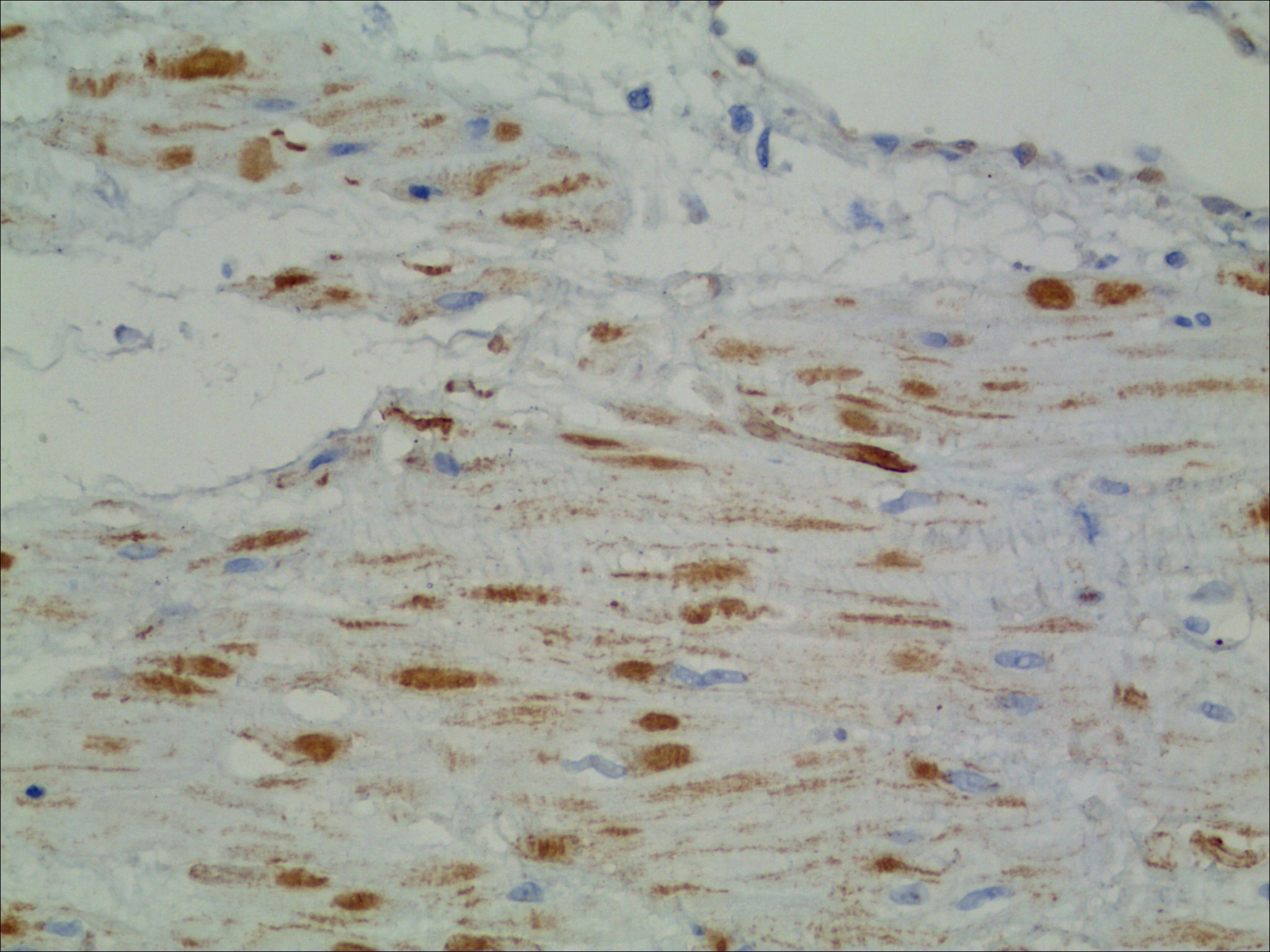

Supplement: Figure 1—source data 1. [file elife-75072-fig1-data1.zip › Figure 1-source data/figure1A/PTL.jpg]

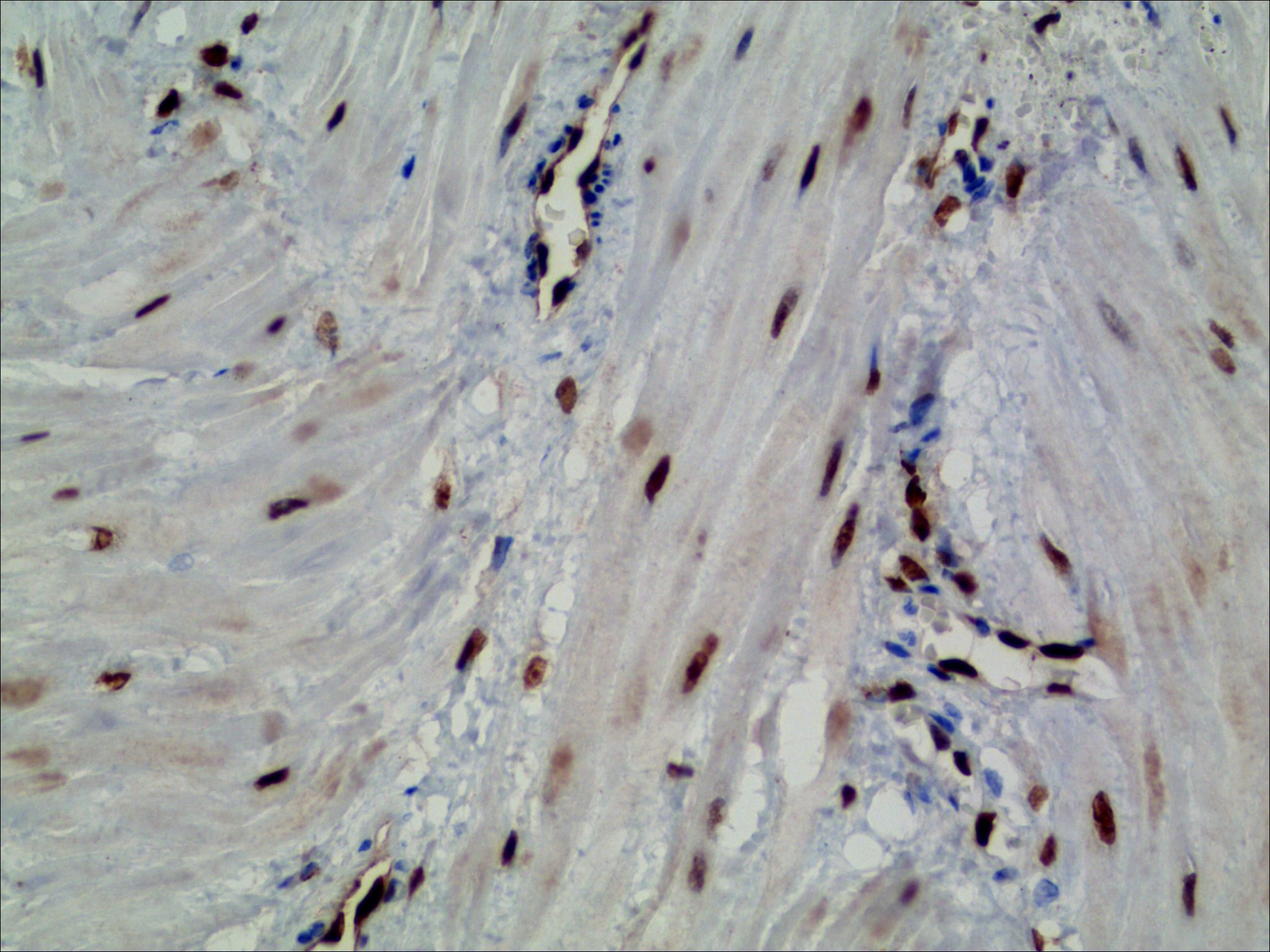

Supplement: Figure 1—source data 1. [file elife-75072-fig1-data1.zip › Figure 1-source data/figure1A/TL.jpg]

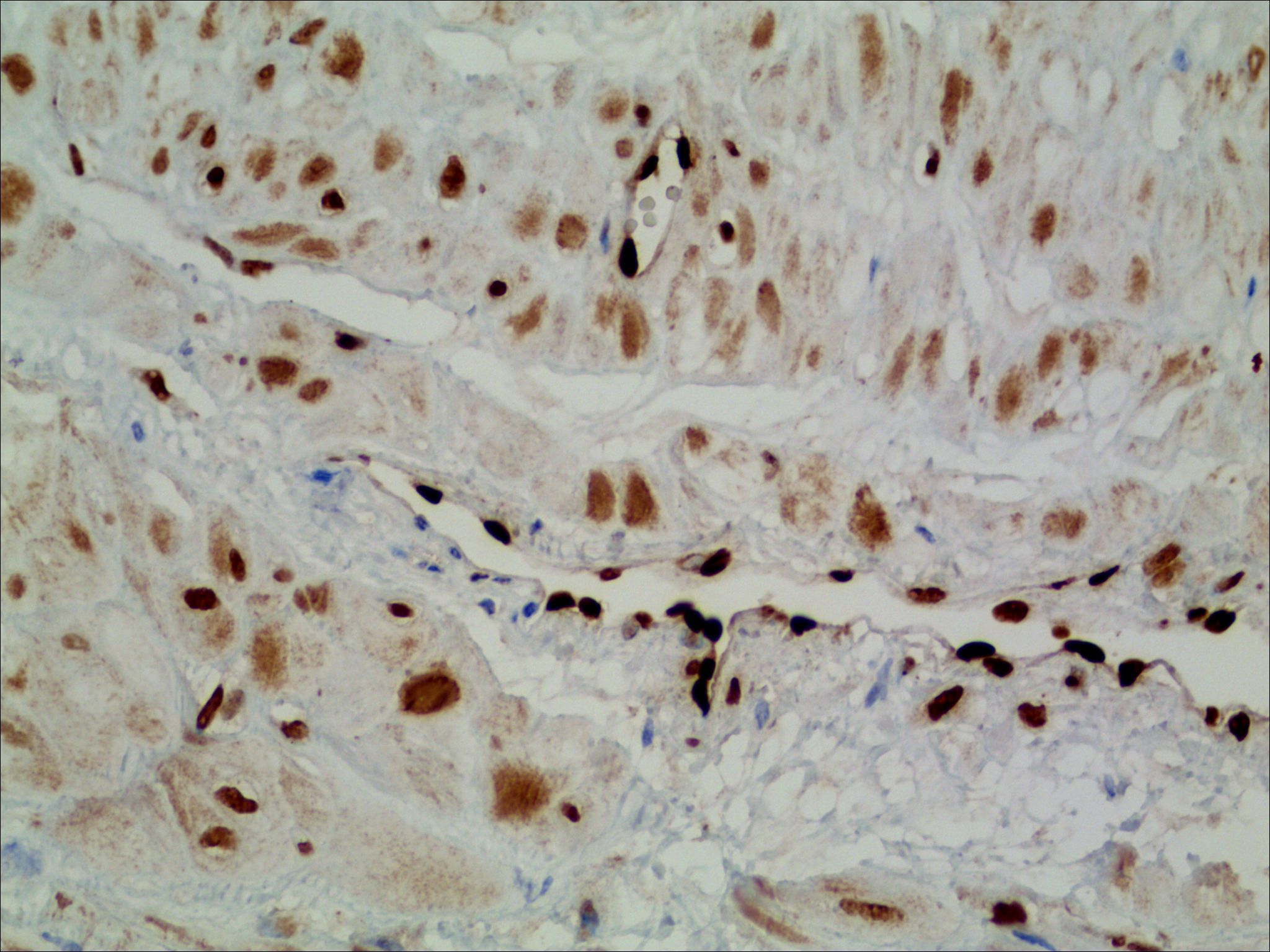

Supplement: Figure 1—source data 1. [file elife-75072-fig1-data1.zip › Figure 1-source data/figure1A/TNL.jpg]

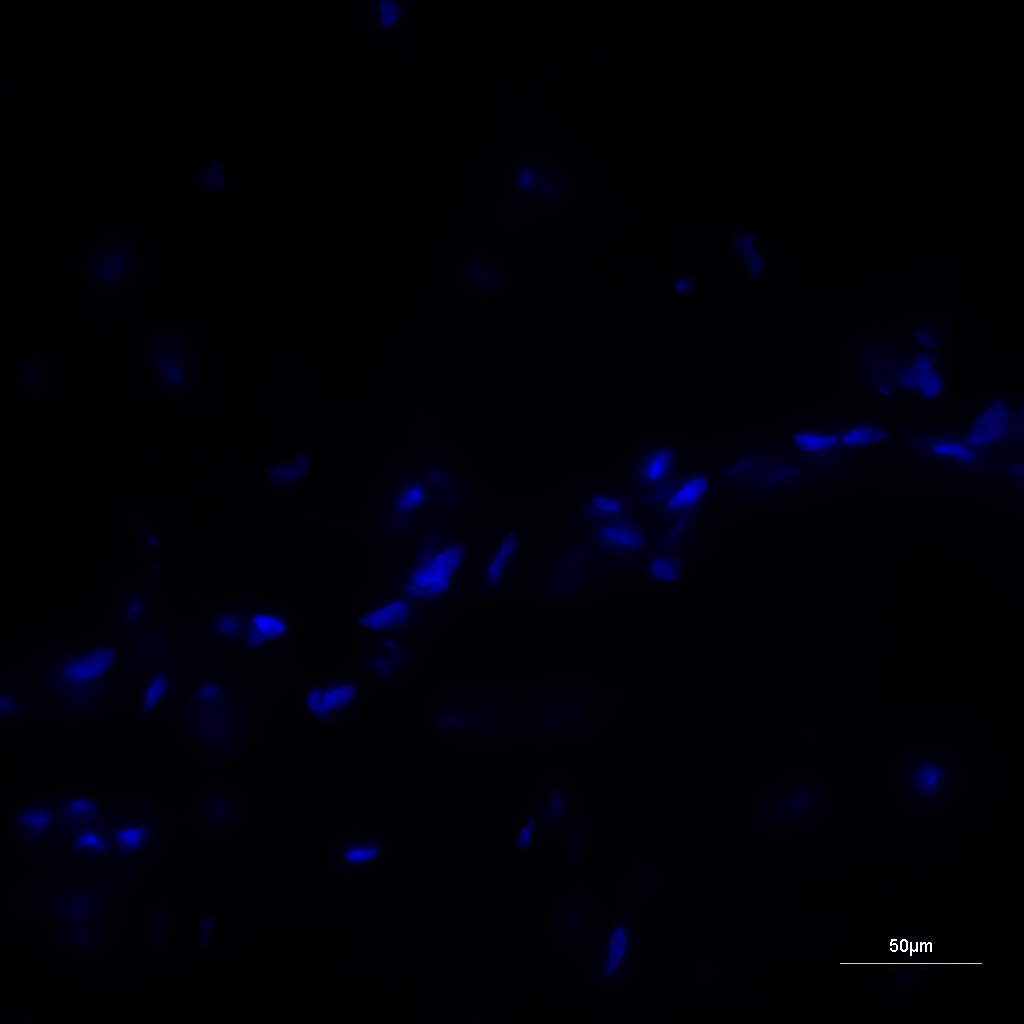

Supplement: Figure 1—source data 1. [file elife-75072-fig1-data1.zip › Figure 1-source data/figure1B/PNL-DAPI.tif]

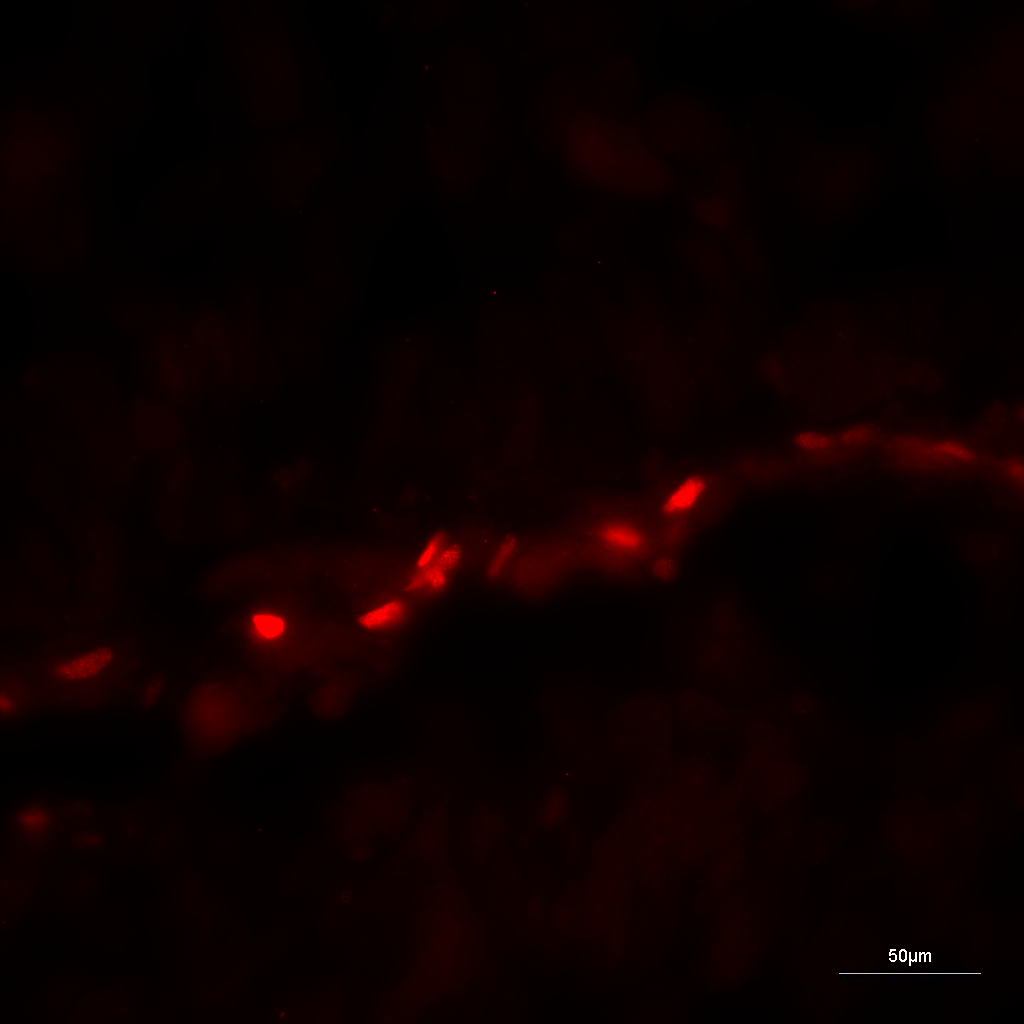

Supplement: Figure 1—source data 1. [file elife-75072-fig1-data1.zip › Figure 1-source data/figure1B/PNL-IL-33.tif]

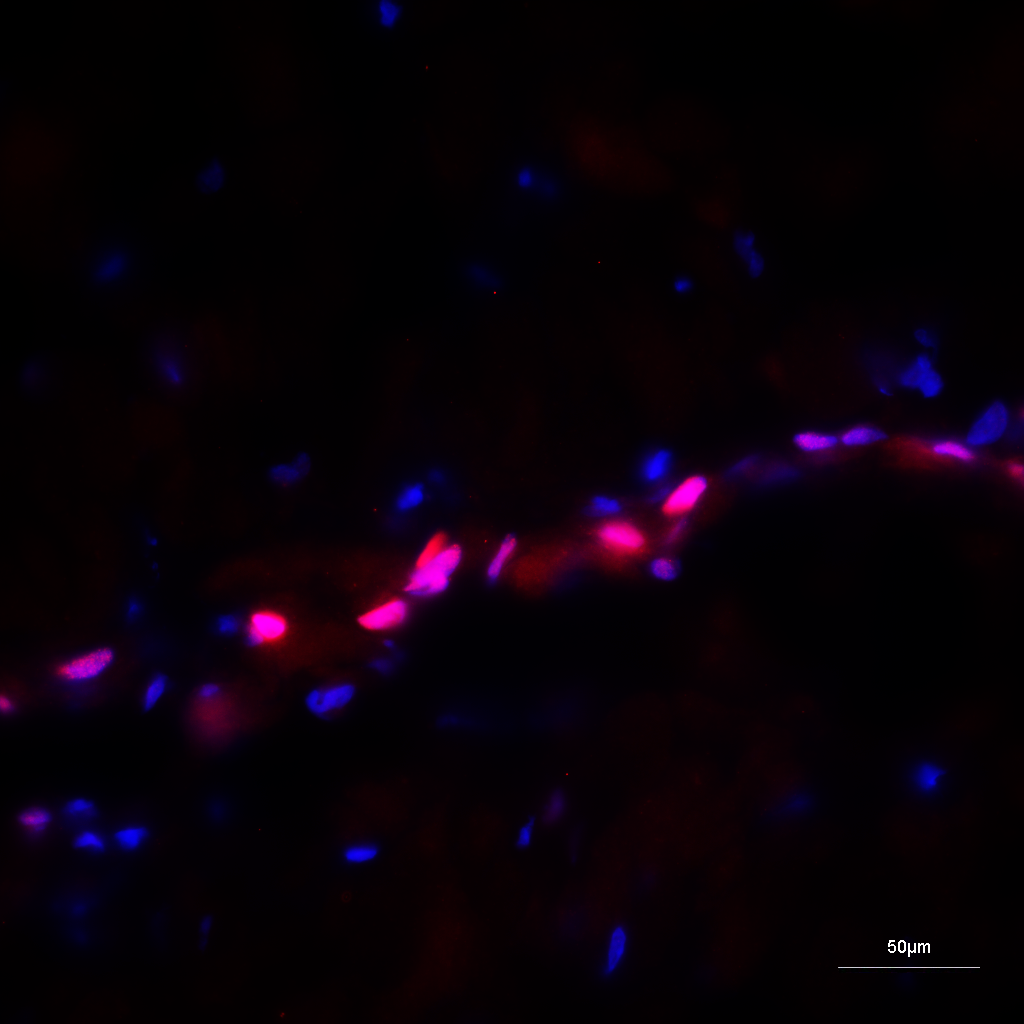

Supplement: Figure 1—source data 1. [file elife-75072-fig1-data1.zip › Figure 1-source data/figure1B/PNL-Merged.tif]

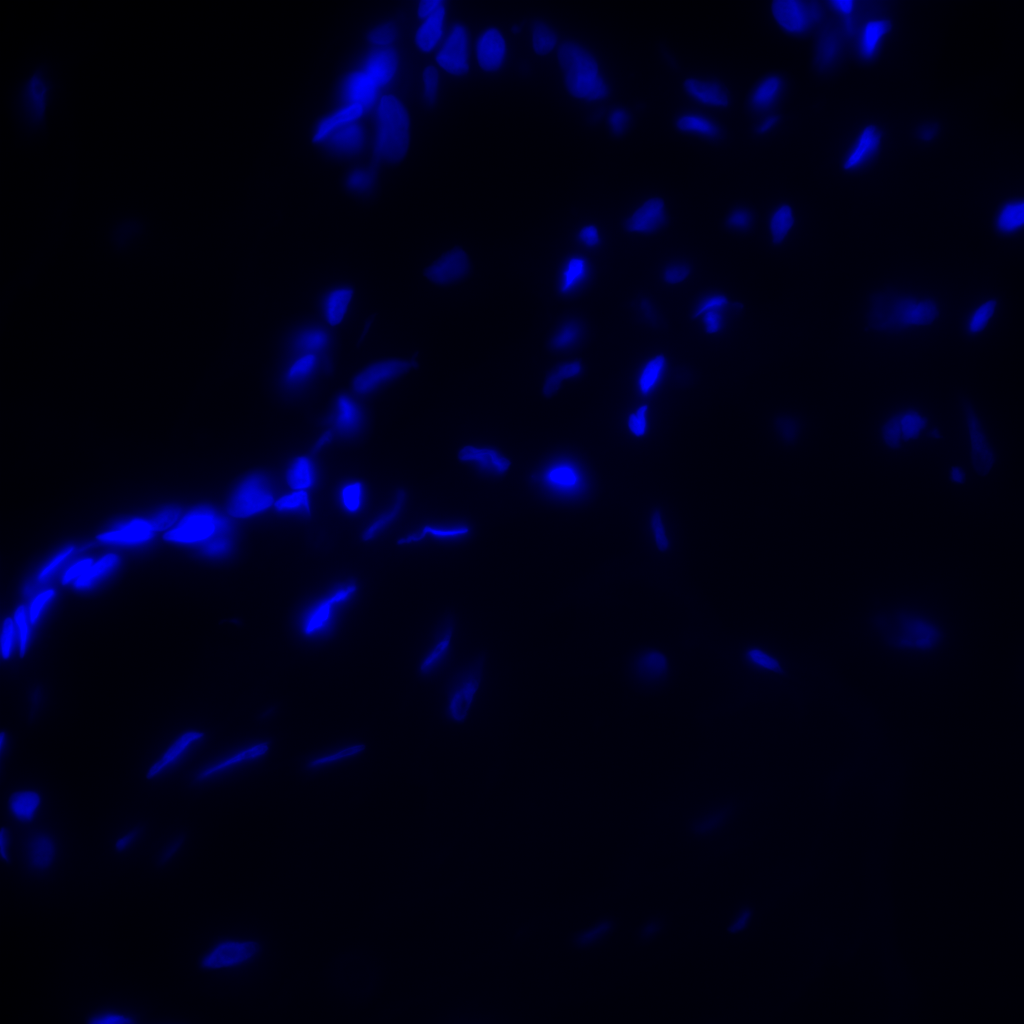

Supplement: Figure 1—source data 1. [file elife-75072-fig1-data1.zip › Figure 1-source data/figure1B/PTL-DAPI.tif]

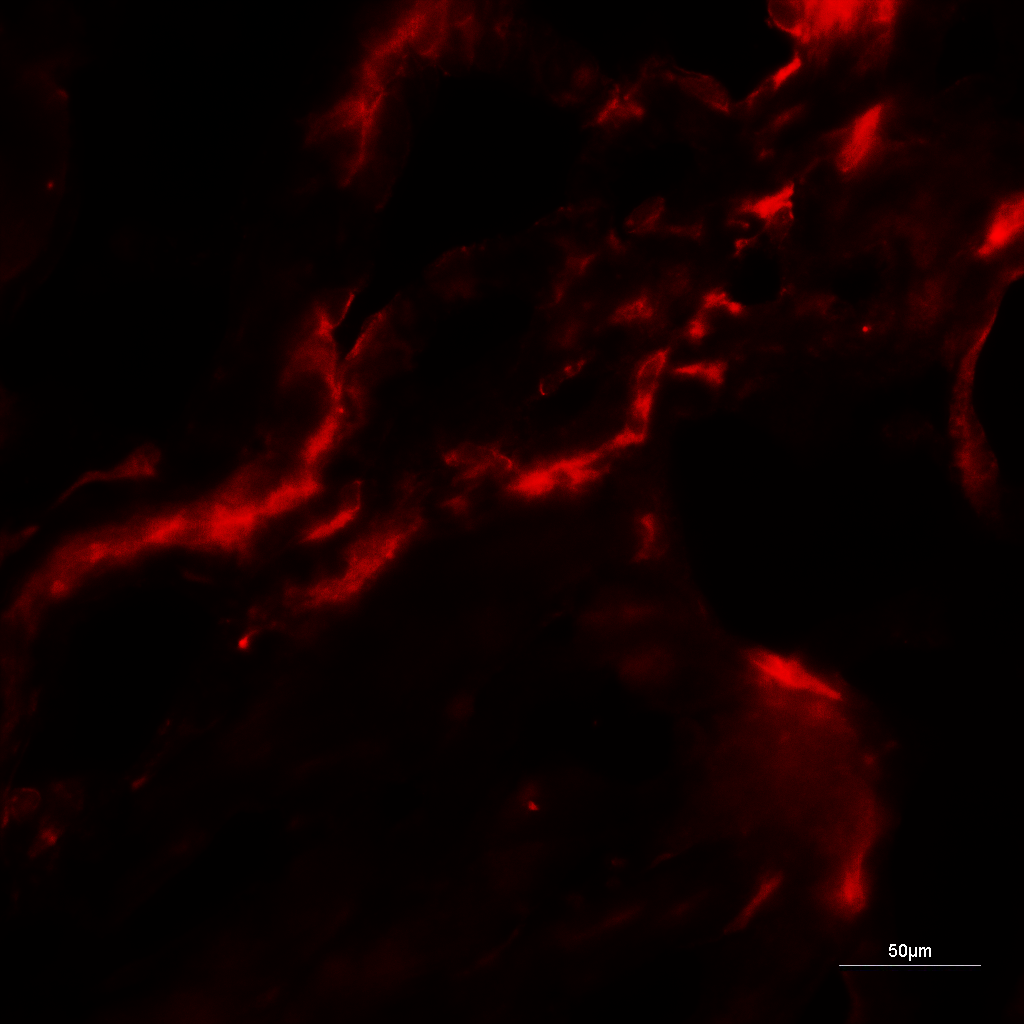

Supplement: Figure 1—source data 1. [file elife-75072-fig1-data1.zip › Figure 1-source data/figure1B/PTL-IL-33.tif]

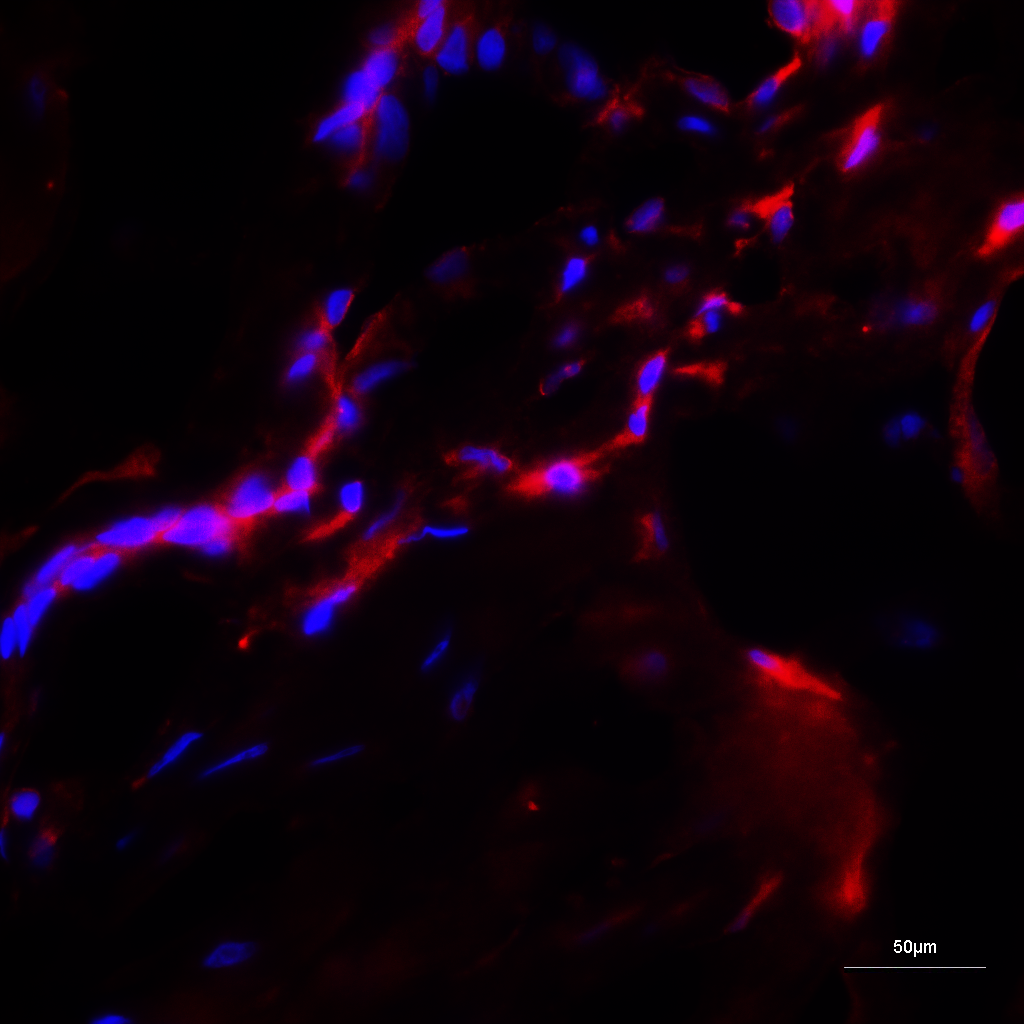

Supplement: Figure 1—source data 1. [file elife-75072-fig1-data1.zip › Figure 1-source data/figure1B/PTL-Merged.tif]

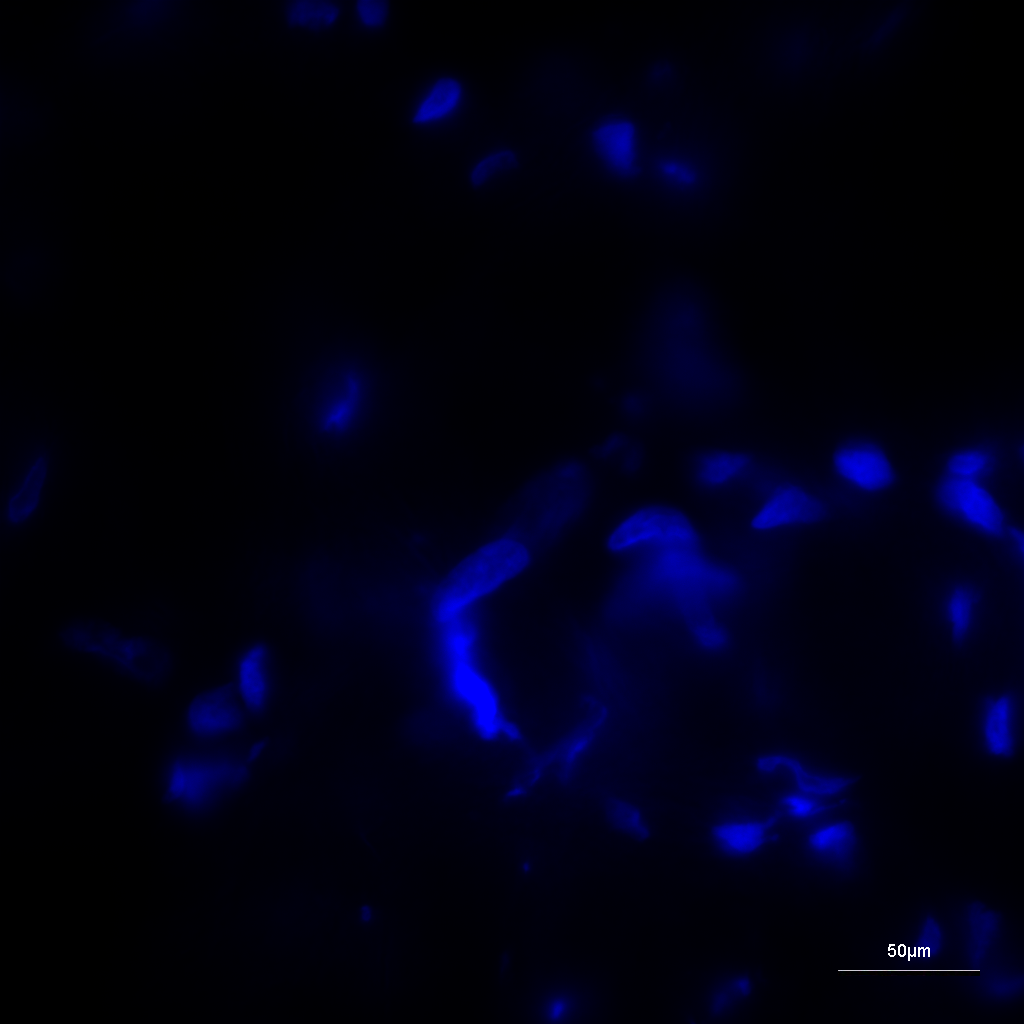

Supplement: Figure 1—source data 1. [file elife-75072-fig1-data1.zip › Figure 1-source data/figure1B/TL-DAPI.tif]

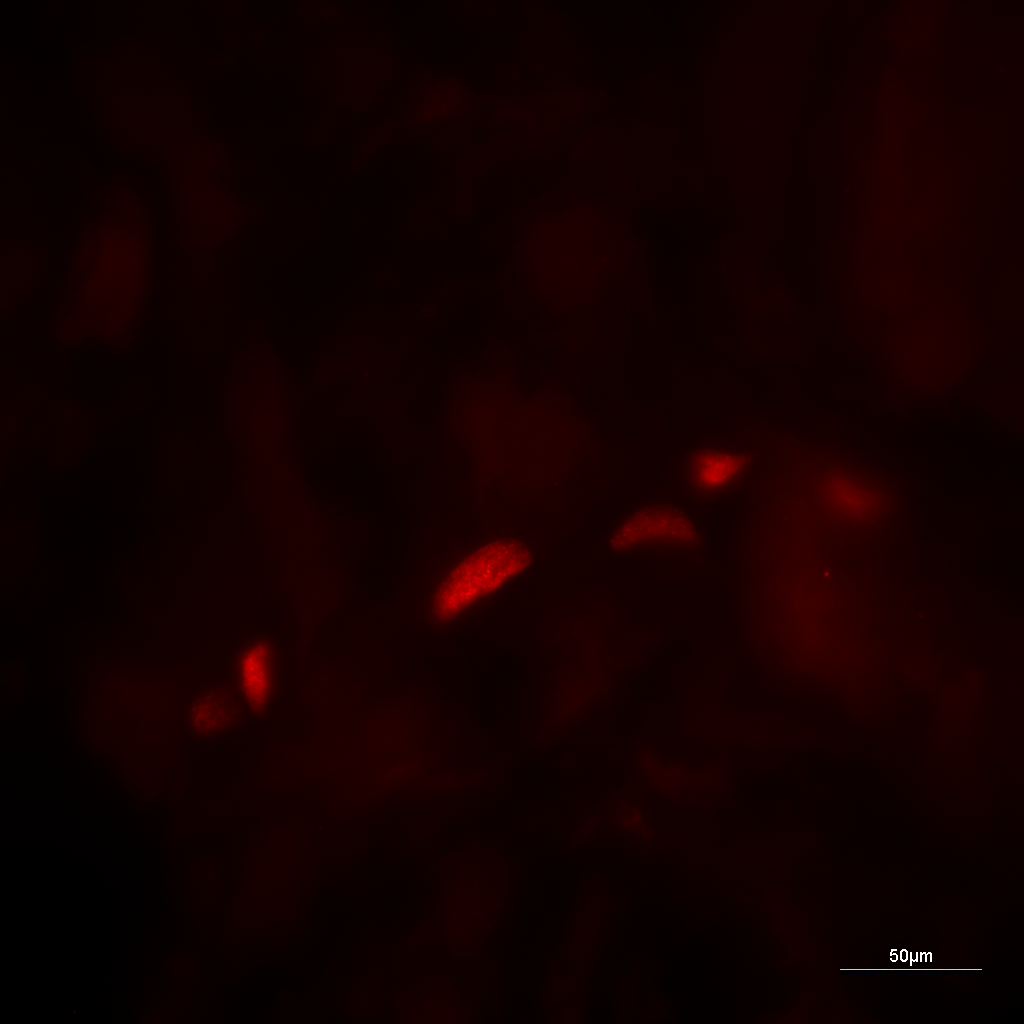

Supplement: Figure 1—source data 1. [file elife-75072-fig1-data1.zip › Figure 1-source data/figure1B/TL-IL-33.tif]

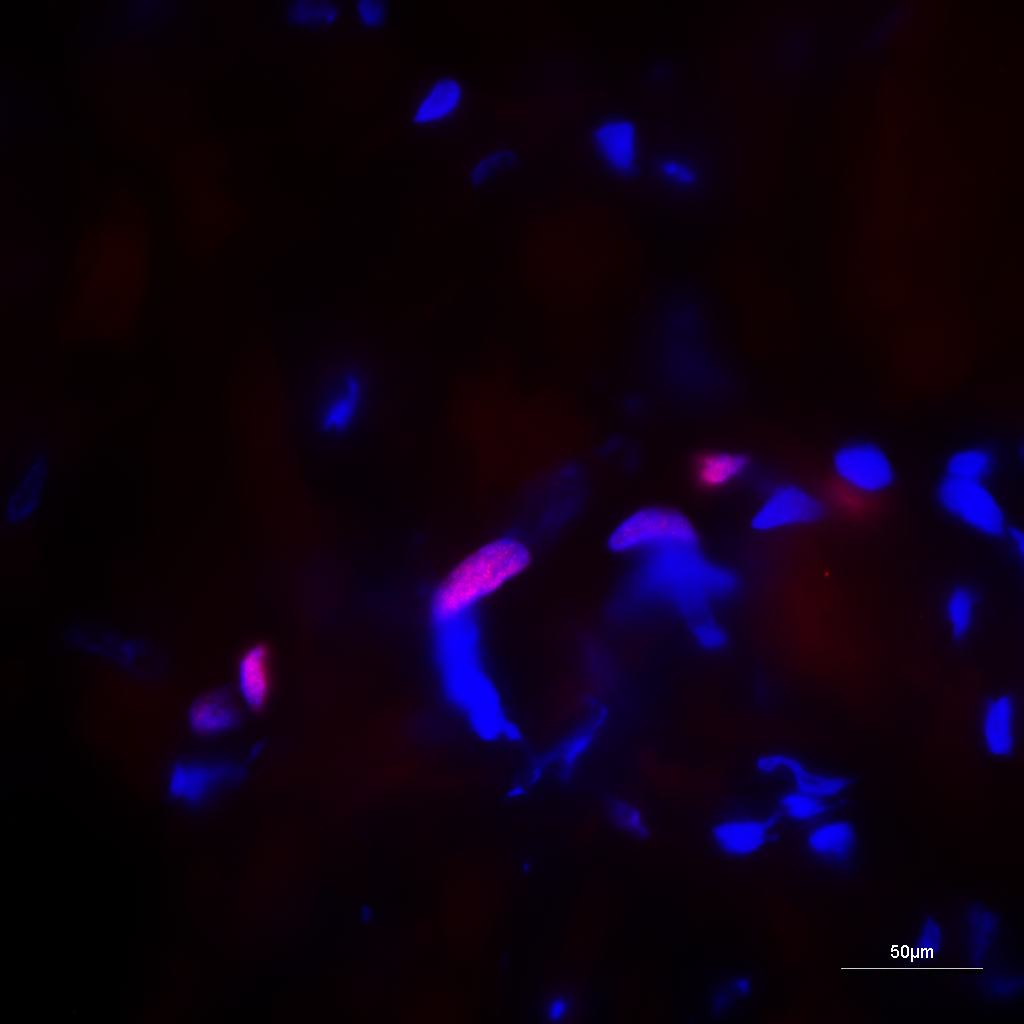

Supplement: Figure 1—source data 1. [file elife-75072-fig1-data1.zip › Figure 1-source data/figure1B/TL-Merged.tif]

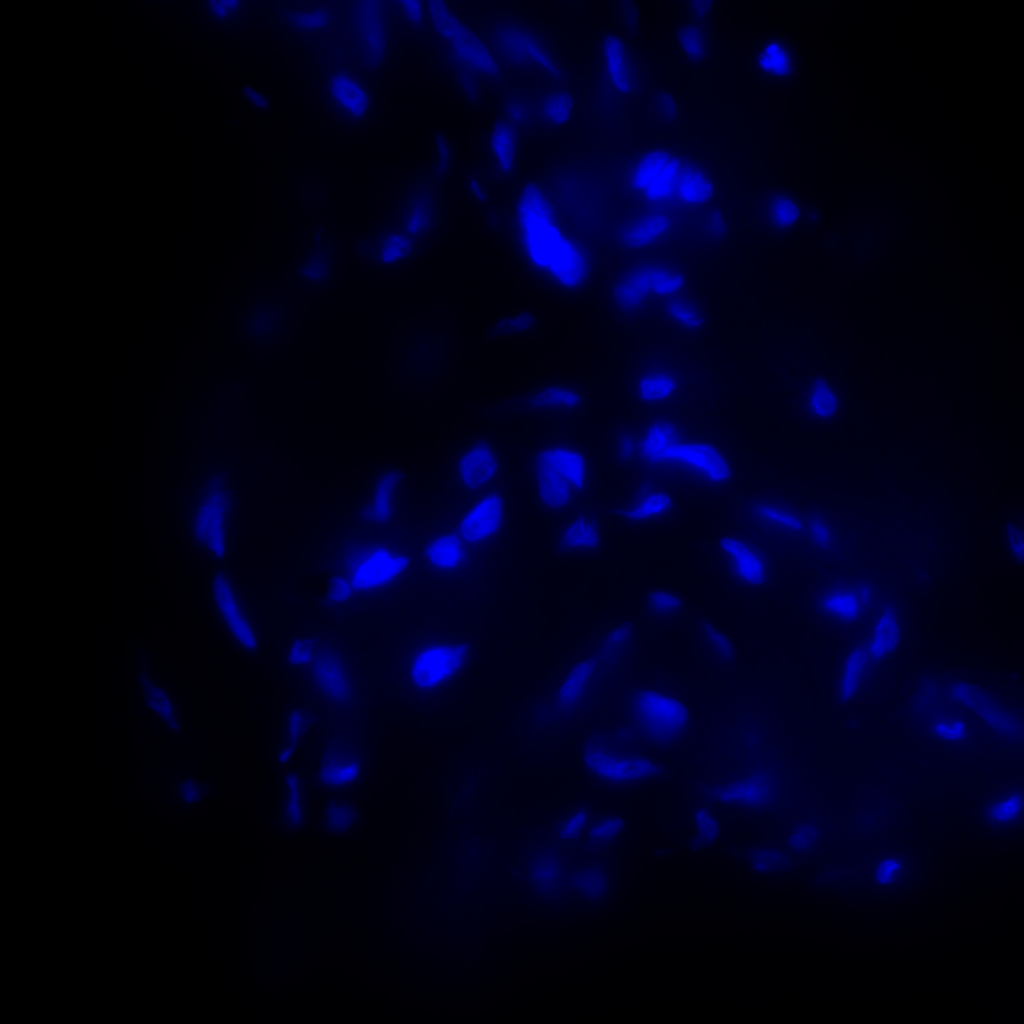

Supplement: Figure 1—source data 1. [file elife-75072-fig1-data1.zip › Figure 1-source data/figure1B/TNL-DAPI.tif]

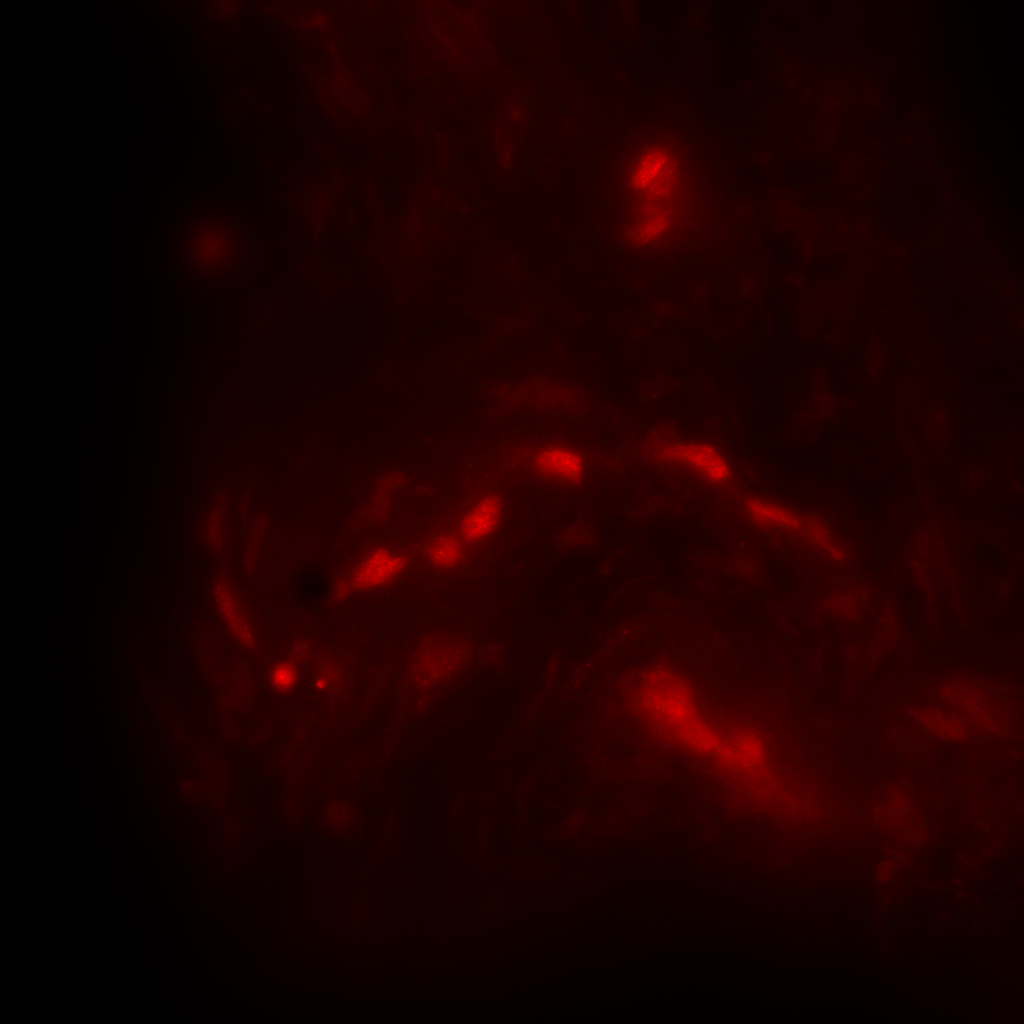

Supplement: Figure 1—source data 1. [file elife-75072-fig1-data1.zip › Figure 1-source data/figure1B/TNL-IL-33.tif]

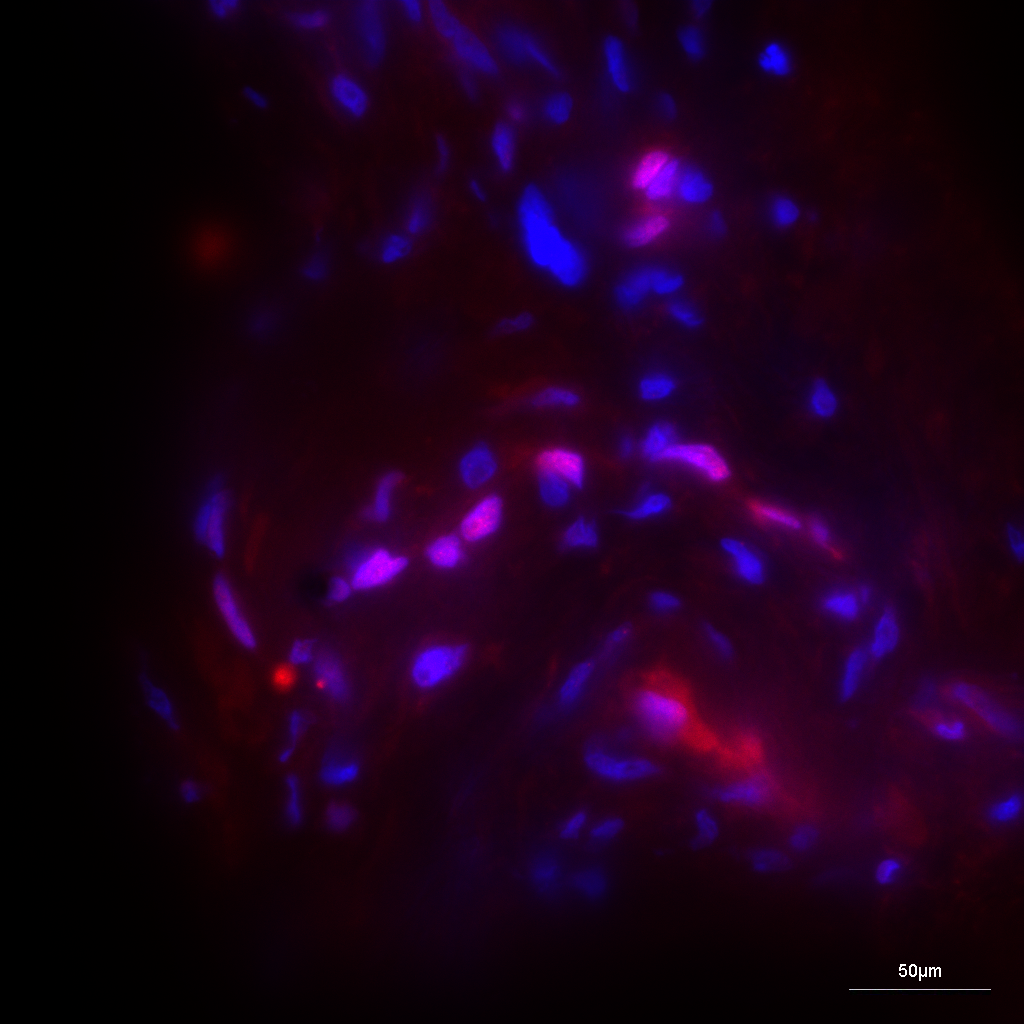

Supplement: Figure 1—source data 1. [file elife-75072-fig1-data1.zip › Figure 1-source data/figure1B/TNL-Merged.tif]

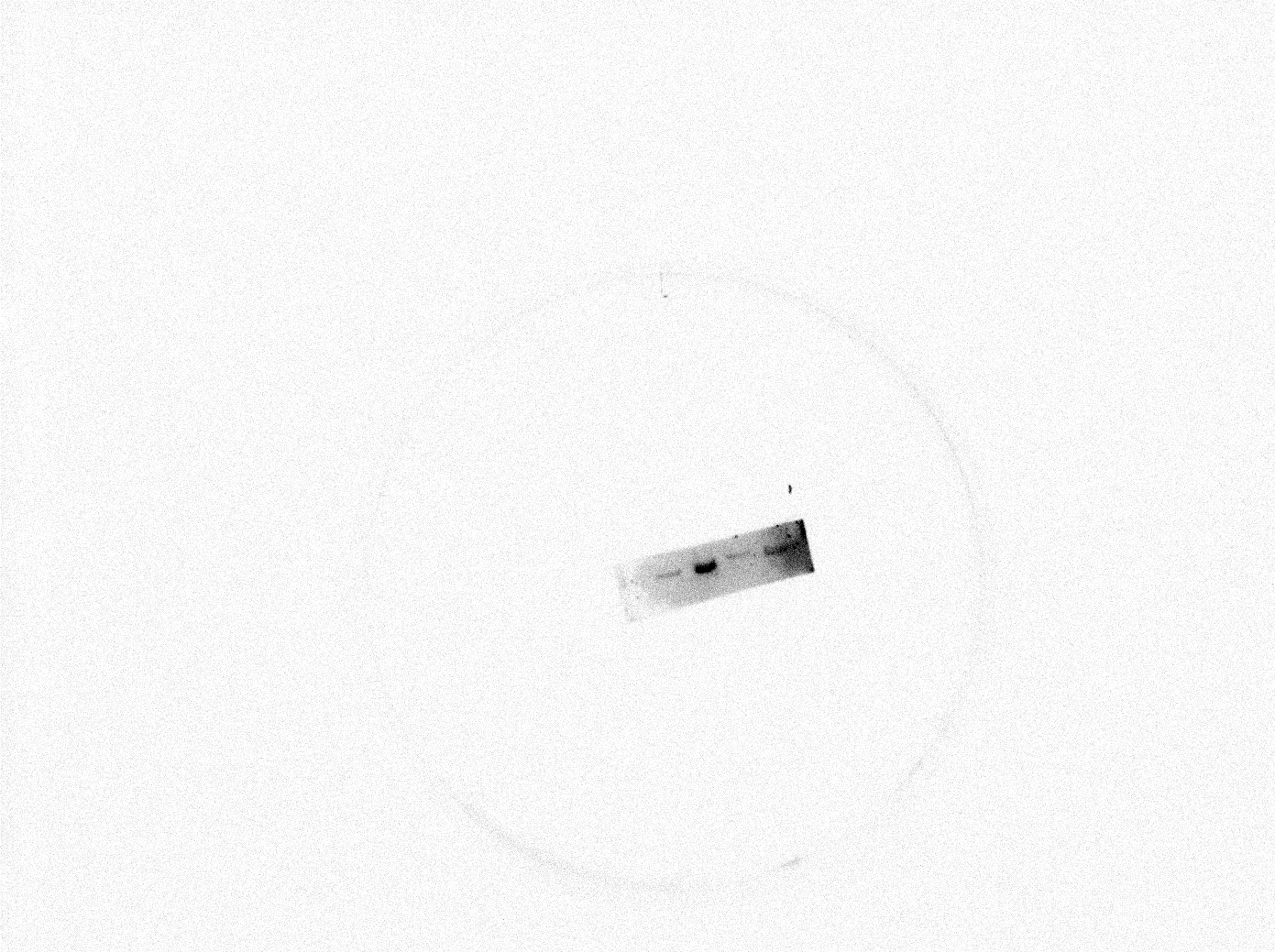

Supplement: Figure 1—source data 1. [file elife-75072-fig1-data1.zip › Figure 1-source data/figure1C/cytoplasmic IL-33.tif]

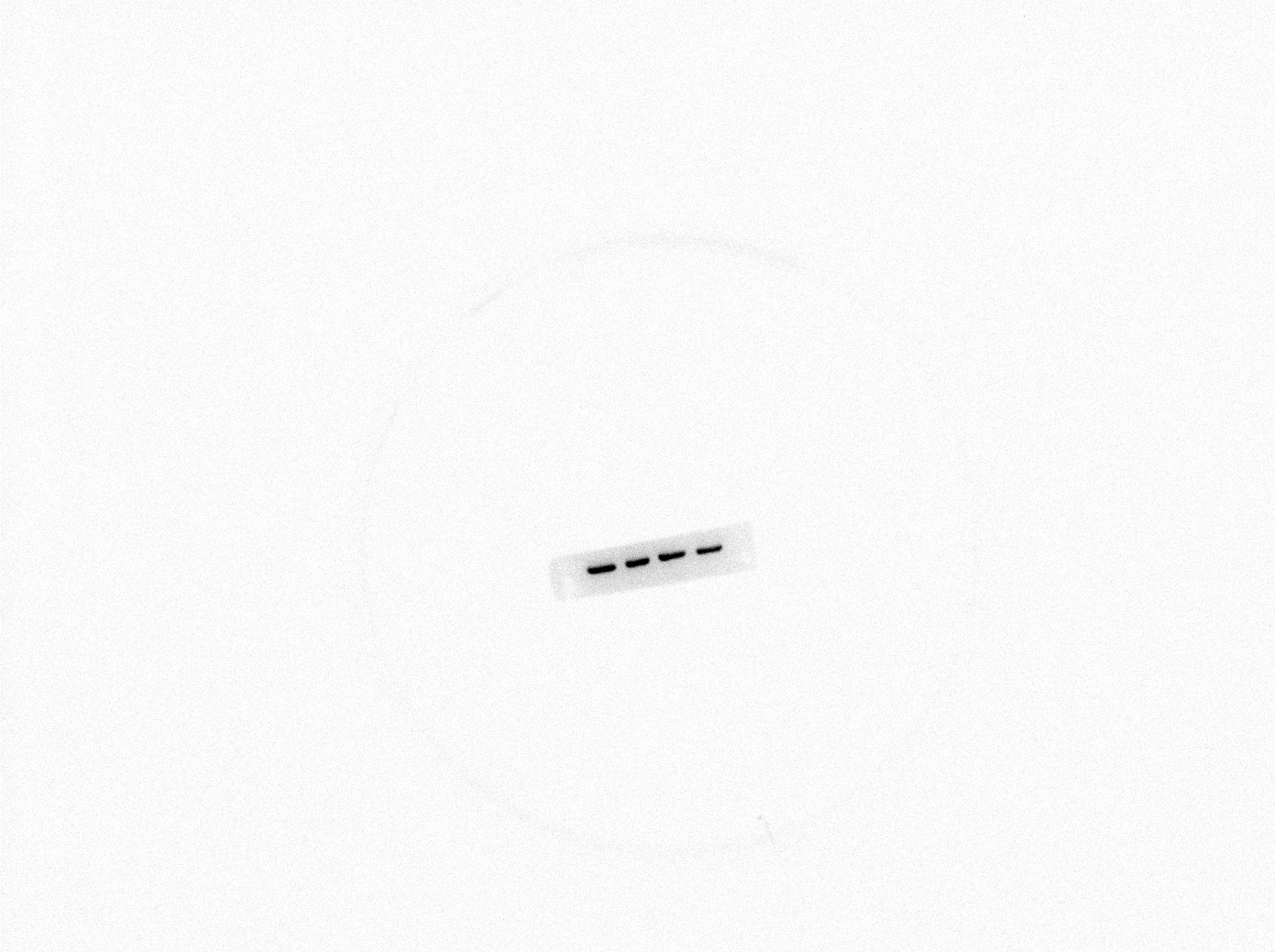

Supplement: Figure 1—source data 1. [file elife-75072-fig1-data1.zip › Figure 1-source data/figure1C/cytoplasmic β-actin.tif]

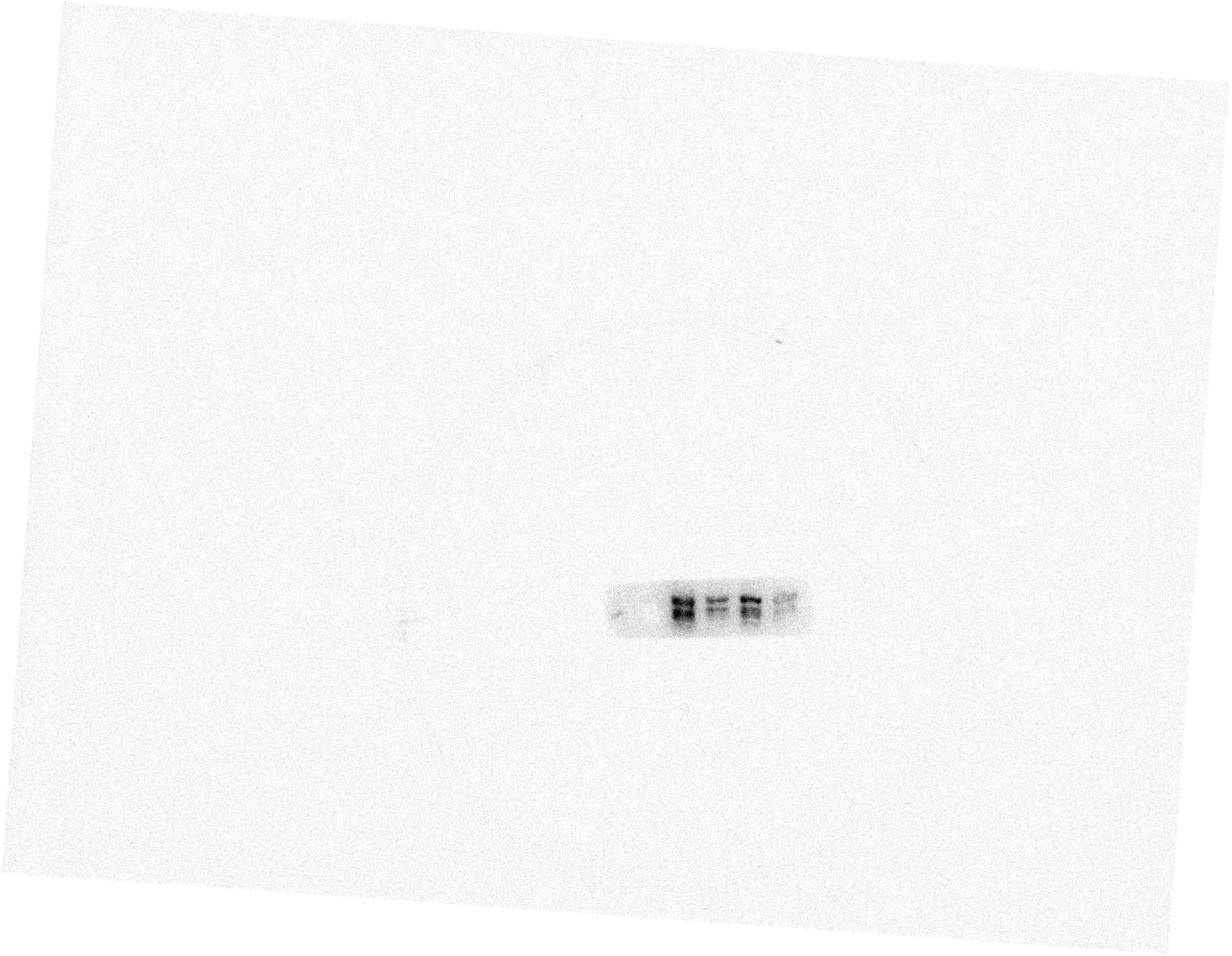

Supplement: Figure 1—source data 1. [file elife-75072-fig1-data1.zip › Figure 1-source data/figure1C/nuclear IL-33.png]

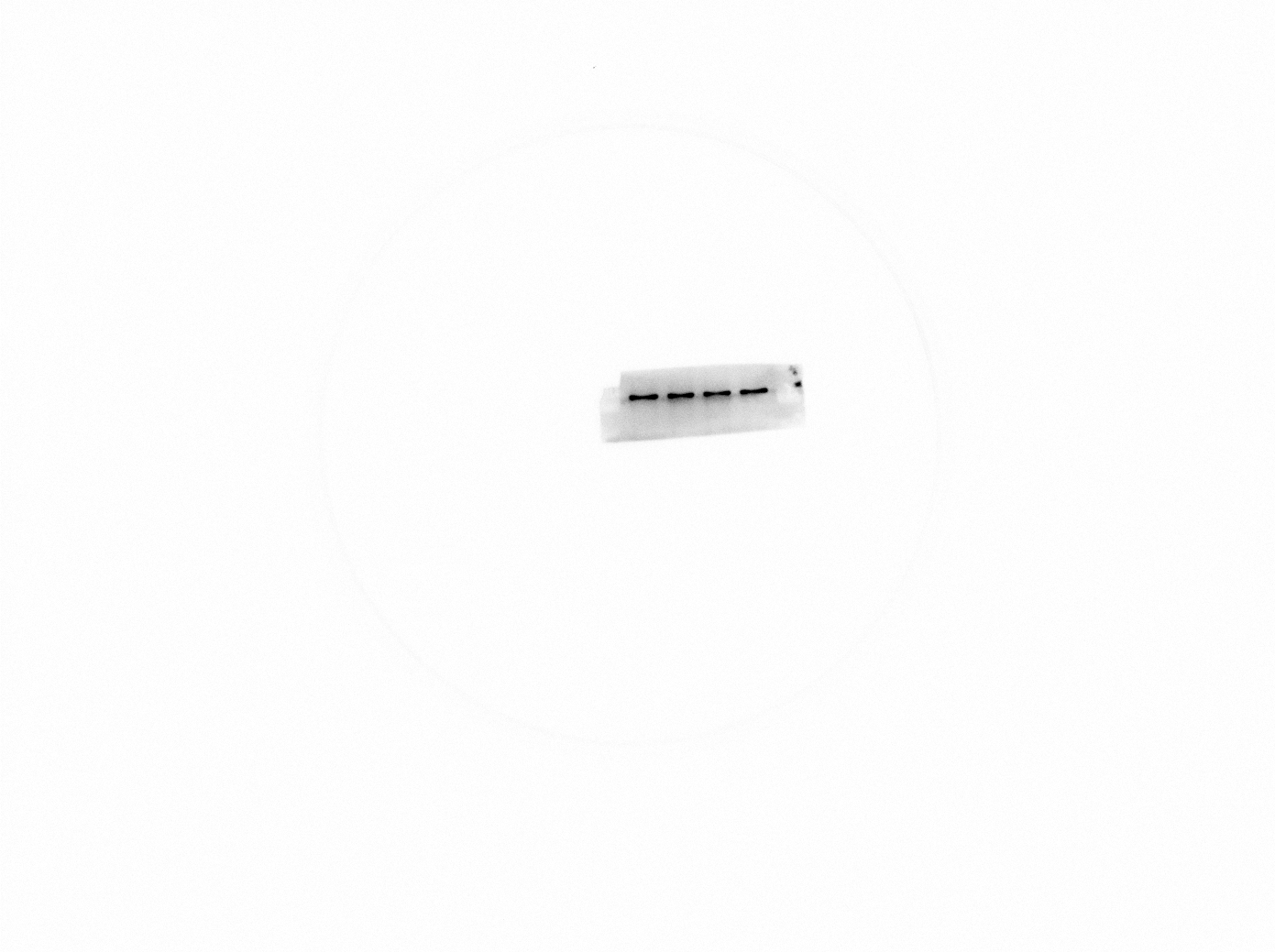

Supplement: Figure 1—source data 1. [file elife-75072-fig1-data1.zip › Figure 1-source data/figure1C/nuclear LaminB1.tif]

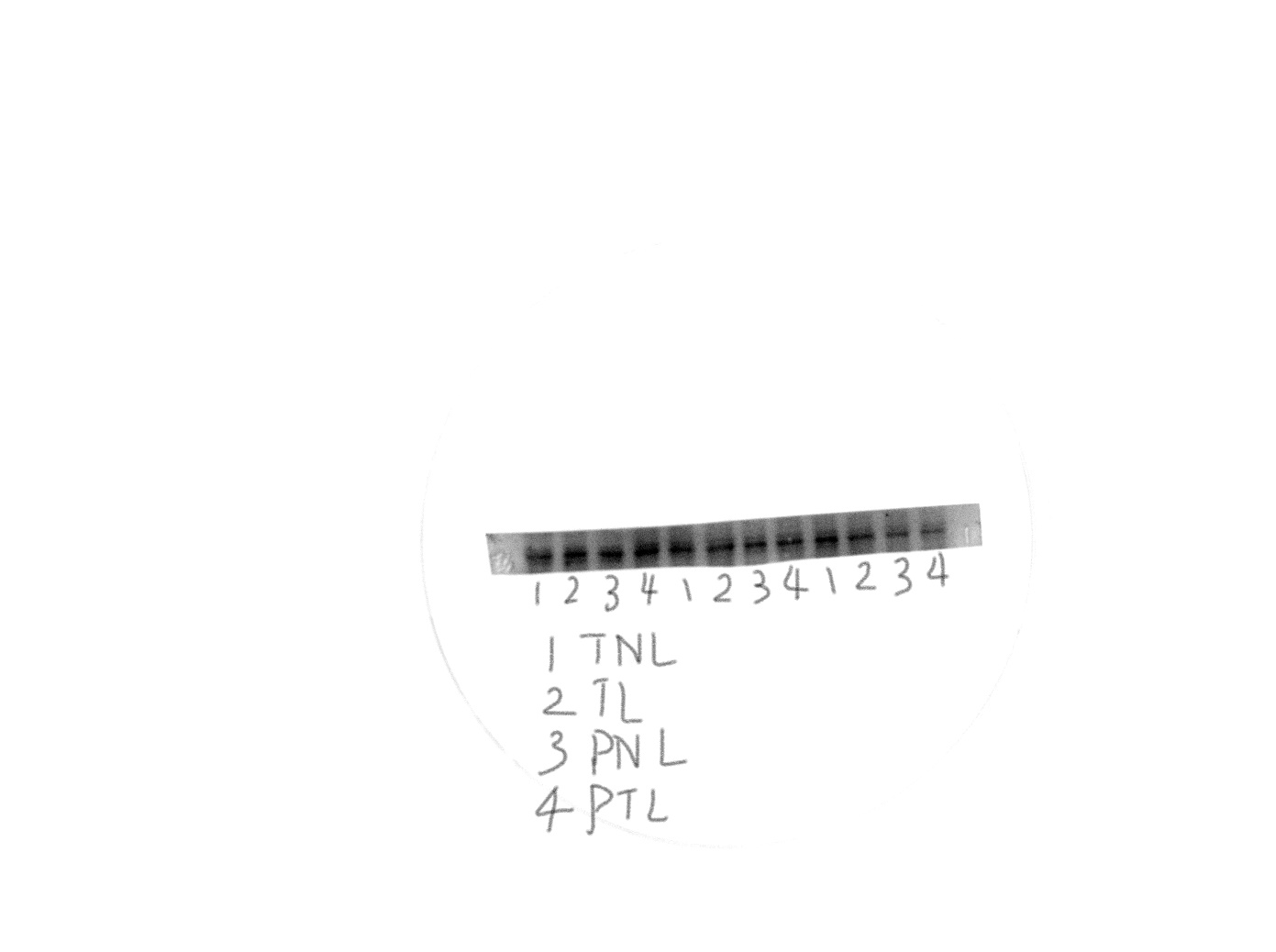

Supplement: Figure 1—source data 1. [file elife-75072-fig1-data1.zip › Figure 1-source data/figure1C/total IL-33.jpg]

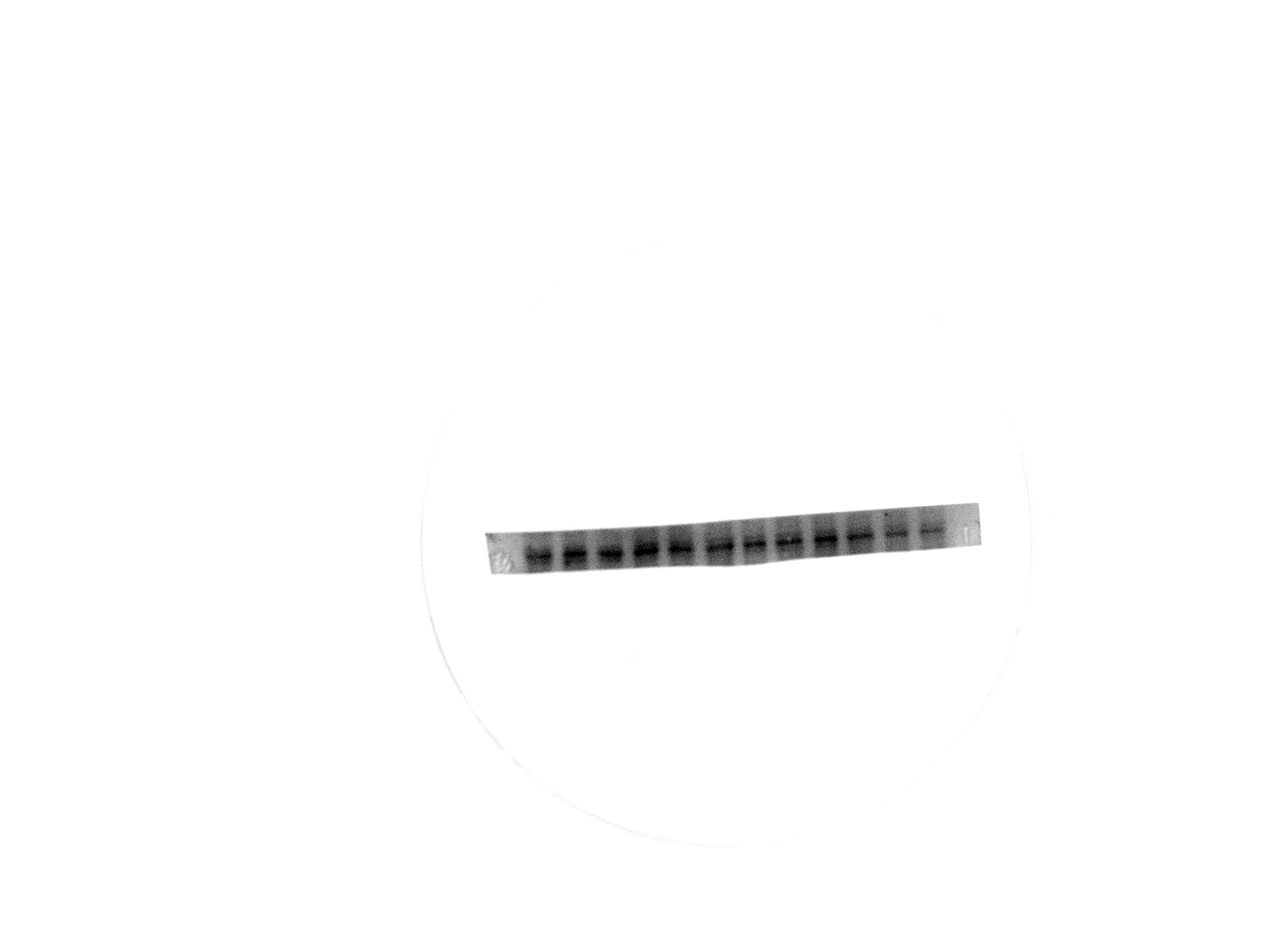

Supplement: Figure 1—source data 1. [file elife-75072-fig1-data1.zip › Figure 1-source data/figure1C/total IL-33.tif]

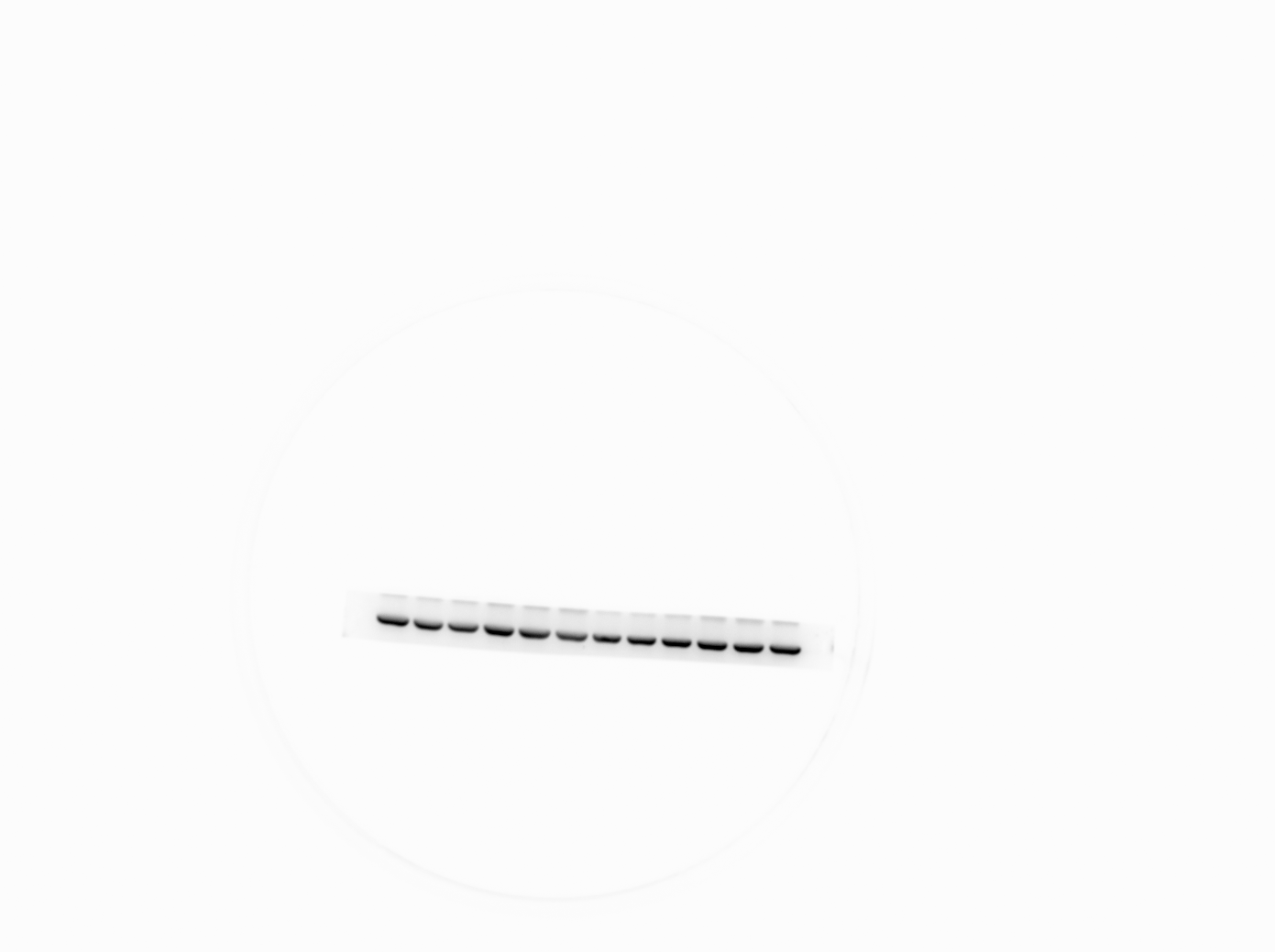

Supplement: Figure 1—source data 1. [file elife-75072-fig1-data1.zip › Figure 1-source data/figure1C/total β-actin.tif]

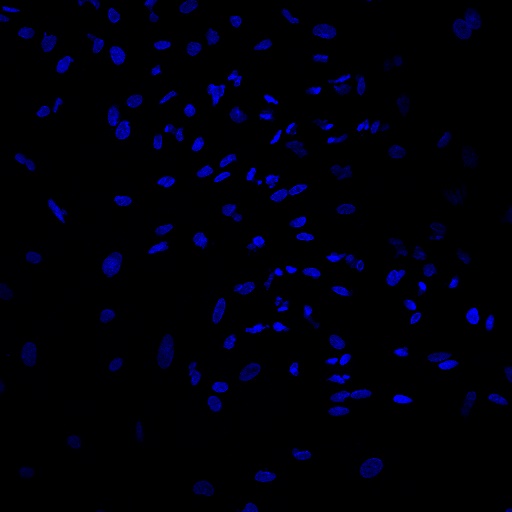

Supplement: Figure 2—source data 1. [file elife-75072-fig2-data1.zip › Figure 2-source data/figure2A/0h(Control)/DAPI.jpg]

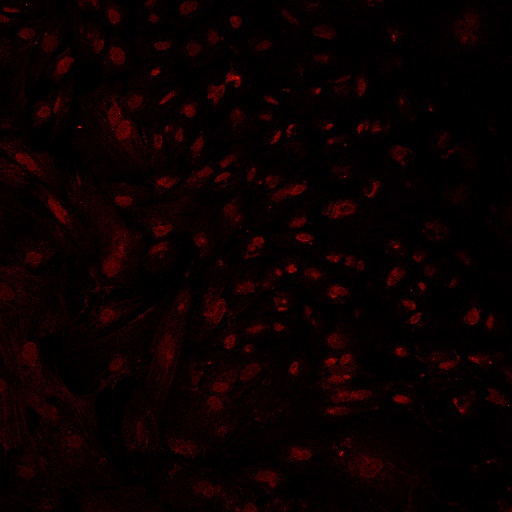

Supplement: Figure 2—source data 1. [file elife-75072-fig2-data1.zip › Figure 2-source data/figure2A/0h(Control)/IL-33.jpg]

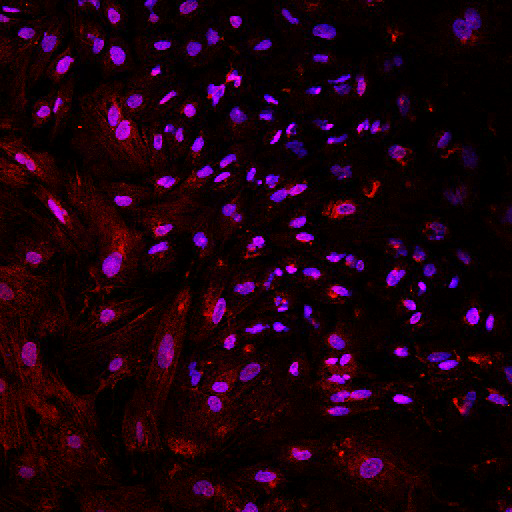

Supplement: Figure 2—source data 1. [file elife-75072-fig2-data1.zip › Figure 2-source data/figure2A/0h(Control)/merged.png]

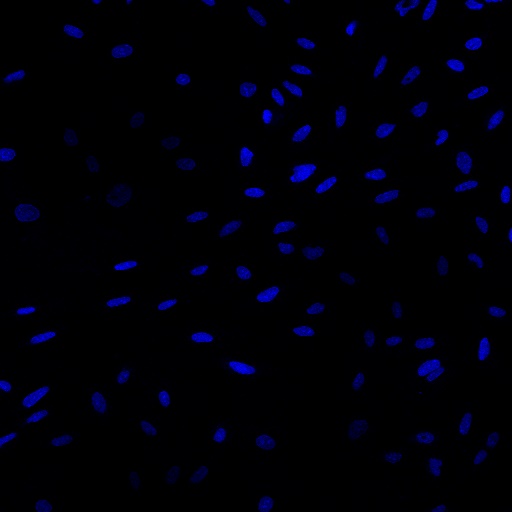

Supplement: Figure 2—source data 1. [file elife-75072-fig2-data1.zip › Figure 2-source data/figure2A/12h/DAPI.jpg]

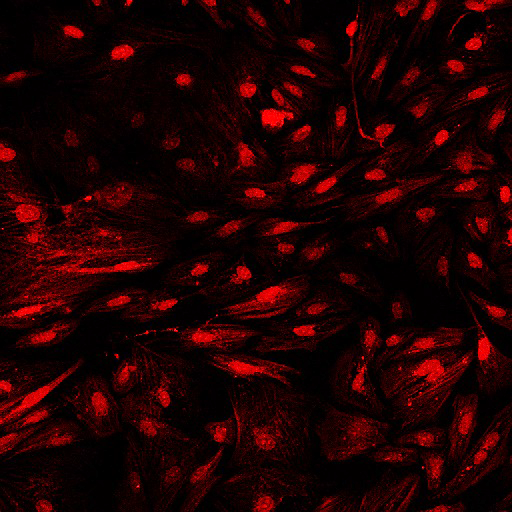

Supplement: Figure 2—source data 1. [file elife-75072-fig2-data1.zip › Figure 2-source data/figure2A/12h/IL-33.png]

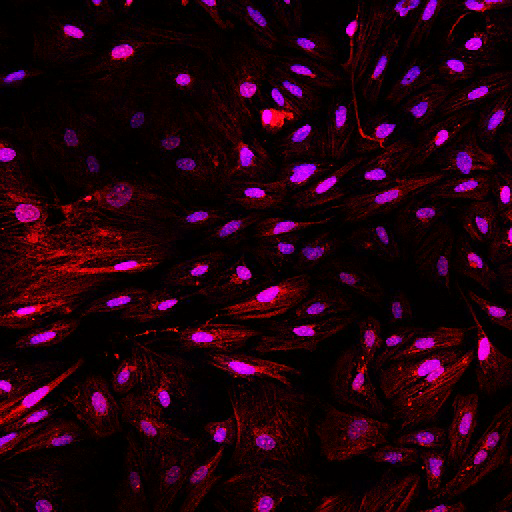

Supplement: Figure 2—source data 1. [file elife-75072-fig2-data1.zip › Figure 2-source data/figure2A/12h/merged.png]

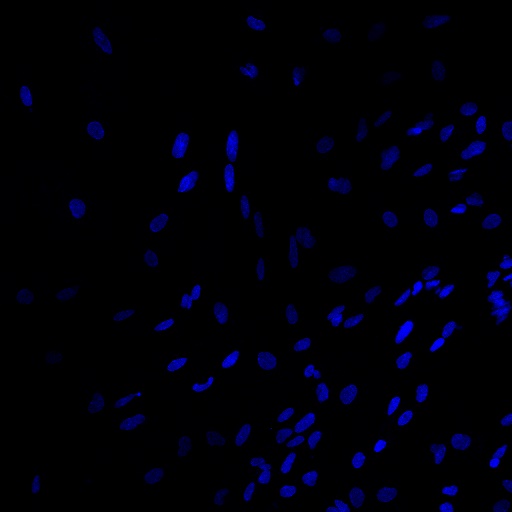

Supplement: Figure 2—source data 1. [file elife-75072-fig2-data1.zip › Figure 2-source data/figure2A/18h/DAPI.jpg]

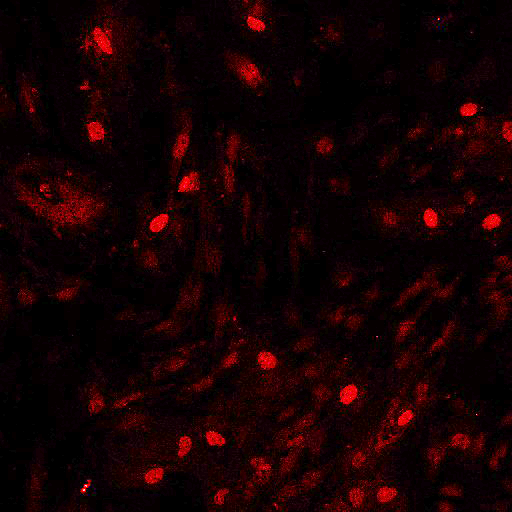

Supplement: Figure 2—source data 1. [file elife-75072-fig2-data1.zip › Figure 2-source data/figure2A/18h/IL-33.png]

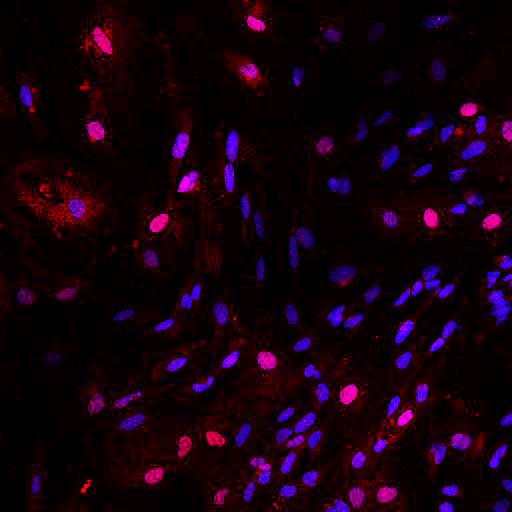

Supplement: Figure 2—source data 1. [file elife-75072-fig2-data1.zip › Figure 2-source data/figure2A/18h/merged.png]

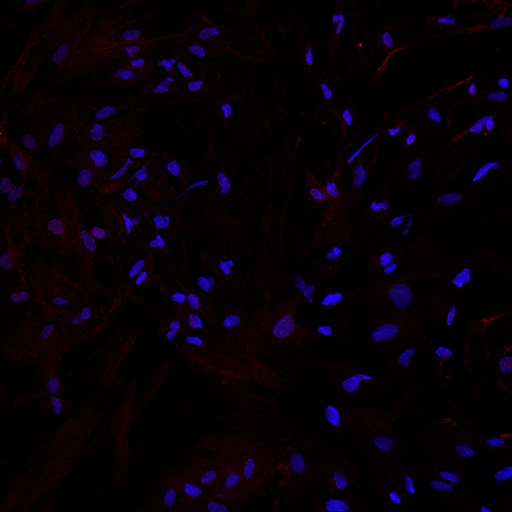

Supplement: Figure 2—source data 1. [file elife-75072-fig2-data1.zip › Figure 2-source data/figure2A/1h/DAPI.jpg]

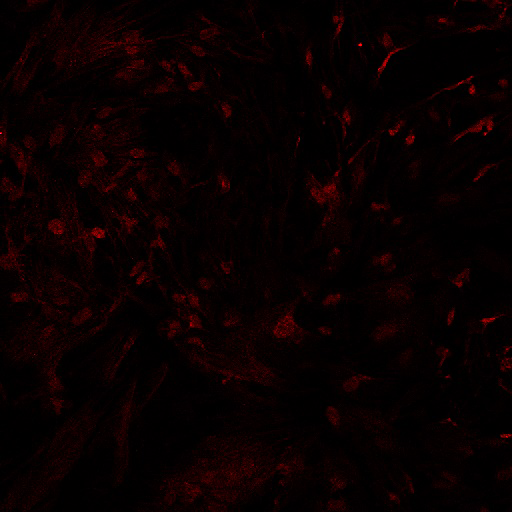

Supplement: Figure 2—source data 1. [file elife-75072-fig2-data1.zip › Figure 2-source data/figure2A/1h/IL-33.png]

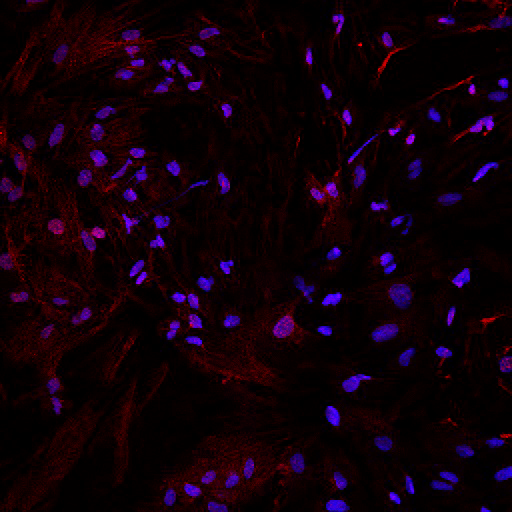

Supplement: Figure 2—source data 1. [file elife-75072-fig2-data1.zip › Figure 2-source data/figure2A/1h/merged.png]

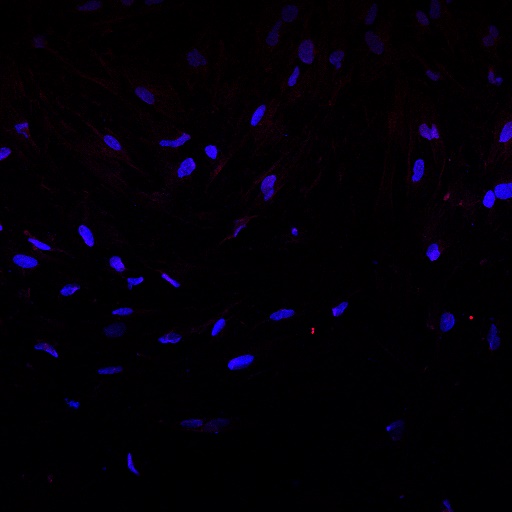

Supplement: Figure 2—source data 1. [file elife-75072-fig2-data1.zip › Figure 2-source data/figure2A/24h/DAPI.jpg]

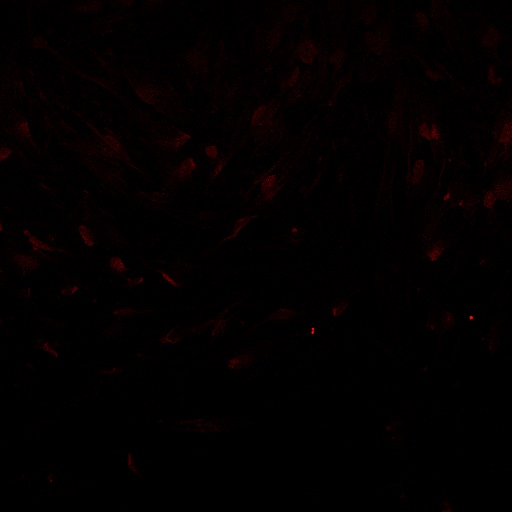

Supplement: Figure 2—source data 1. [file elife-75072-fig2-data1.zip › Figure 2-source data/figure2A/24h/IL-33.jpg]

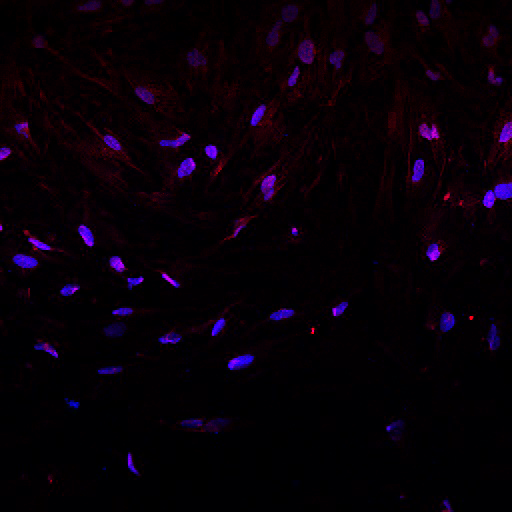

Supplement: Figure 2—source data 1. [file elife-75072-fig2-data1.zip › Figure 2-source data/figure2A/24h/merged.png]

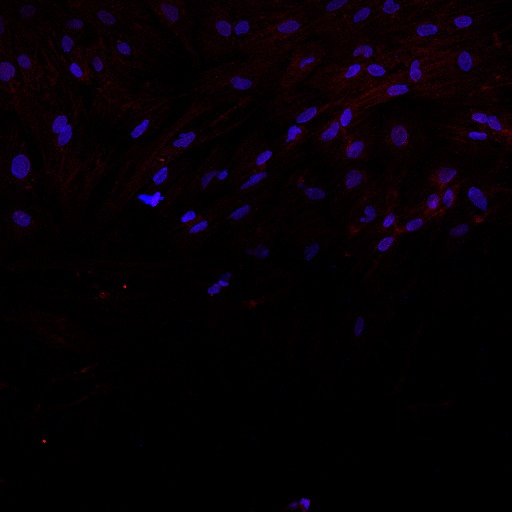

Supplement: Figure 2—source data 1. [file elife-75072-fig2-data1.zip › Figure 2-source data/figure2A/3h/DAPI.jpg]

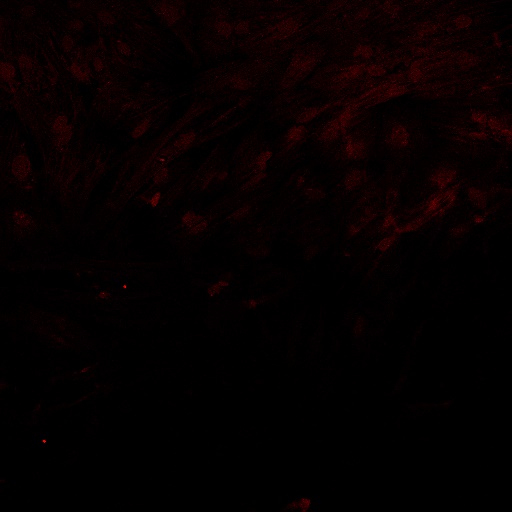

Supplement: Figure 2—source data 1. [file elife-75072-fig2-data1.zip › Figure 2-source data/figure2A/3h/IL-33.jpg]

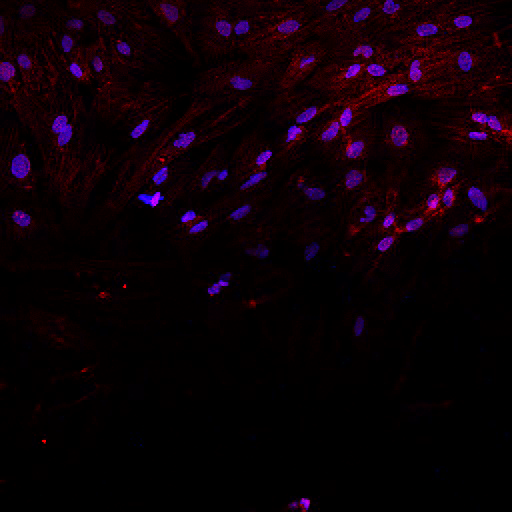

Supplement: Figure 2—source data 1. [file elife-75072-fig2-data1.zip › Figure 2-source data/figure2A/3h/merged.png]

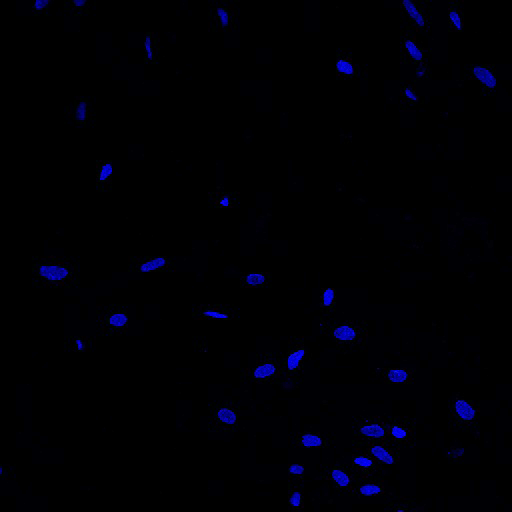

Supplement: Figure 2—source data 1. [file elife-75072-fig2-data1.zip › Figure 2-source data/figure2A/6h/DAPI.png]

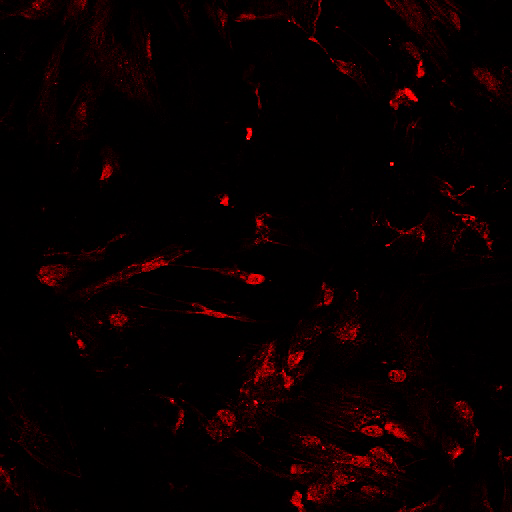

Supplement: Figure 2—source data 1. [file elife-75072-fig2-data1.zip › Figure 2-source data/figure2A/6h/IL-33.png]

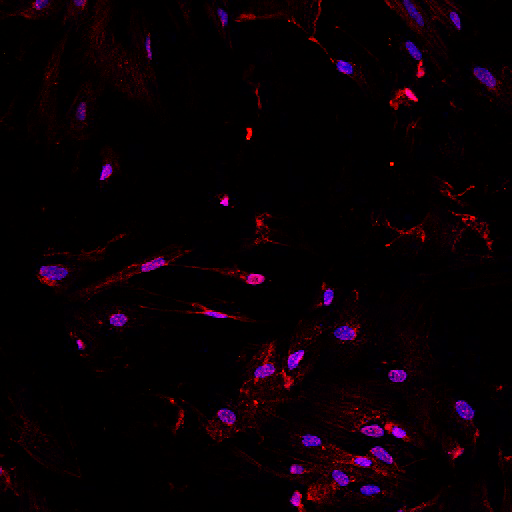

Supplement: Figure 2—source data 1. [file elife-75072-fig2-data1.zip › Figure 2-source data/figure2A/6h/megred.png]

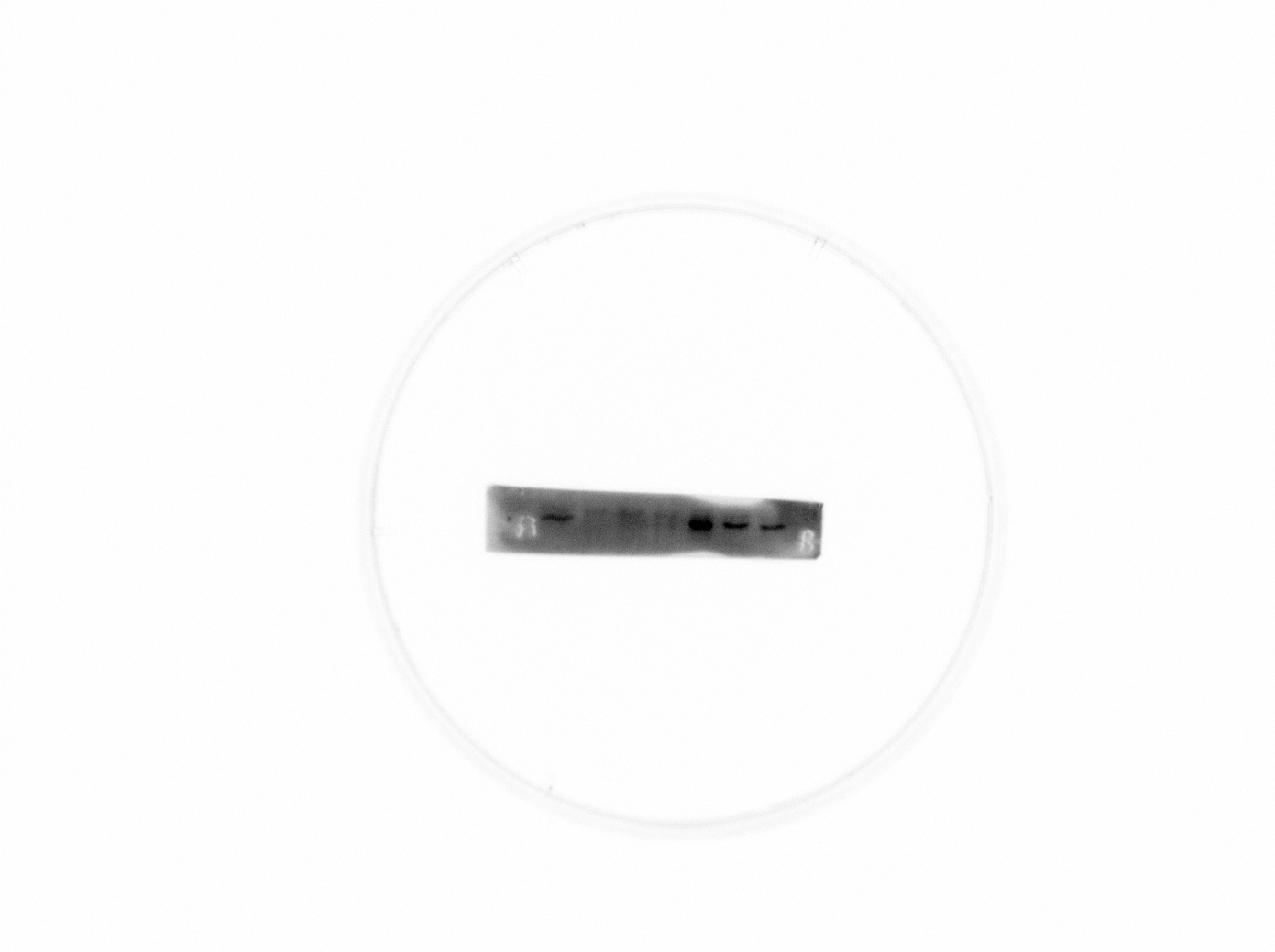

Supplement: Figure 2—source data 1. [file elife-75072-fig2-data1.zip › Figure 2-source data/figure2B/cytoplasmic IL-33.tif]

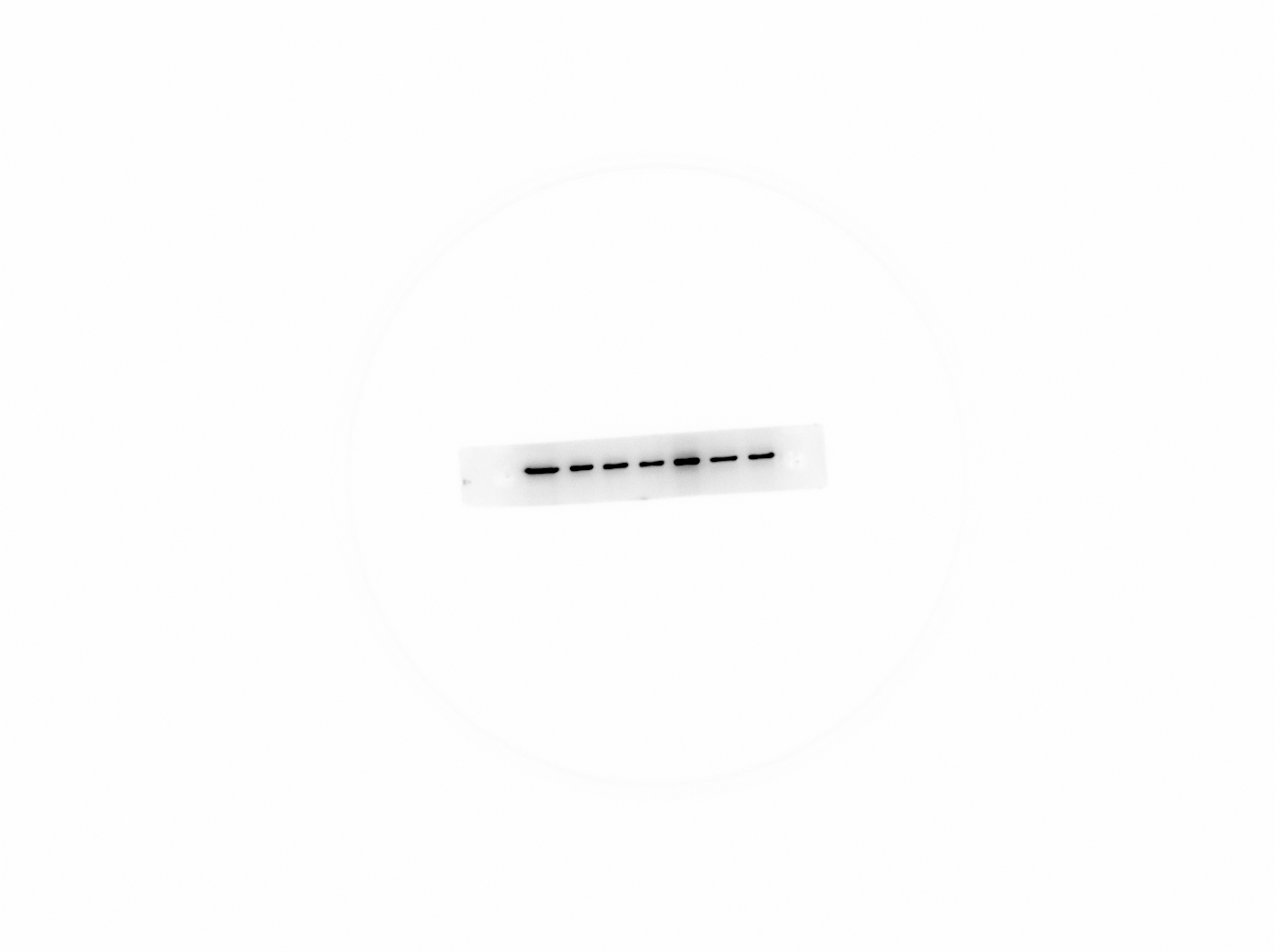

Supplement: Figure 2—source data 1. [file elife-75072-fig2-data1.zip › Figure 2-source data/figure2B/LaminB1.tif]

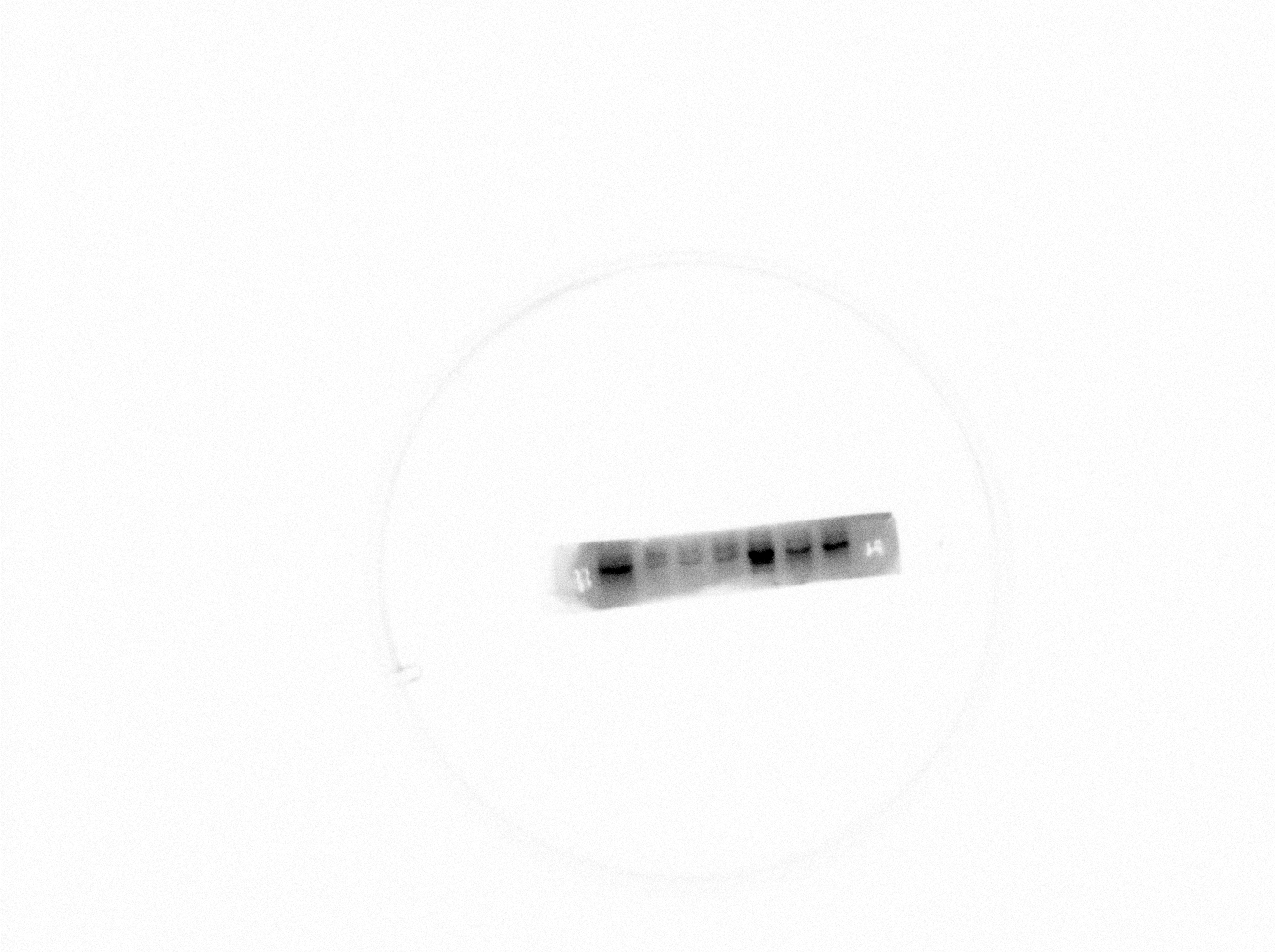

Supplement: Figure 2—source data 1. [file elife-75072-fig2-data1.zip › Figure 2-source data/figure2B/nuclear IL-33.tif]

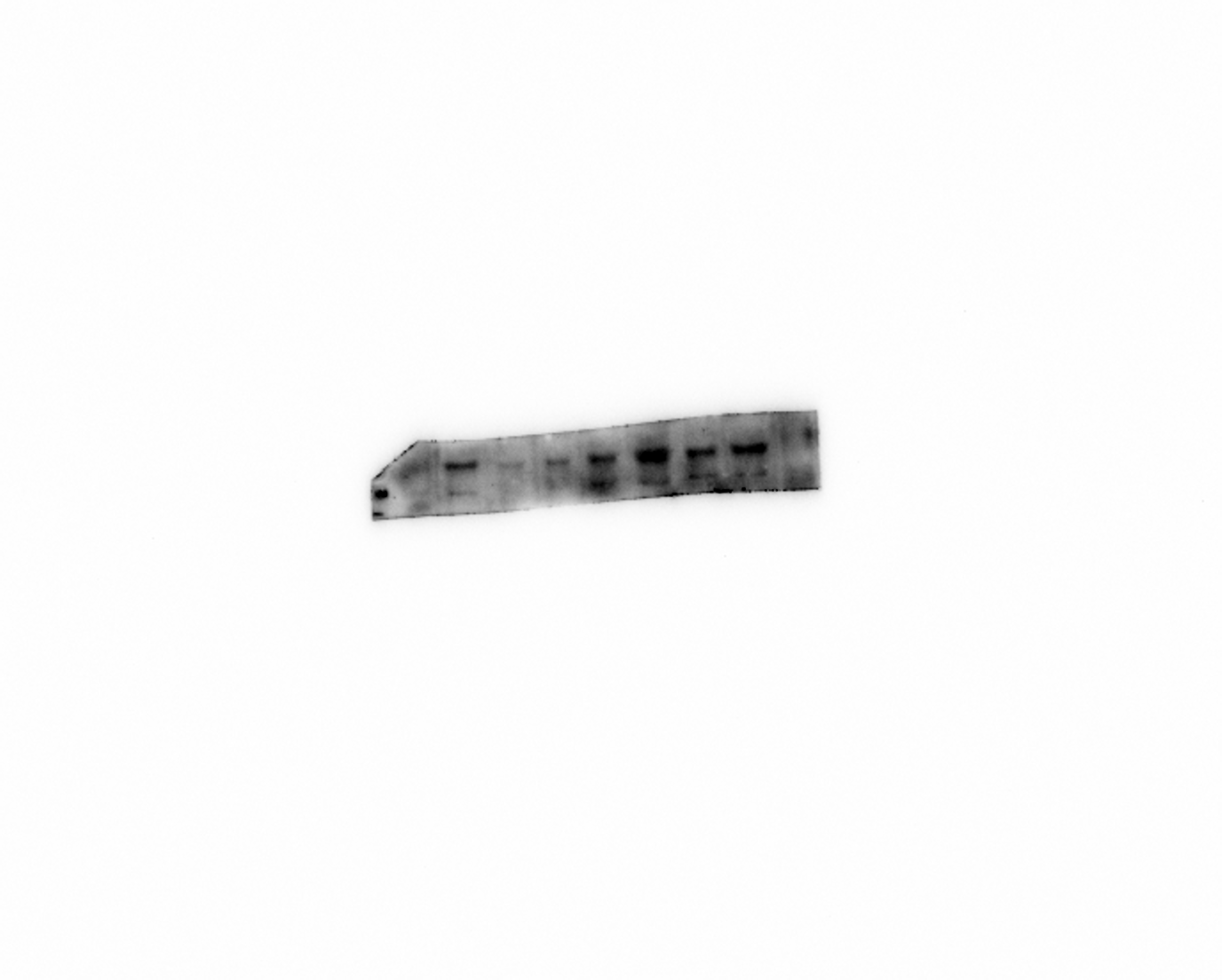

Supplement: Figure 2—source data 1. [file elife-75072-fig2-data1.zip › Figure 2-source data/figure2B/total IL-33/total IL-33.Tif]

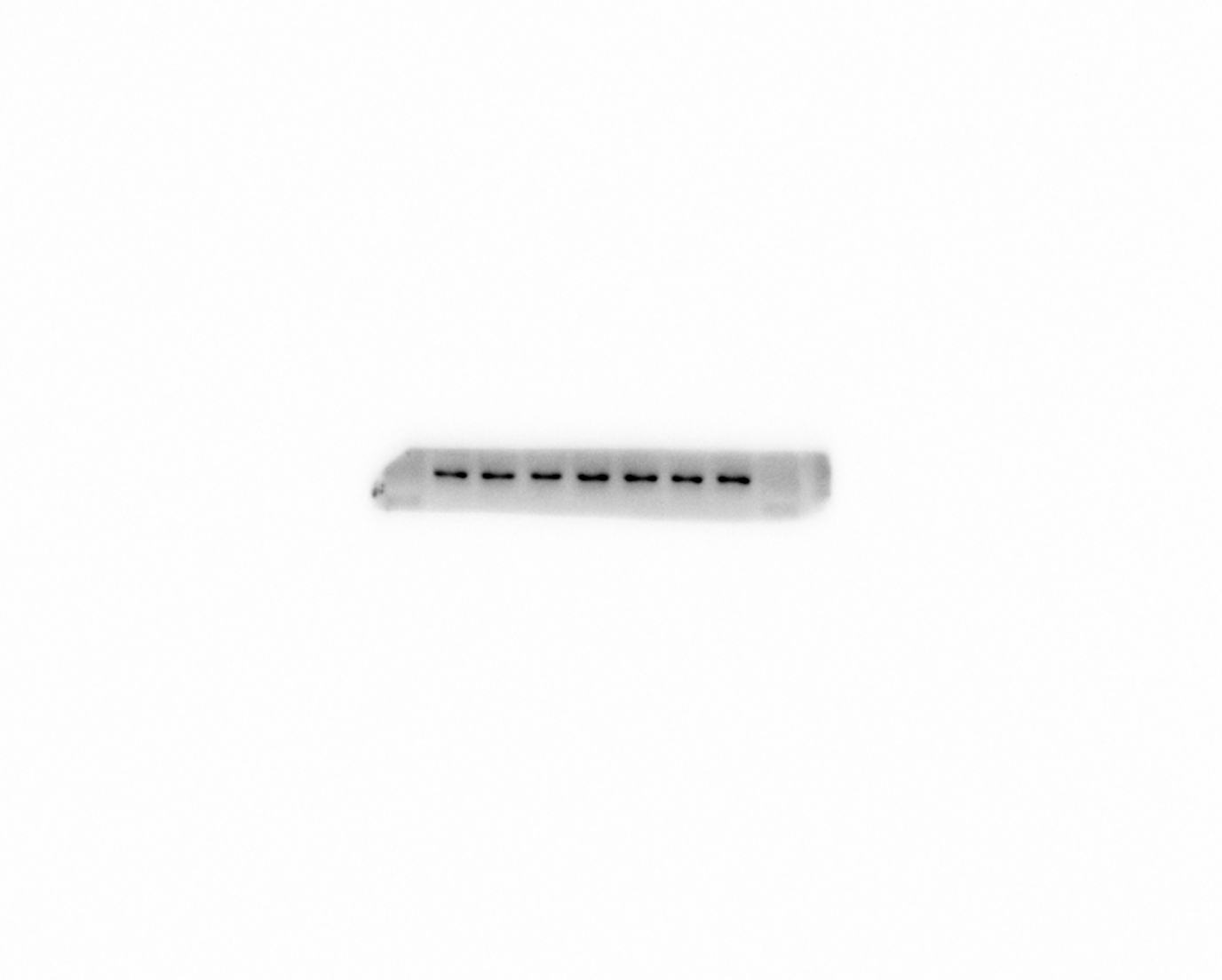

Supplement: Figure 2—source data 1. [file elife-75072-fig2-data1.zip › Figure 2-source data/figure2B/total IL-33/β-actin.Tif]

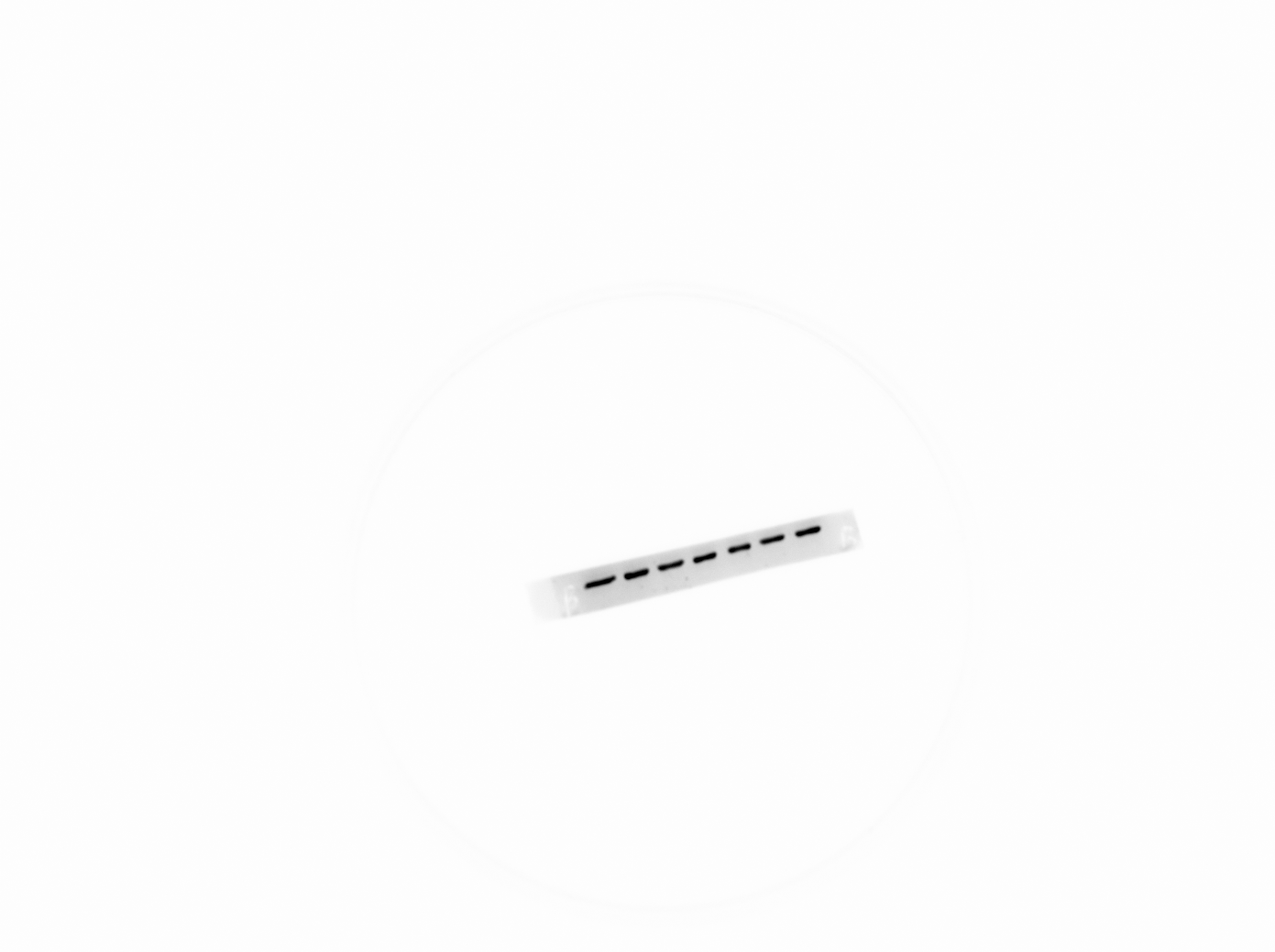

Supplement: Figure 2—source data 1. [file elife-75072-fig2-data1.zip › Figure 2-source data/figure2B/β-actin.tif]

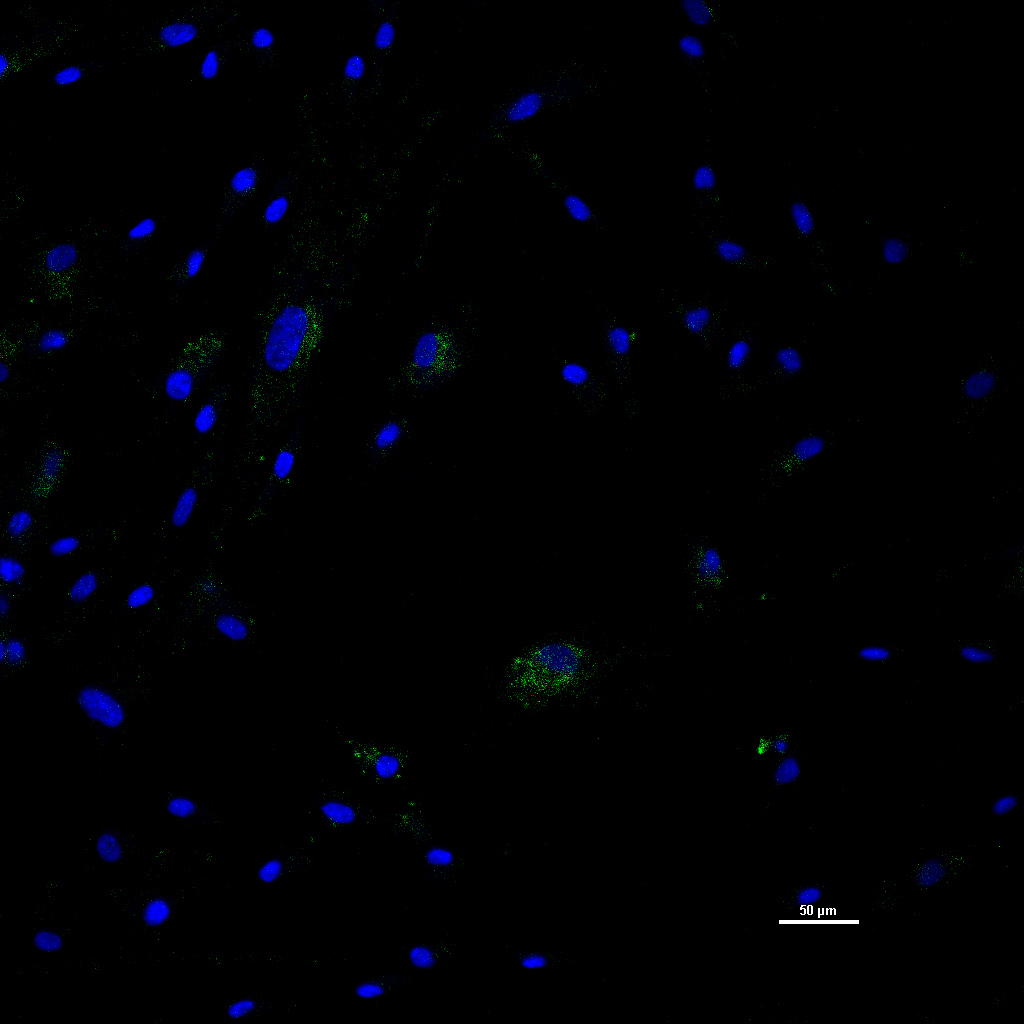

Supplement: Figure 3—source data 1. [file elife-75072-fig3-data1.zip › Figure 3-source data/figure3A/LPS(0.5h)/lps 30min-merge-1.tif]

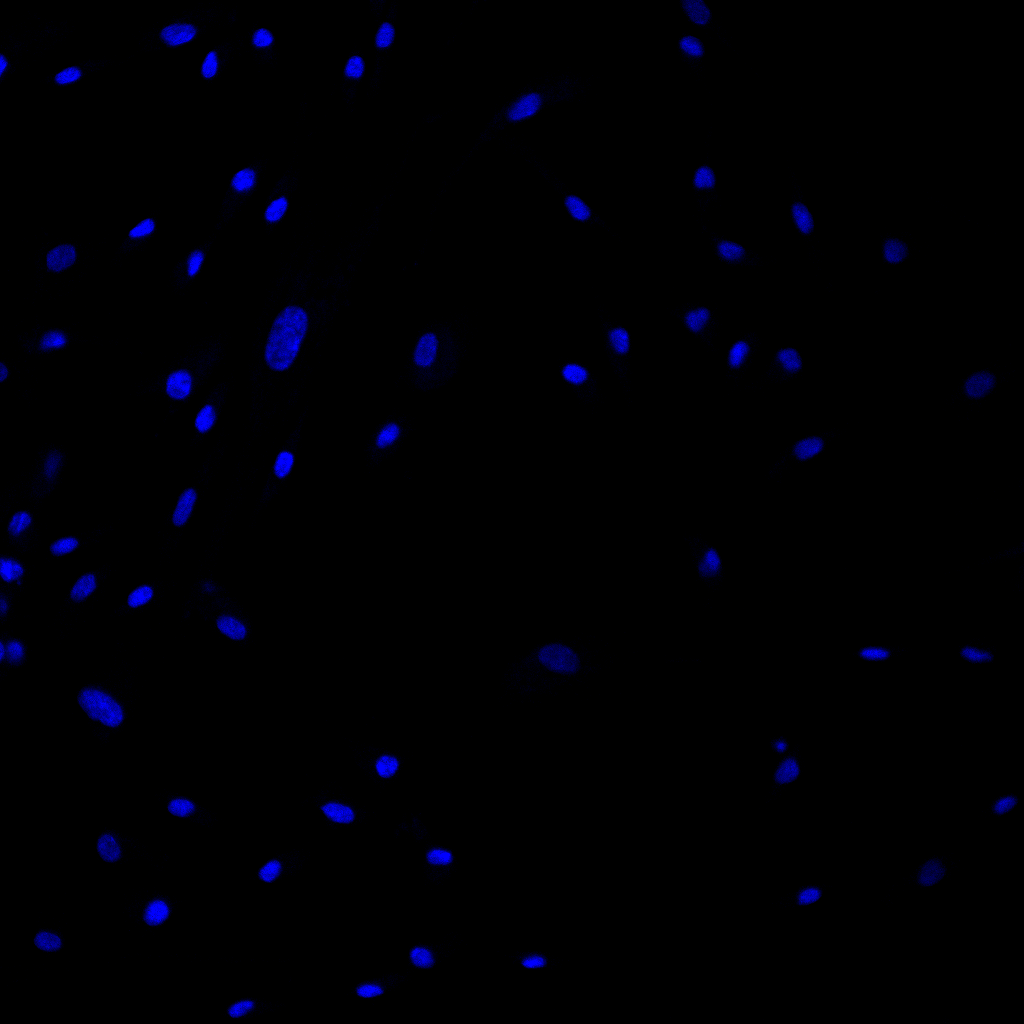

Supplement: Figure 3—source data 1. [file elife-75072-fig3-data1.zip › Figure 3-source data/figure3A/LPS(0.5h)/lps 30min-merge-1c1.tif]

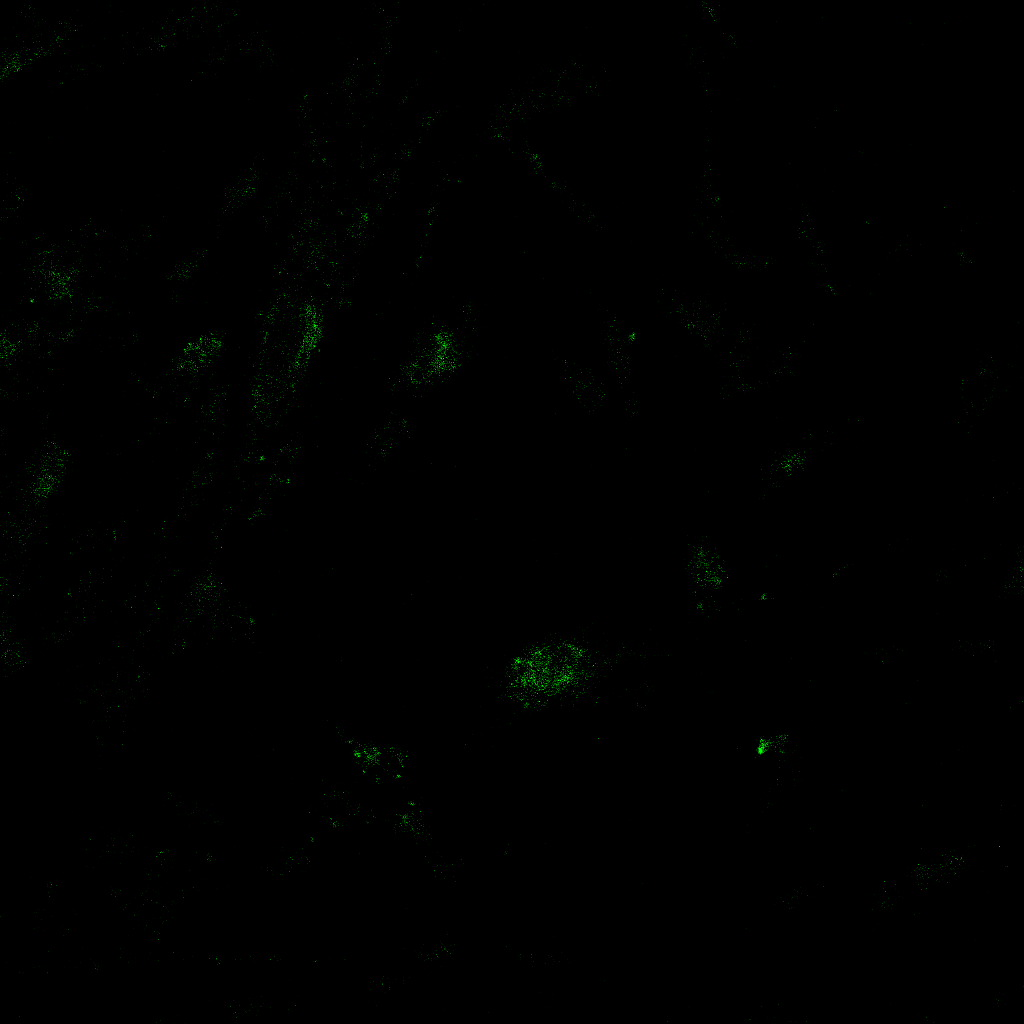

Supplement: Figure 3—source data 1. [file elife-75072-fig3-data1.zip › Figure 3-source data/figure3A/LPS(0.5h)/lps 30min-merge-1c2.tif]

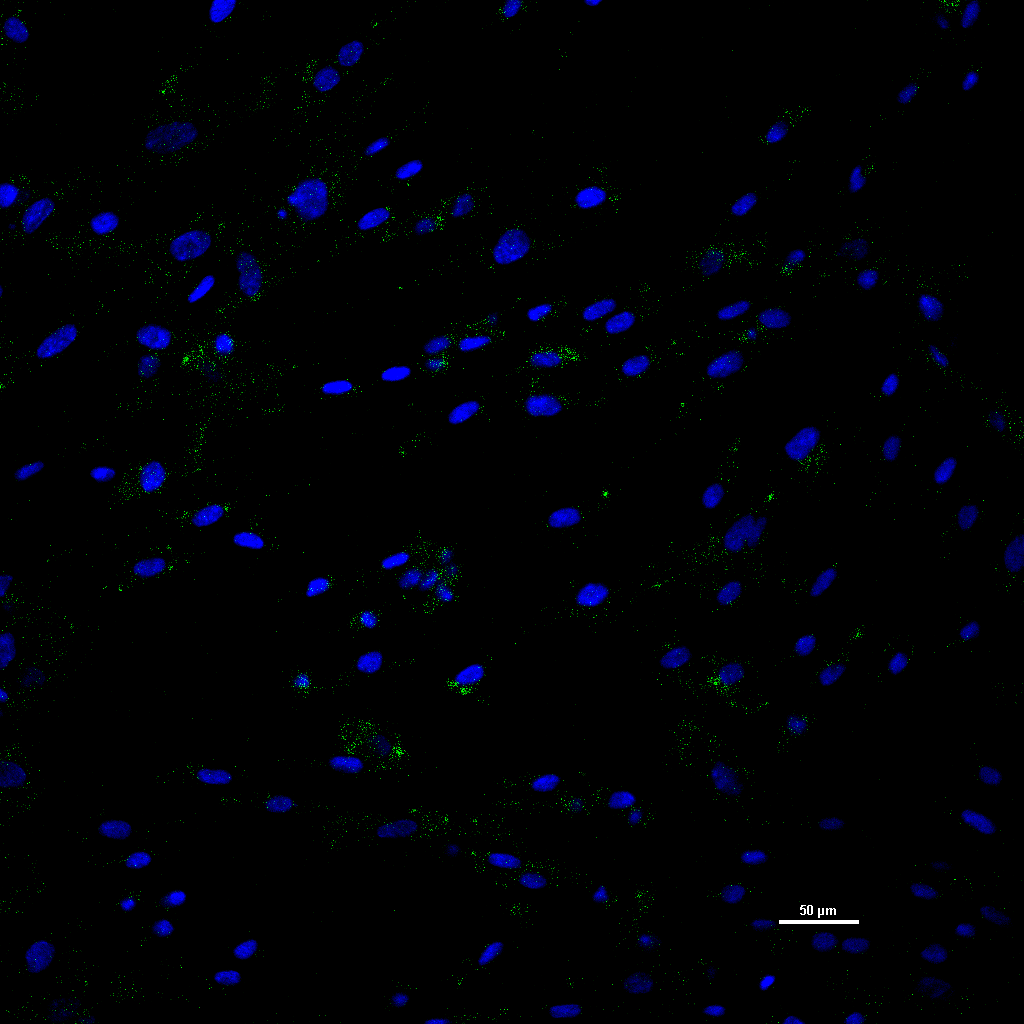

Supplement: Figure 3—source data 1. [file elife-75072-fig3-data1.zip › Figure 3-source data/figure3A/LPS(0h)/con-merge-1.tif]

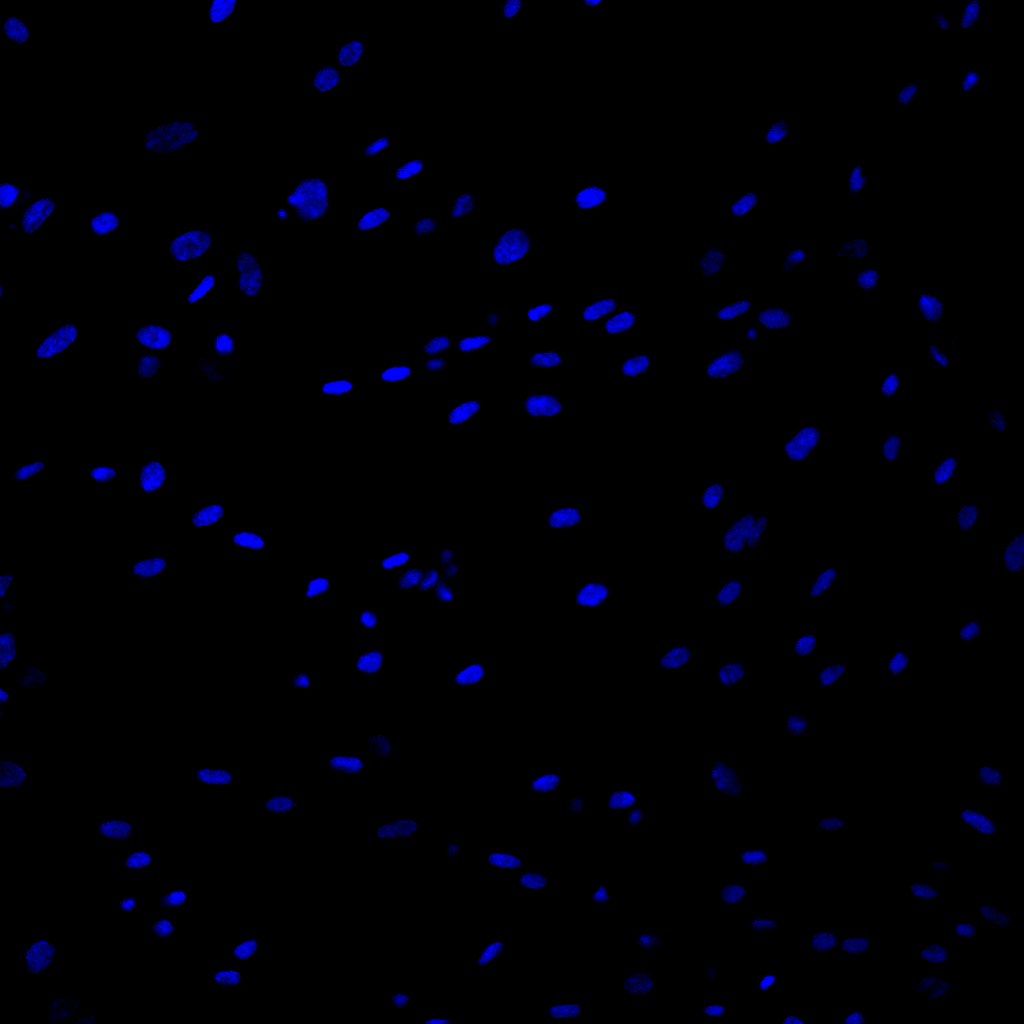

Supplement: Figure 3—source data 1. [file elife-75072-fig3-data1.zip › Figure 3-source data/figure3A/LPS(0h)/con-merge-1c1.tif]

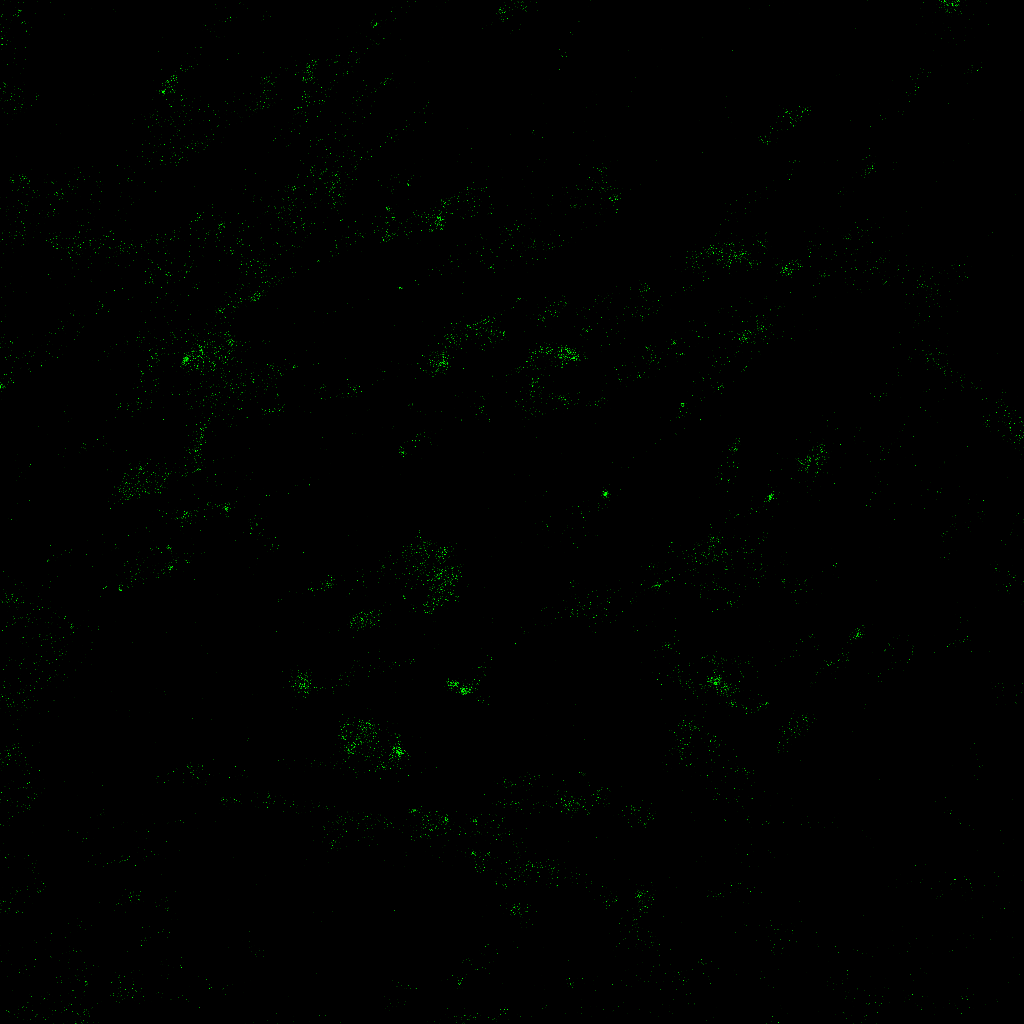

Supplement: Figure 3—source data 1. [file elife-75072-fig3-data1.zip › Figure 3-source data/figure3A/LPS(0h)/con-merge-1c2.tif]

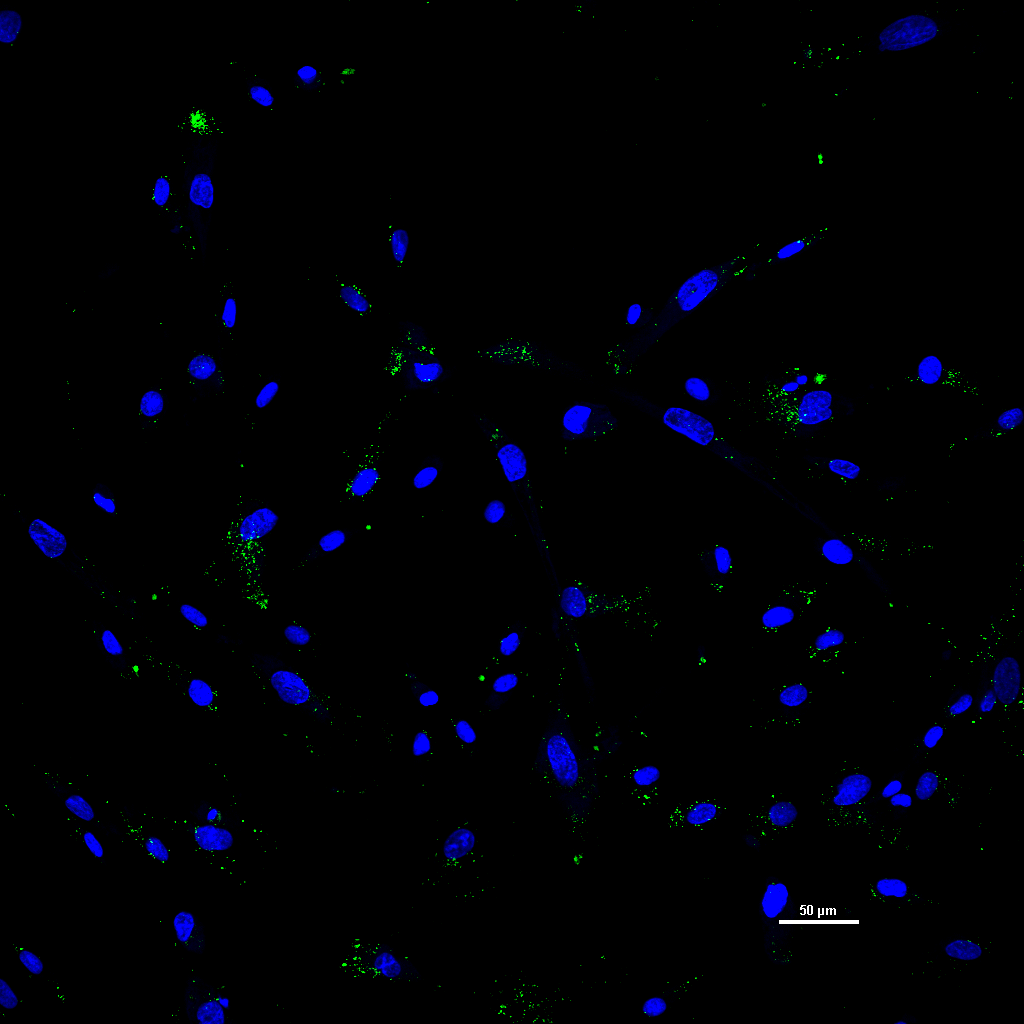

Supplement: Figure 3—source data 1. [file elife-75072-fig3-data1.zip › Figure 3-source data/figure3A/LPS(1h)/lps 1h-merge-1.tif]

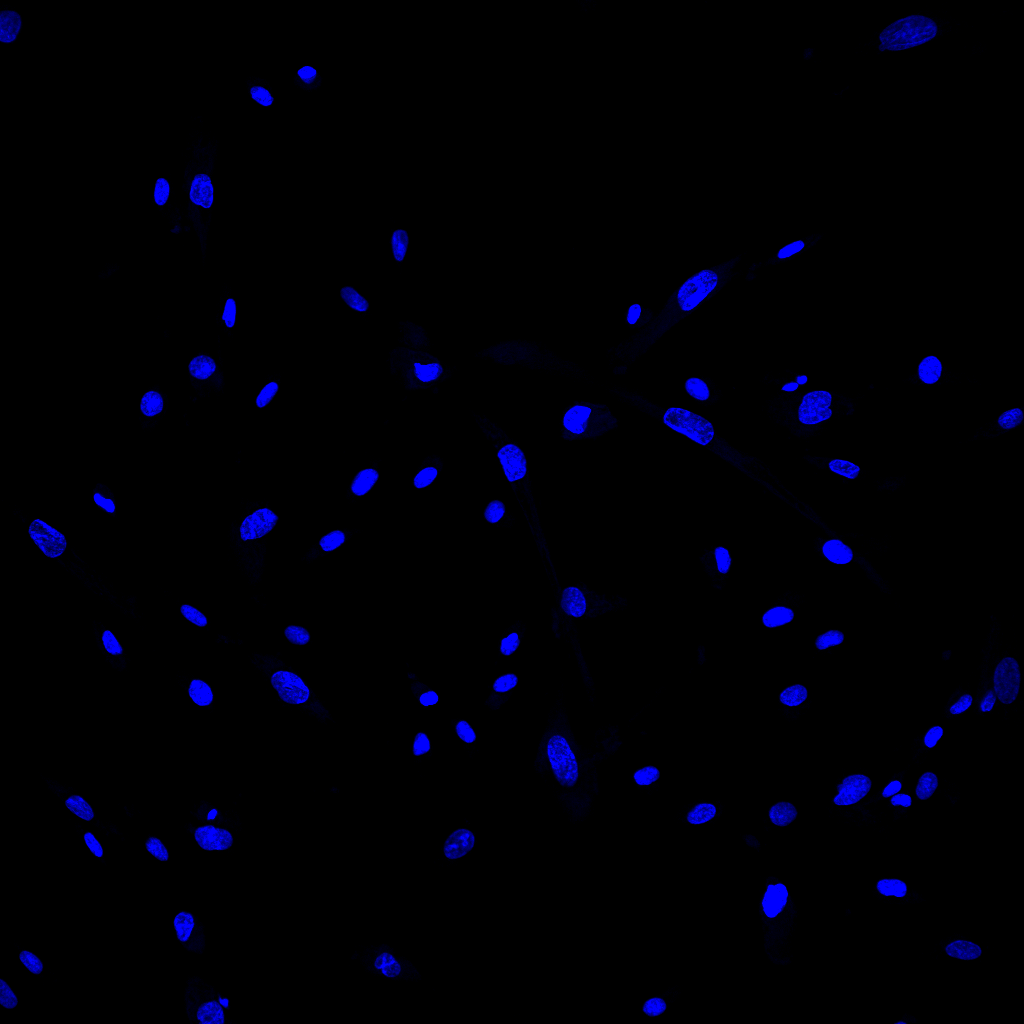

Supplement: Figure 3—source data 1. [file elife-75072-fig3-data1.zip › Figure 3-source data/figure3A/LPS(1h)/lps 1h-merge-1c1.tif]

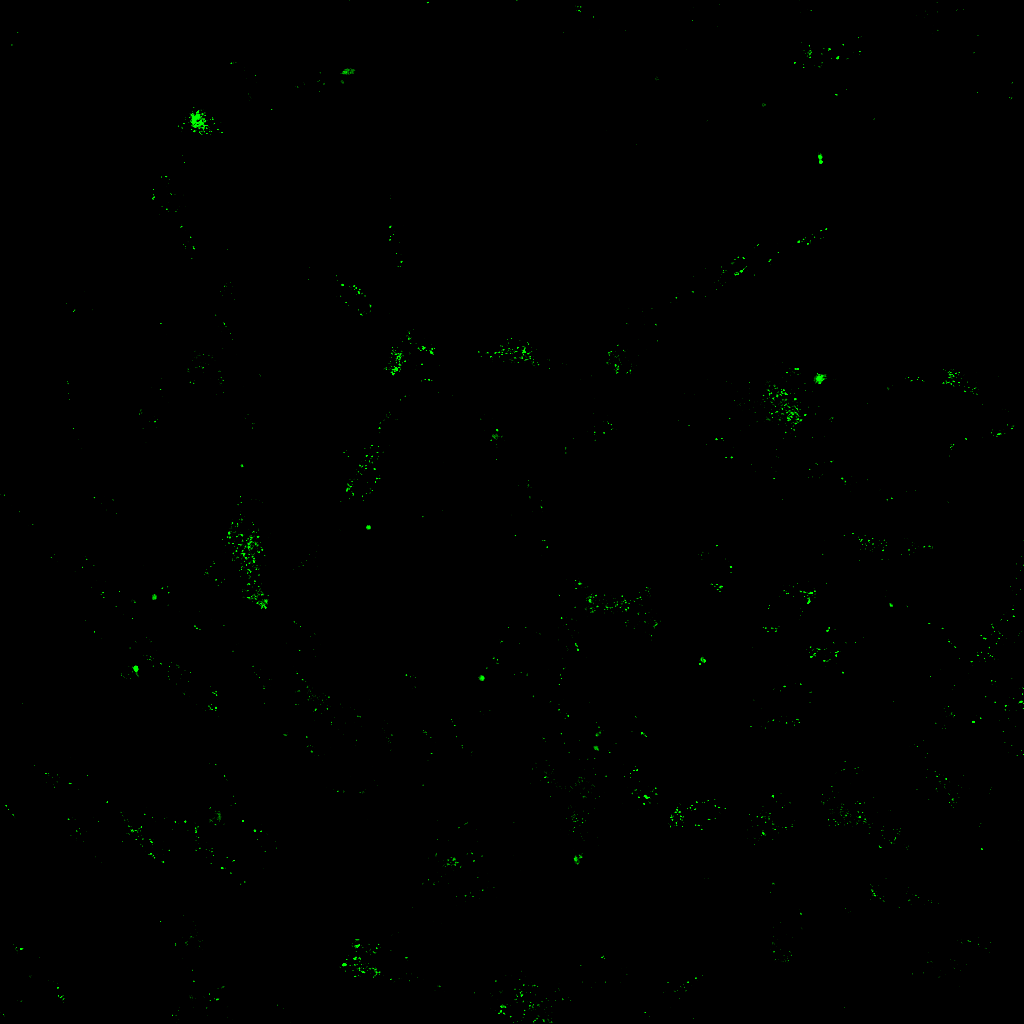

Supplement: Figure 3—source data 1. [file elife-75072-fig3-data1.zip › Figure 3-source data/figure3A/LPS(1h)/lps 1h-merge-1c2.tif]

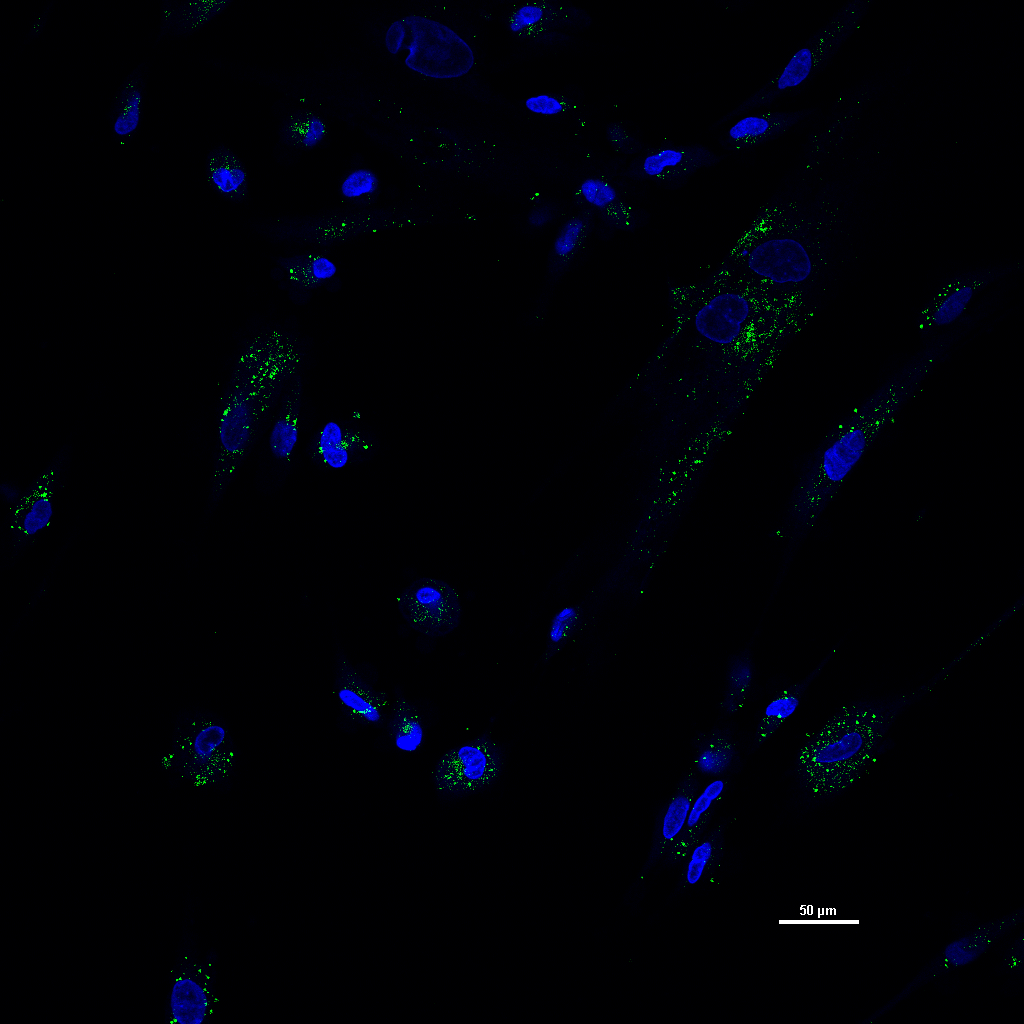

Supplement: Figure 3—source data 1. [file elife-75072-fig3-data1.zip › Figure 3-source data/figure3A/LPS(3h)/lps 3h-merge 1.tif]

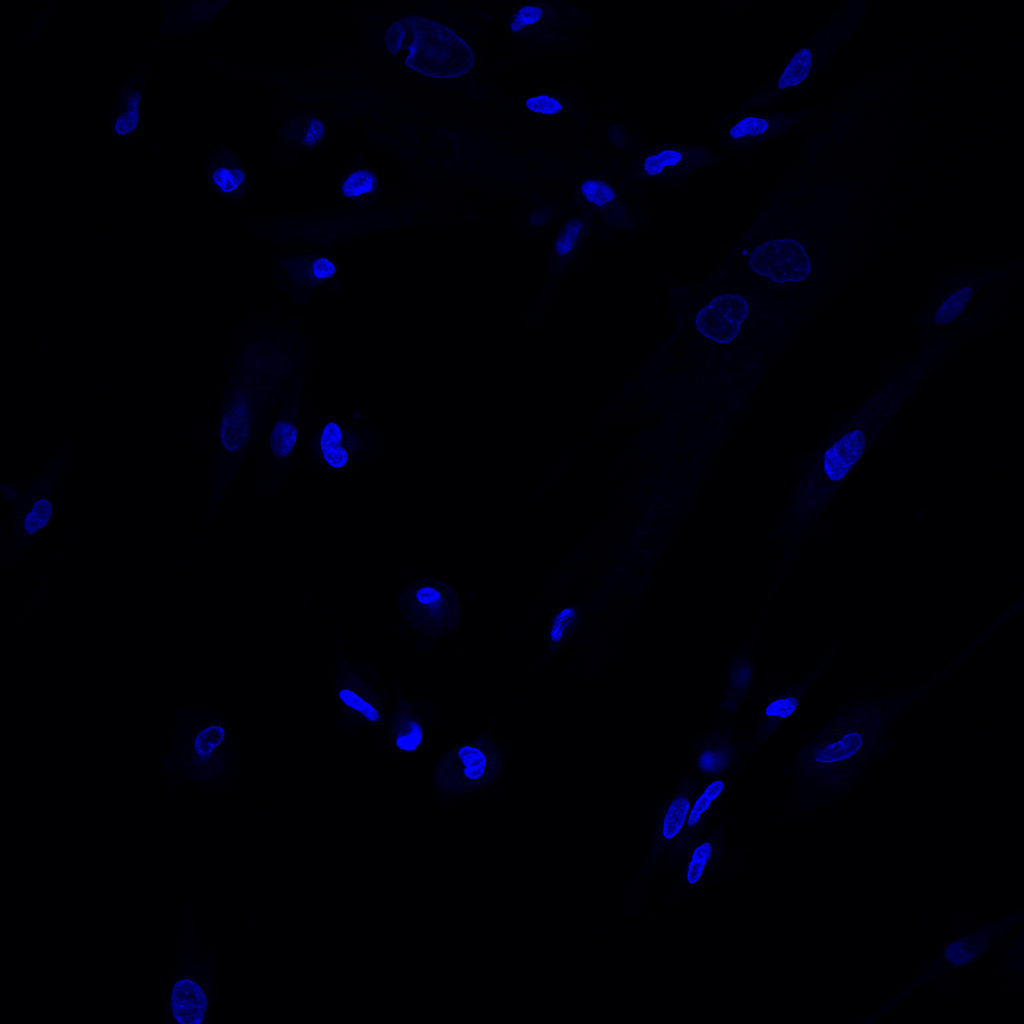

Supplement: Figure 3—source data 1. [file elife-75072-fig3-data1.zip › Figure 3-source data/figure3A/LPS(3h)/lps 3h-merge 1c1.tif]

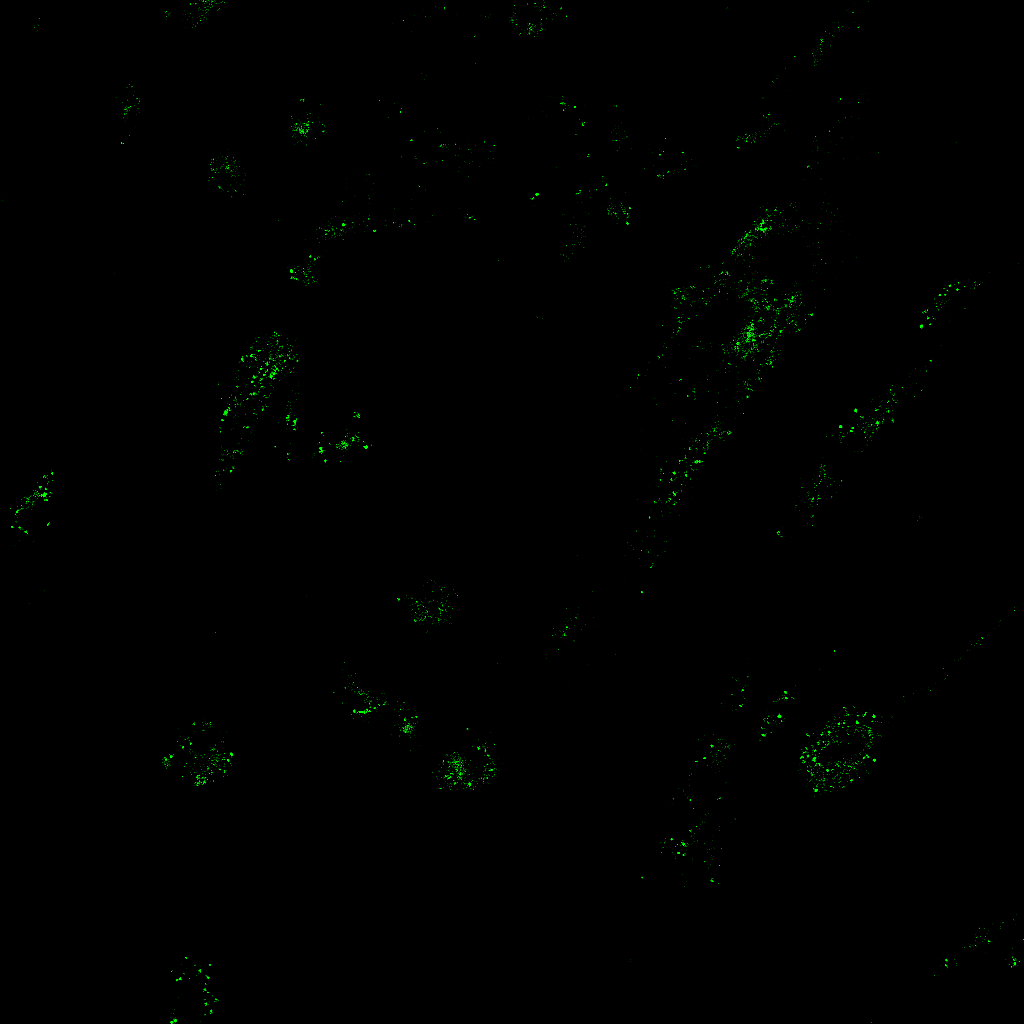

Supplement: Figure 3—source data 1. [file elife-75072-fig3-data1.zip › Figure 3-source data/figure3A/LPS(3h)/lps 3h-merge 1c2.tif]

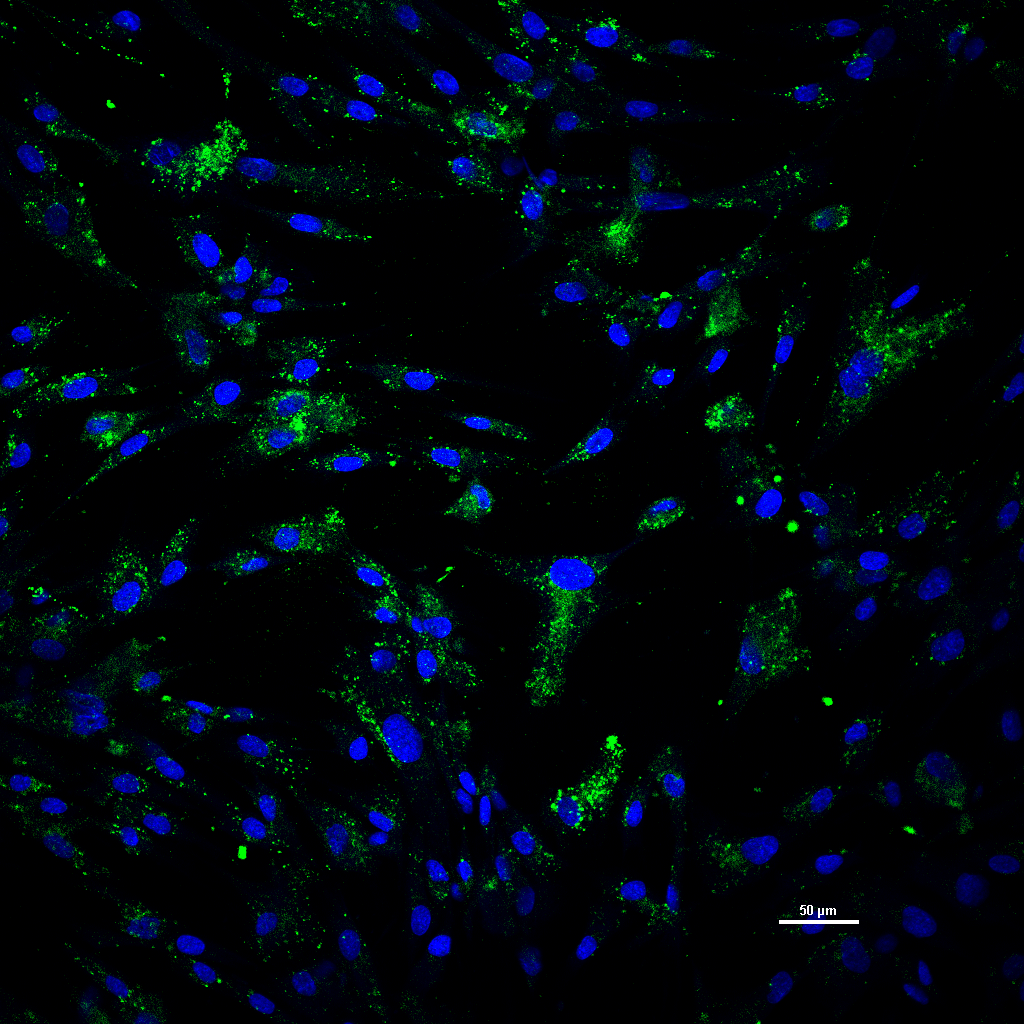

Supplement: Figure 3—source data 1. [file elife-75072-fig3-data1.zip › Figure 3-source data/figure3A/LPS(6h)/lps 6h-merge-1.tif]

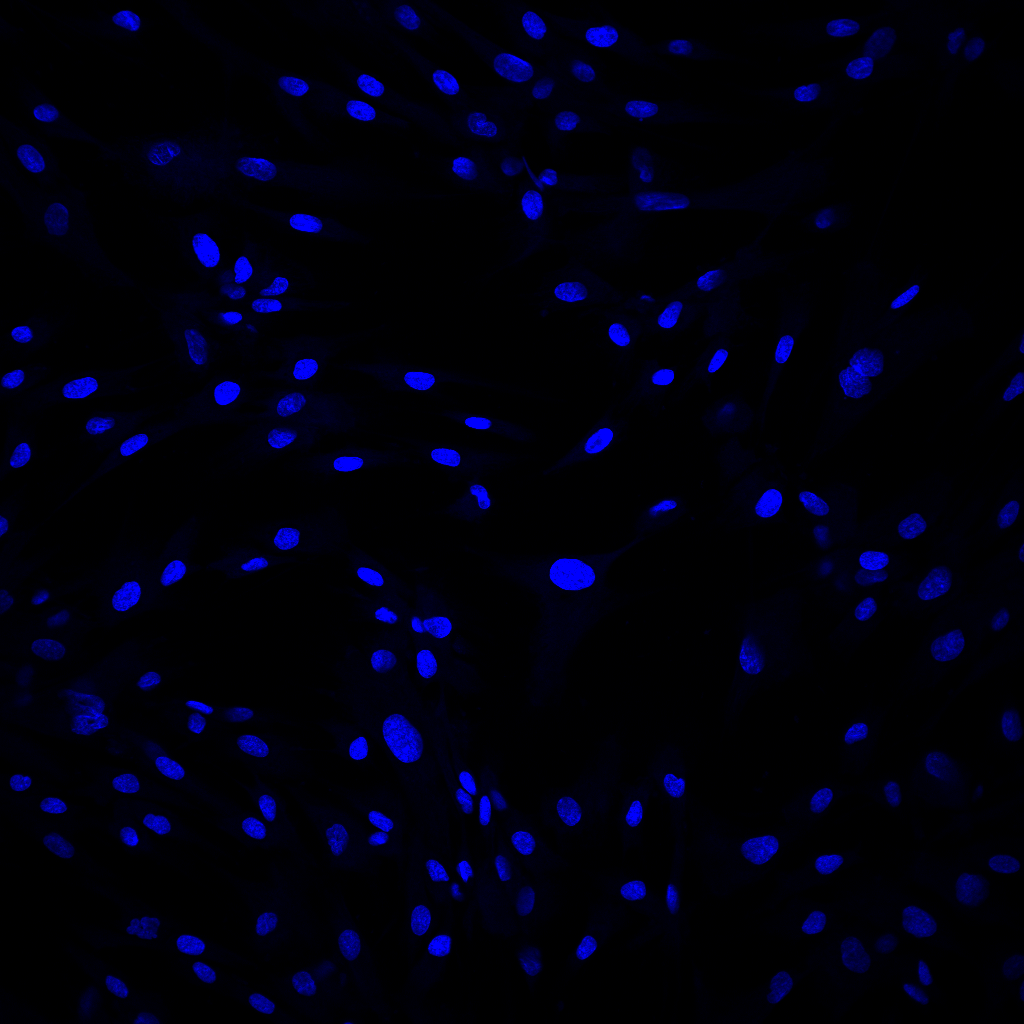

Supplement: Figure 3—source data 1. [file elife-75072-fig3-data1.zip › Figure 3-source data/figure3A/LPS(6h)/lps 6h-merge-1c1.tif]

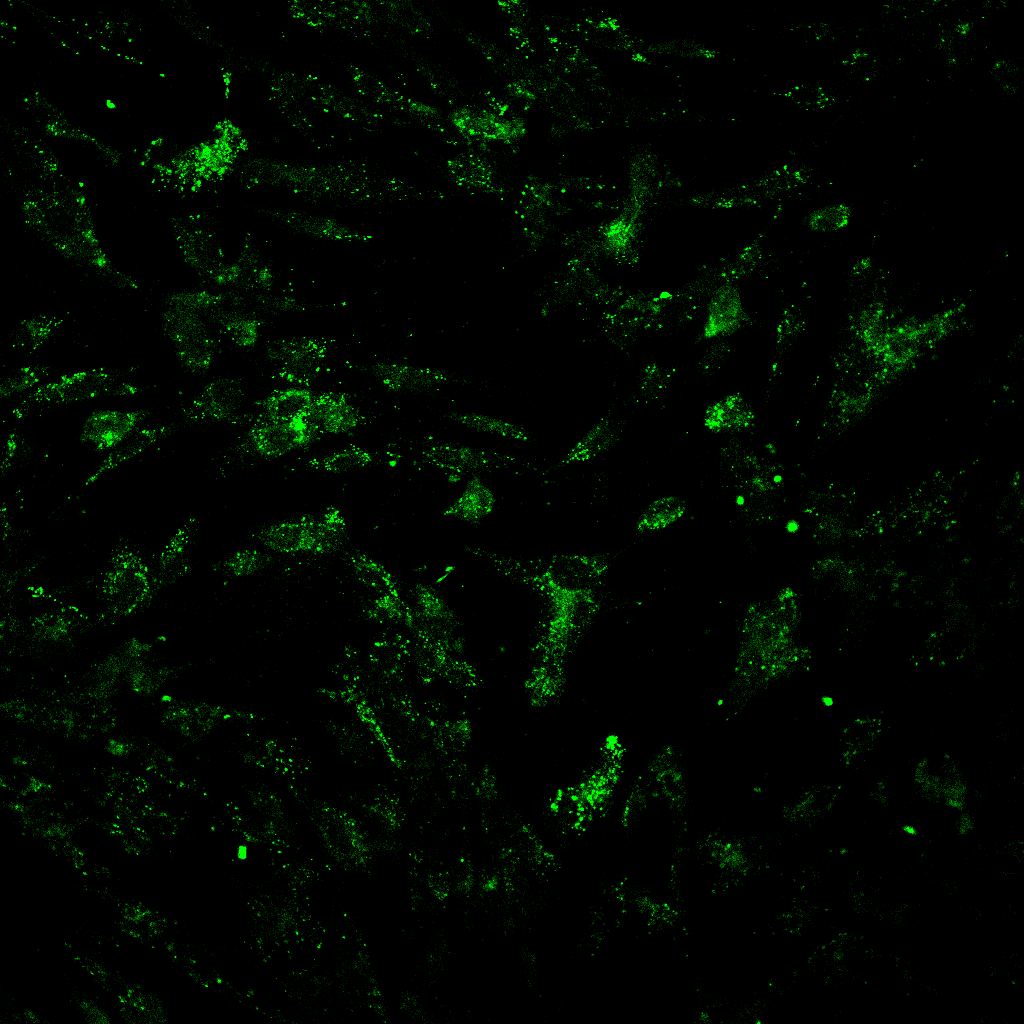

Supplement: Figure 3—source data 1. [file elife-75072-fig3-data1.zip › Figure 3-source data/figure3A/LPS(6h)/lps 6h-merge-1c2.tif]

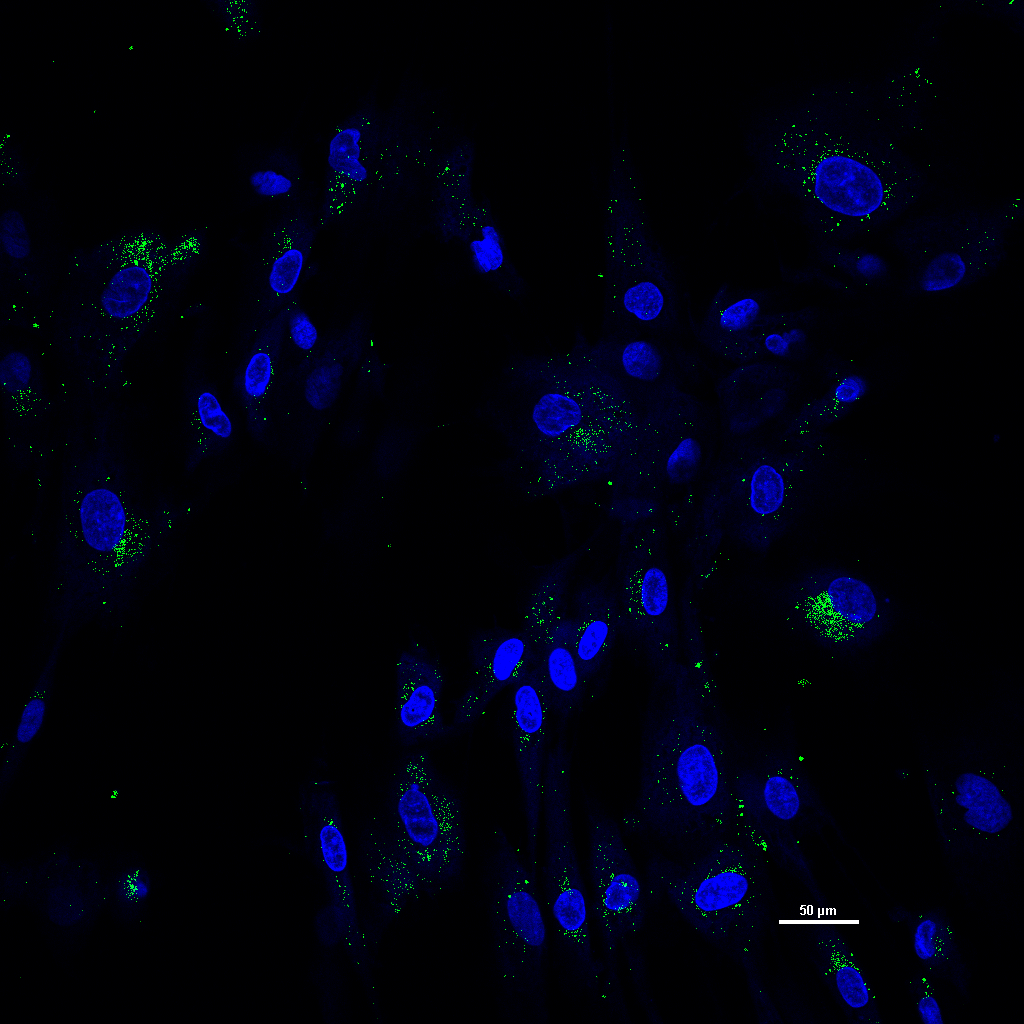

Supplement: Figure 3—source data 1. [file elife-75072-fig3-data1.zip › Figure 3-source data/figure3A/LPS+IL-33 siRNA(0.5h)/lps 30min+IL-33 siRNA-merge 1.tif]

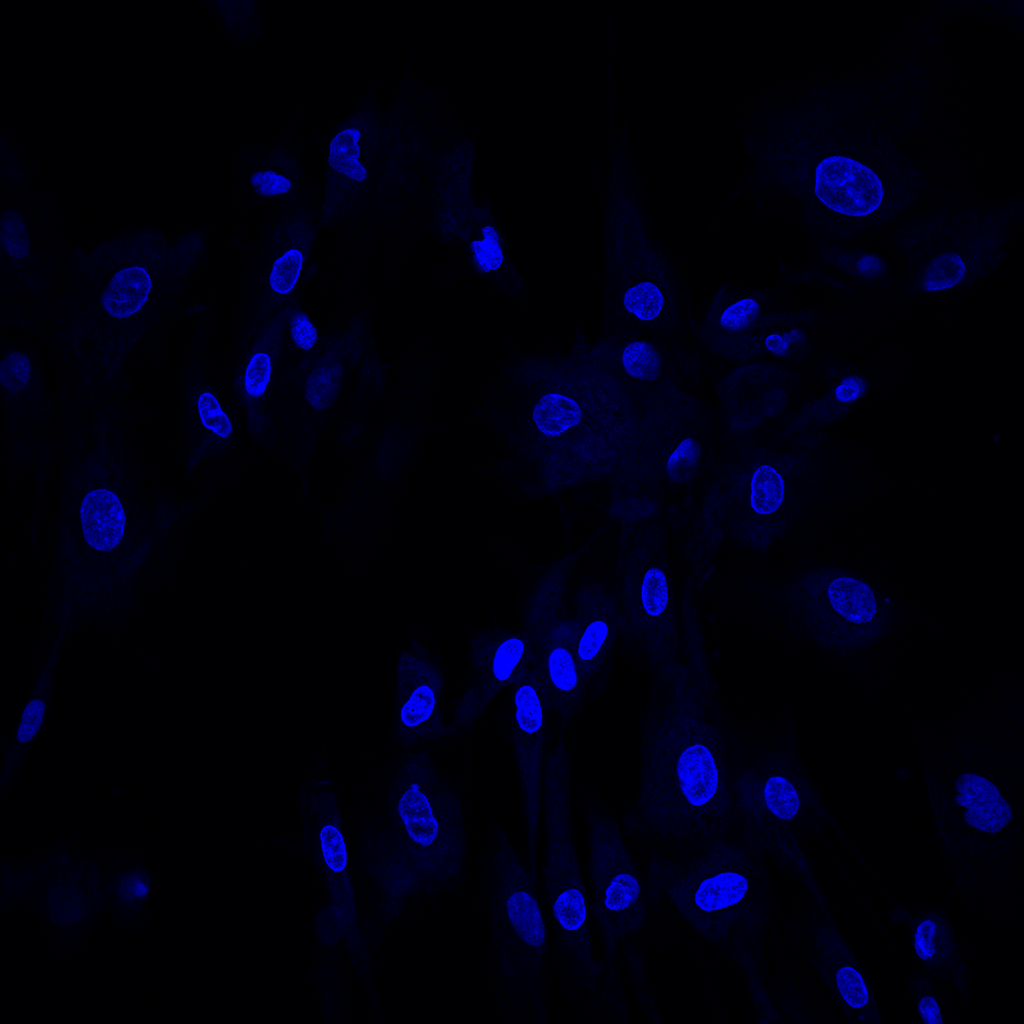

Supplement: Figure 3—source data 1. [file elife-75072-fig3-data1.zip › Figure 3-source data/figure3A/LPS+IL-33 siRNA(0.5h)/lps 30min+IL-33 siRNA-merge 1c1.tif]

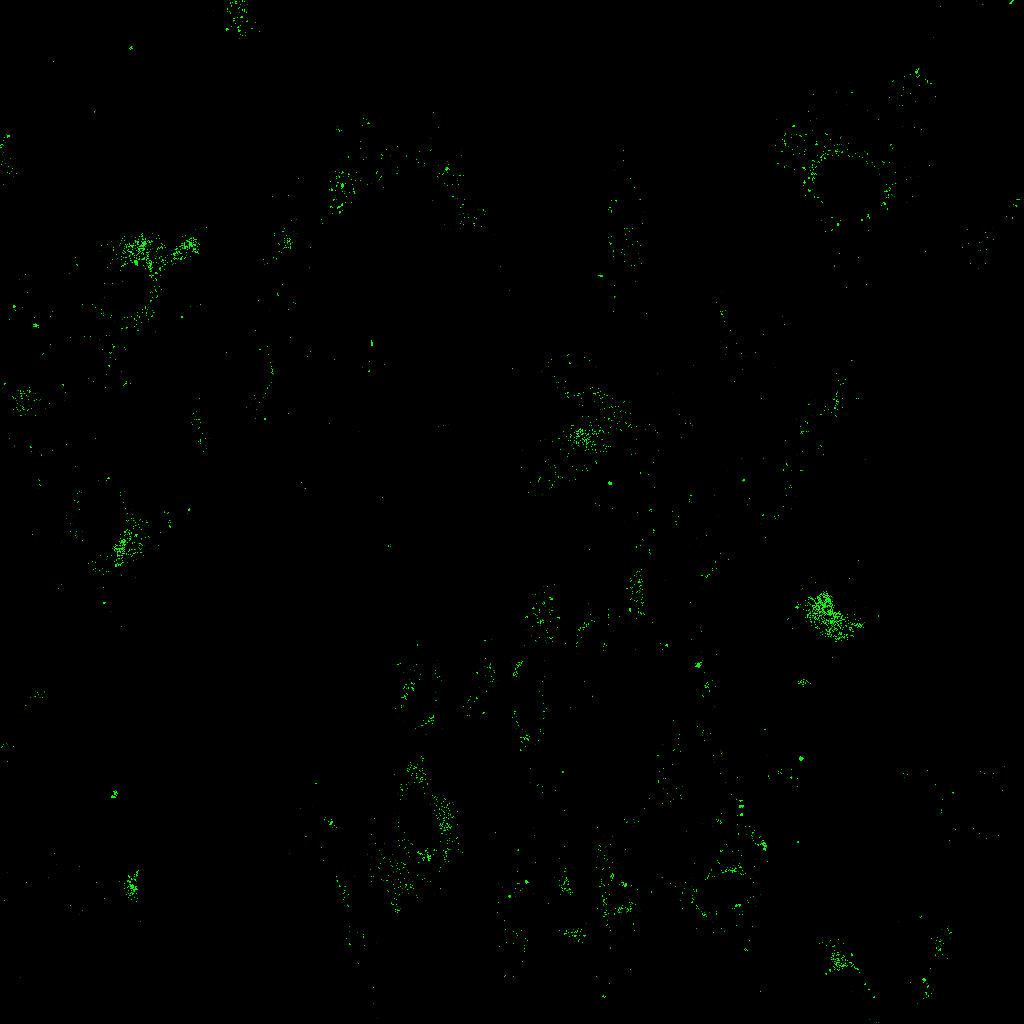

Supplement: Figure 3—source data 1. [file elife-75072-fig3-data1.zip › Figure 3-source data/figure3A/LPS+IL-33 siRNA(0.5h)/lps 30min+IL-33 siRNA-merge 1c2.tif]

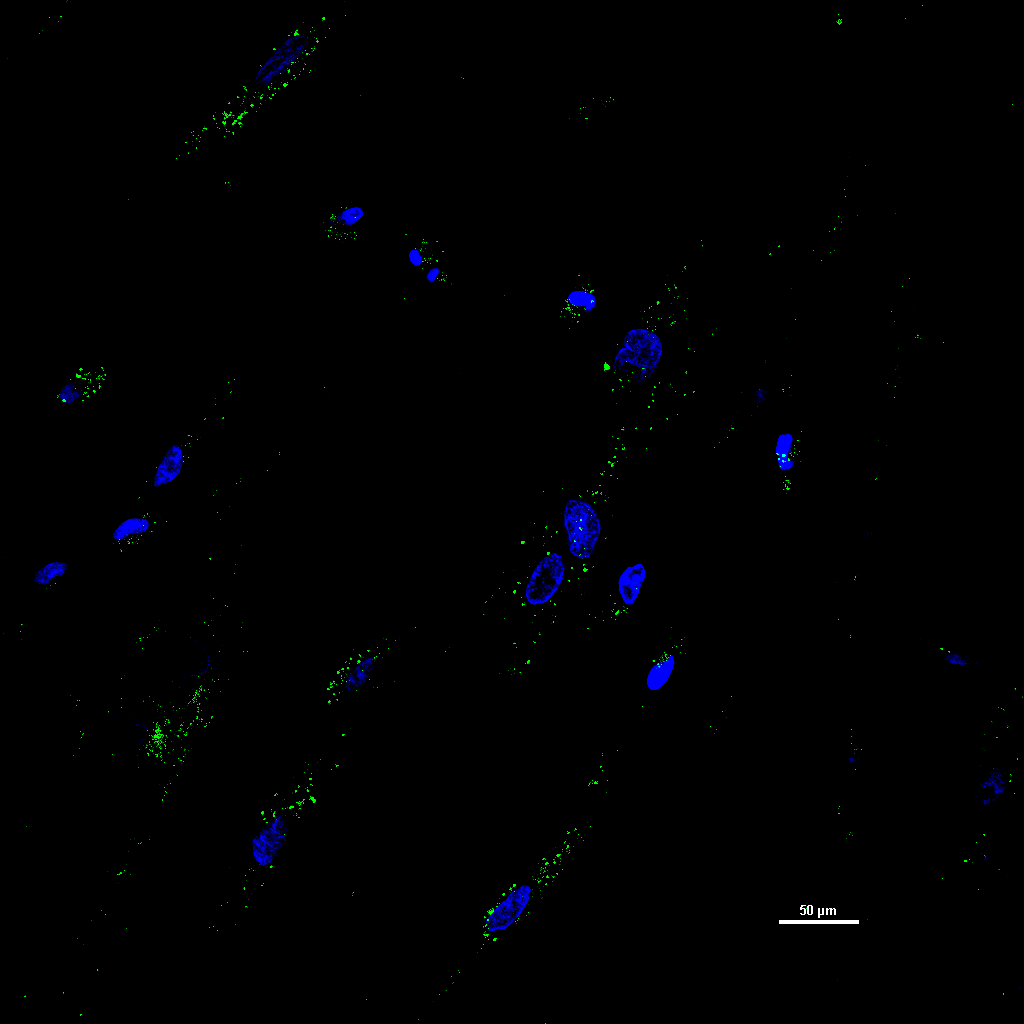

Supplement: Figure 3—source data 1. [file elife-75072-fig3-data1.zip › Figure 3-source data/figure3A/LPS+IL-33 siRNA(0h)/si merge 1.tif]

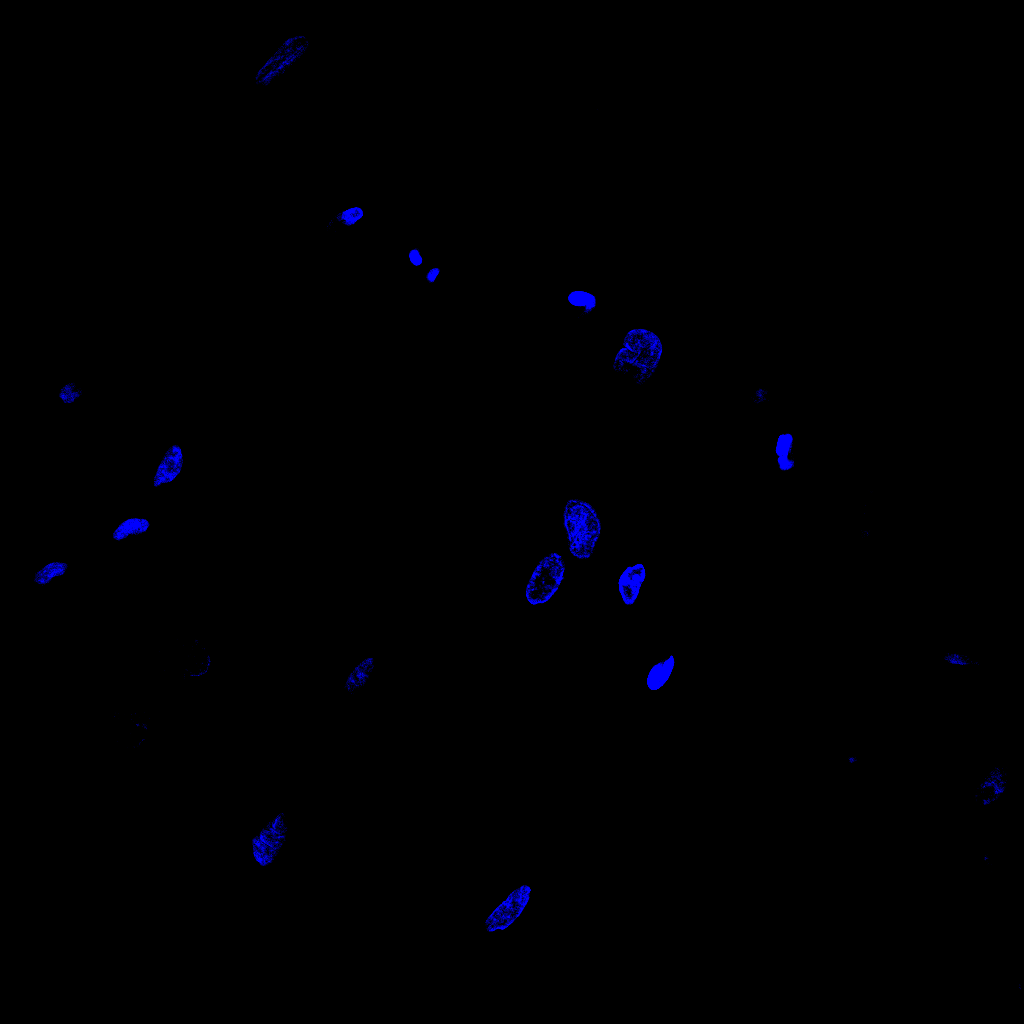

Supplement: Figure 3—source data 1. [file elife-75072-fig3-data1.zip › Figure 3-source data/figure3A/LPS+IL-33 siRNA(0h)/si merge 1c1.tif]

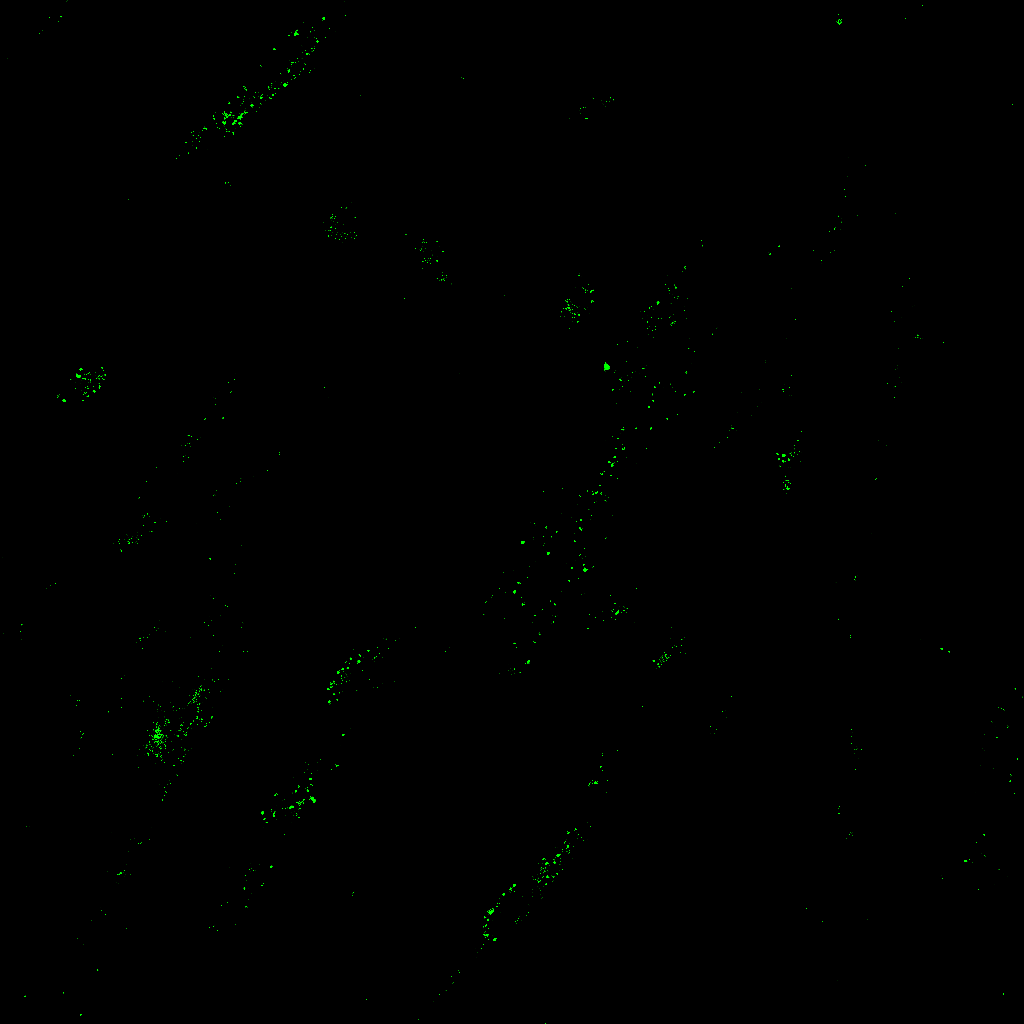

Supplement: Figure 3—source data 1. [file elife-75072-fig3-data1.zip › Figure 3-source data/figure3A/LPS+IL-33 siRNA(0h)/si merge 1c2.tif]

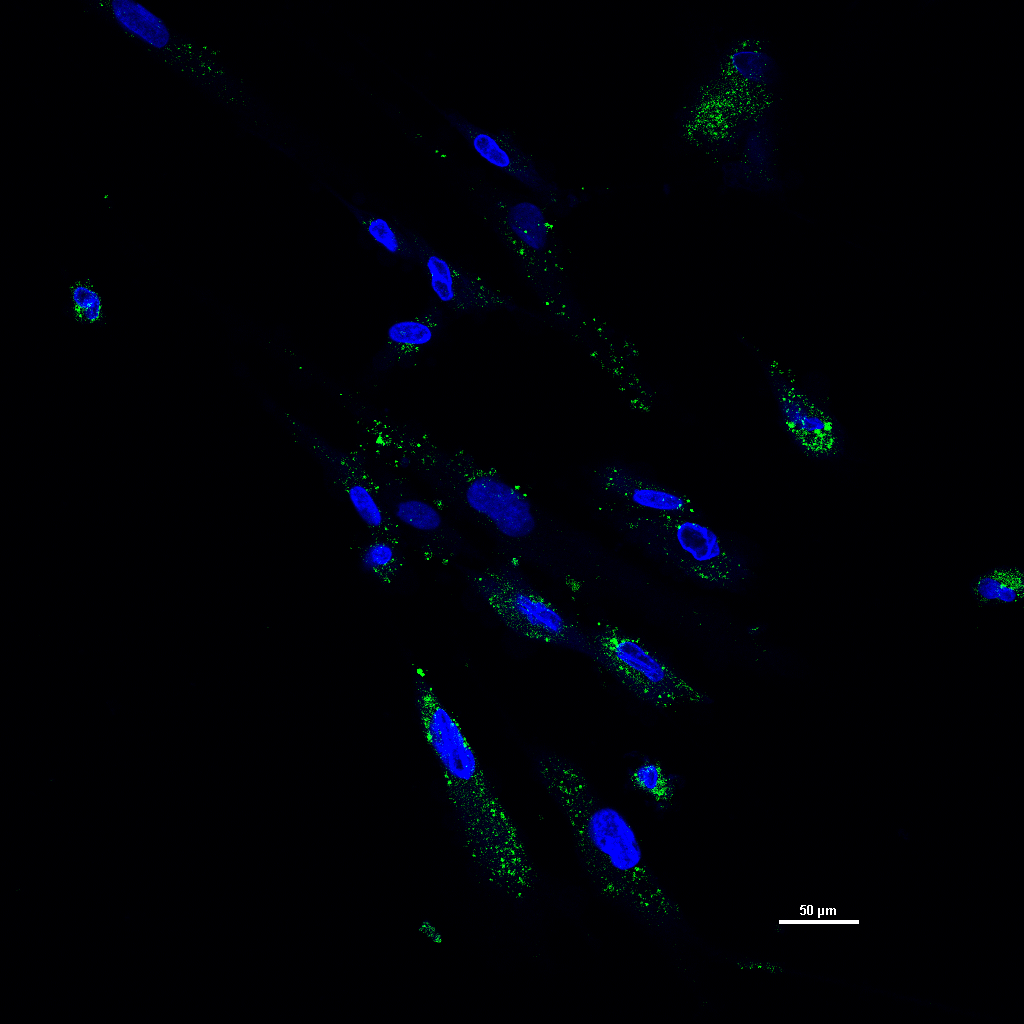

Supplement: Figure 3—source data 1. [file elife-75072-fig3-data1.zip › Figure 3-source data/figure3A/LPS+IL-33 siRNA(1h)/lps 1h+IL-33 siRNA-merge 1.tif]

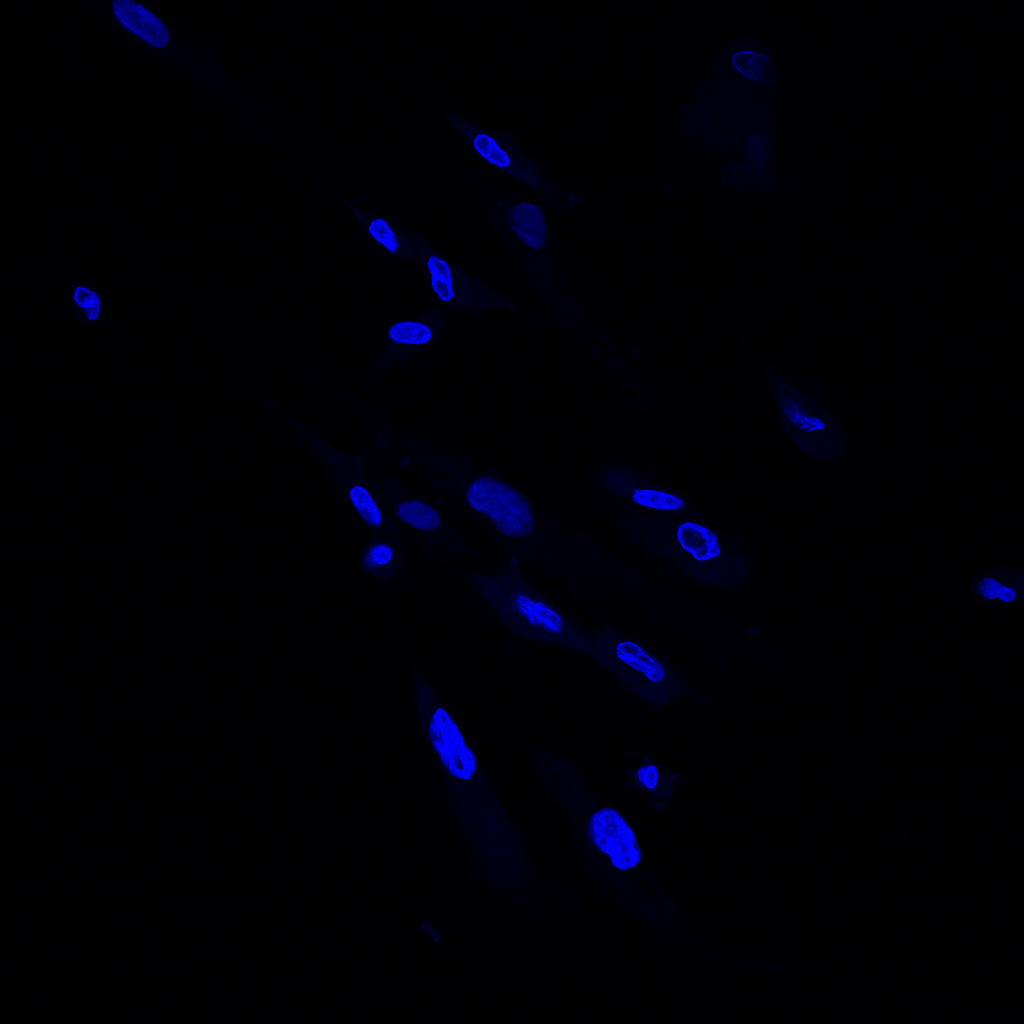

Supplement: Figure 3—source data 1. [file elife-75072-fig3-data1.zip › Figure 3-source data/figure3A/LPS+IL-33 siRNA(1h)/lps 1h+IL-33 siRNA-merge 1c1.tif]

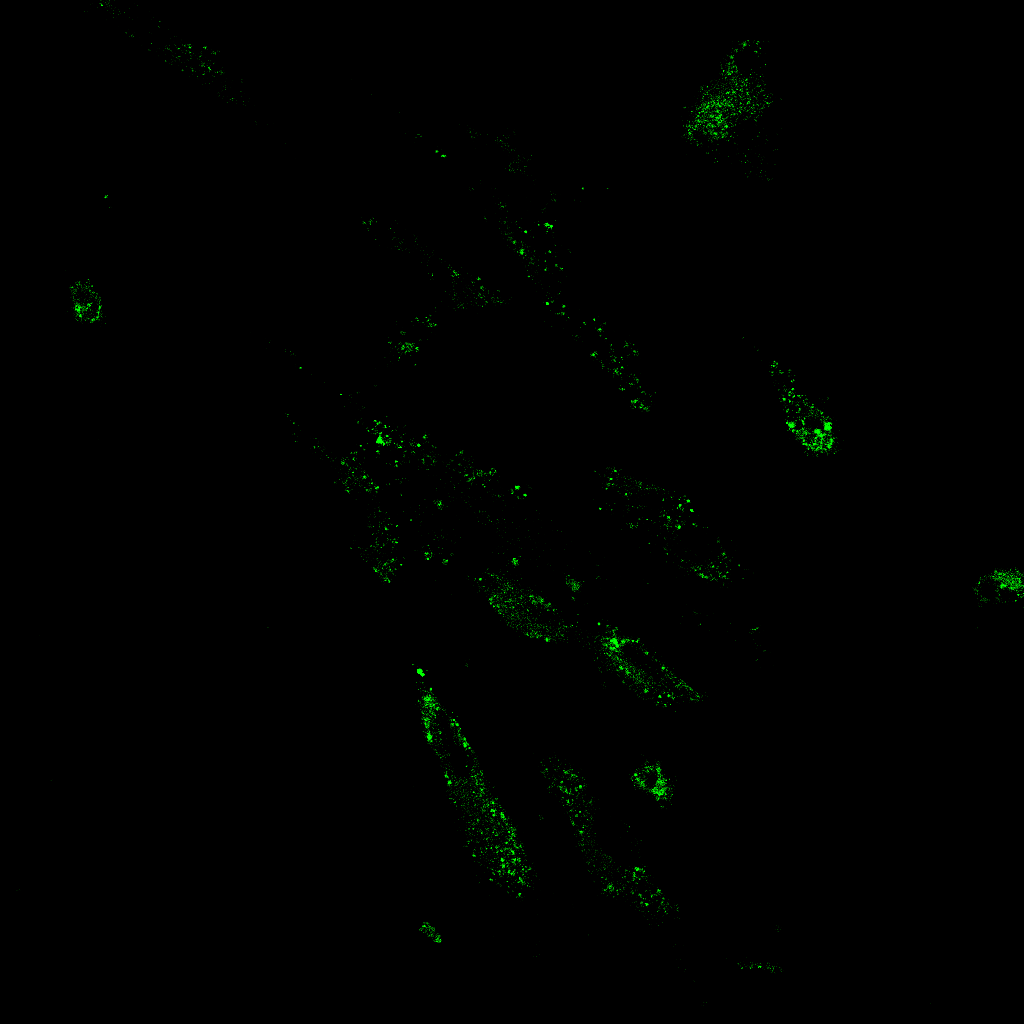

Supplement: Figure 3—source data 1. [file elife-75072-fig3-data1.zip › Figure 3-source data/figure3A/LPS+IL-33 siRNA(1h)/lps 1h+IL-33 siRNA-merge 1c2.tif]

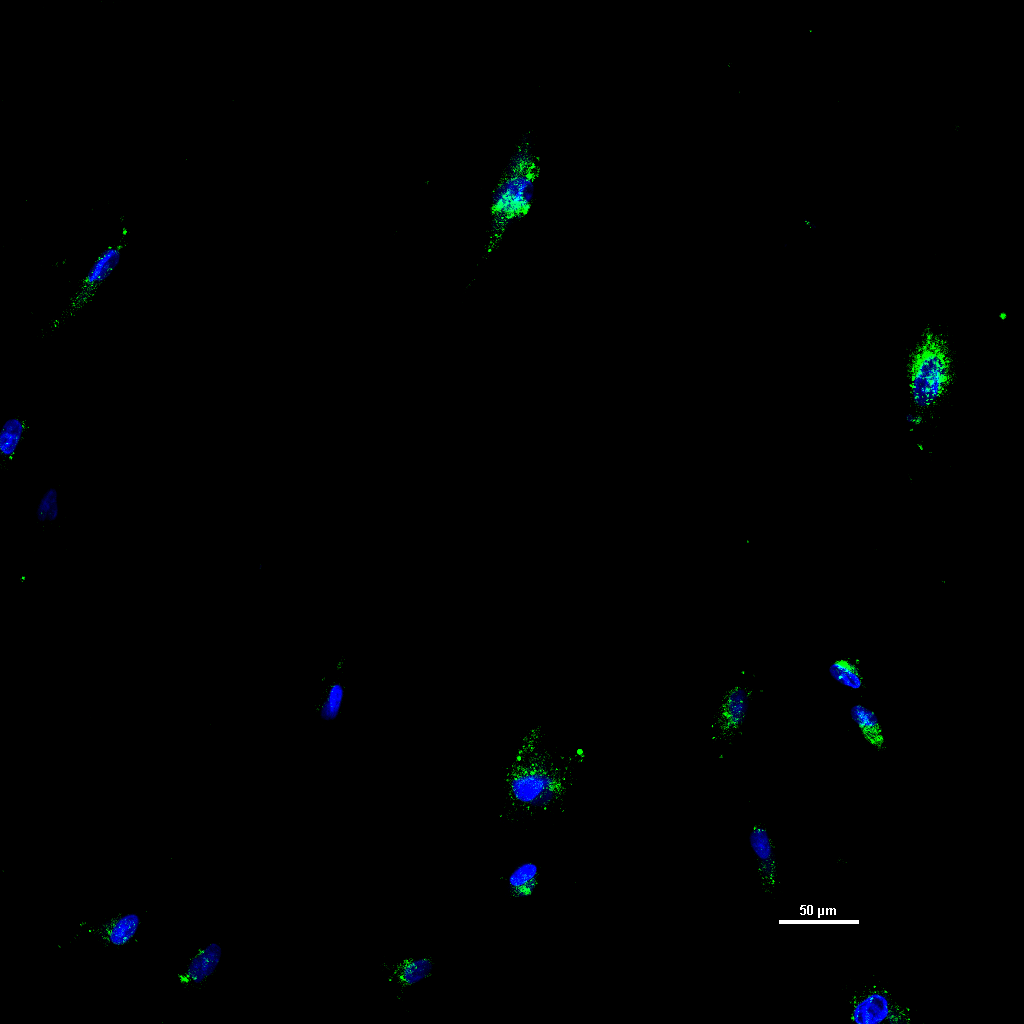

Supplement: Figure 3—source data 1. [file elife-75072-fig3-data1.zip › Figure 3-source data/figure3A/LPS+IL-33 siRNA(3h)/lps 3h+IL-33 siRNA-merge-1.tif]

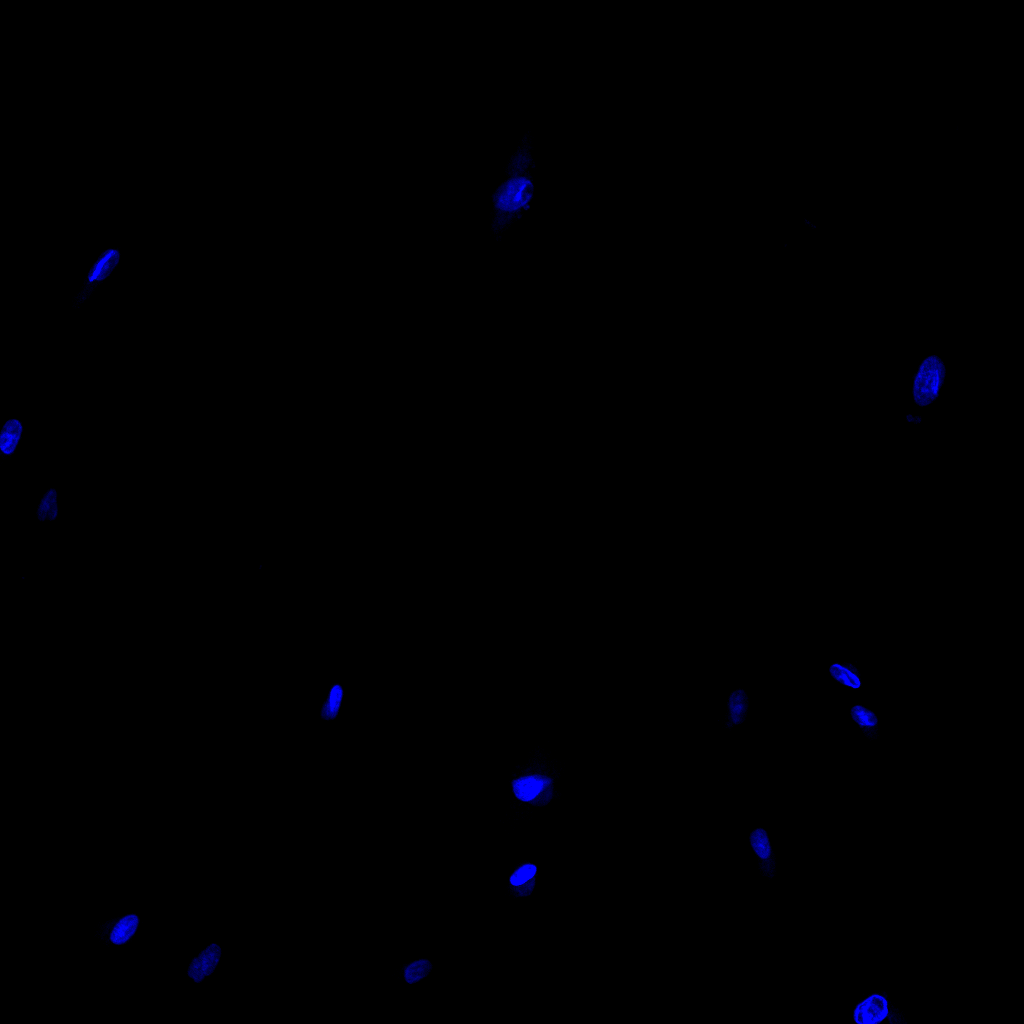

Supplement: Figure 3—source data 1. [file elife-75072-fig3-data1.zip › Figure 3-source data/figure3A/LPS+IL-33 siRNA(3h)/lps 3h+IL-33 siRNA-merge-1c1.tif]

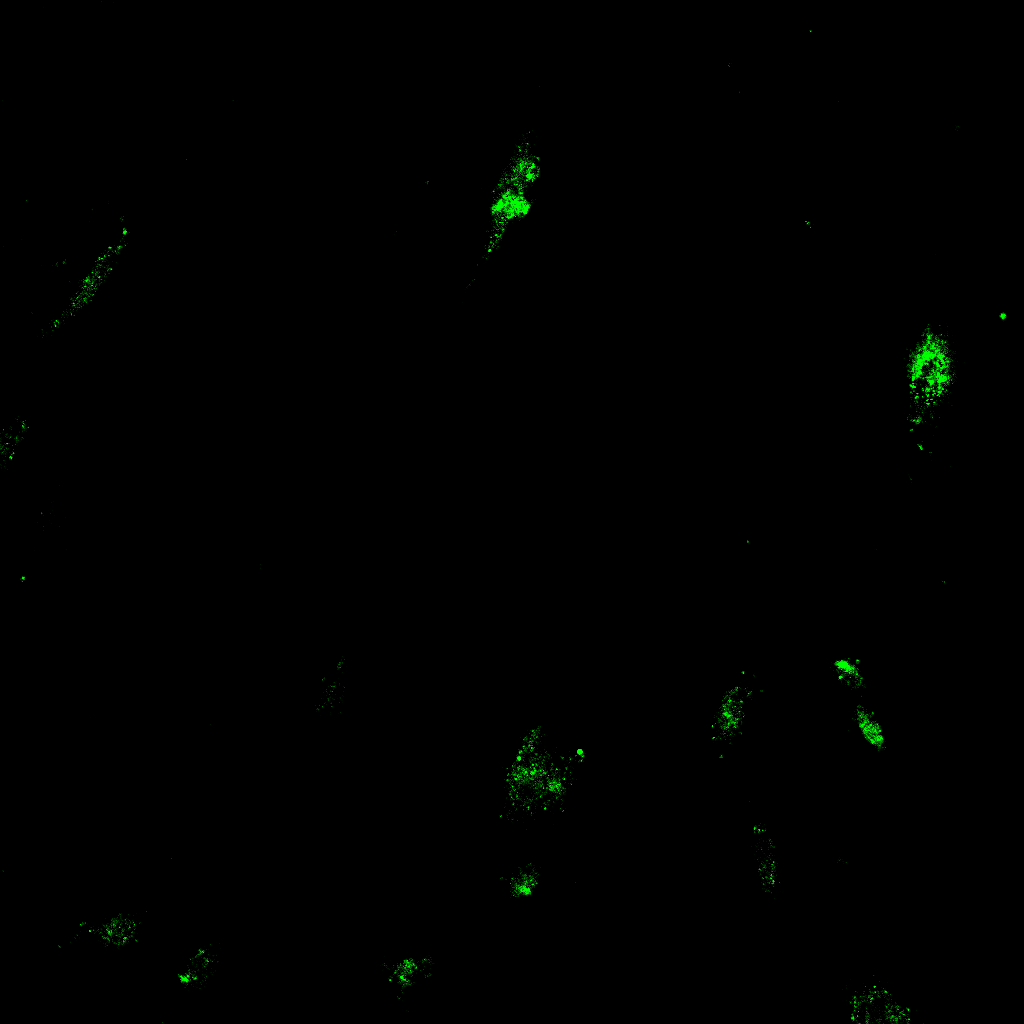

Supplement: Figure 3—source data 1. [file elife-75072-fig3-data1.zip › Figure 3-source data/figure3A/LPS+IL-33 siRNA(3h)/lps 3h+IL-33 siRNA-merge-1c2.tif]

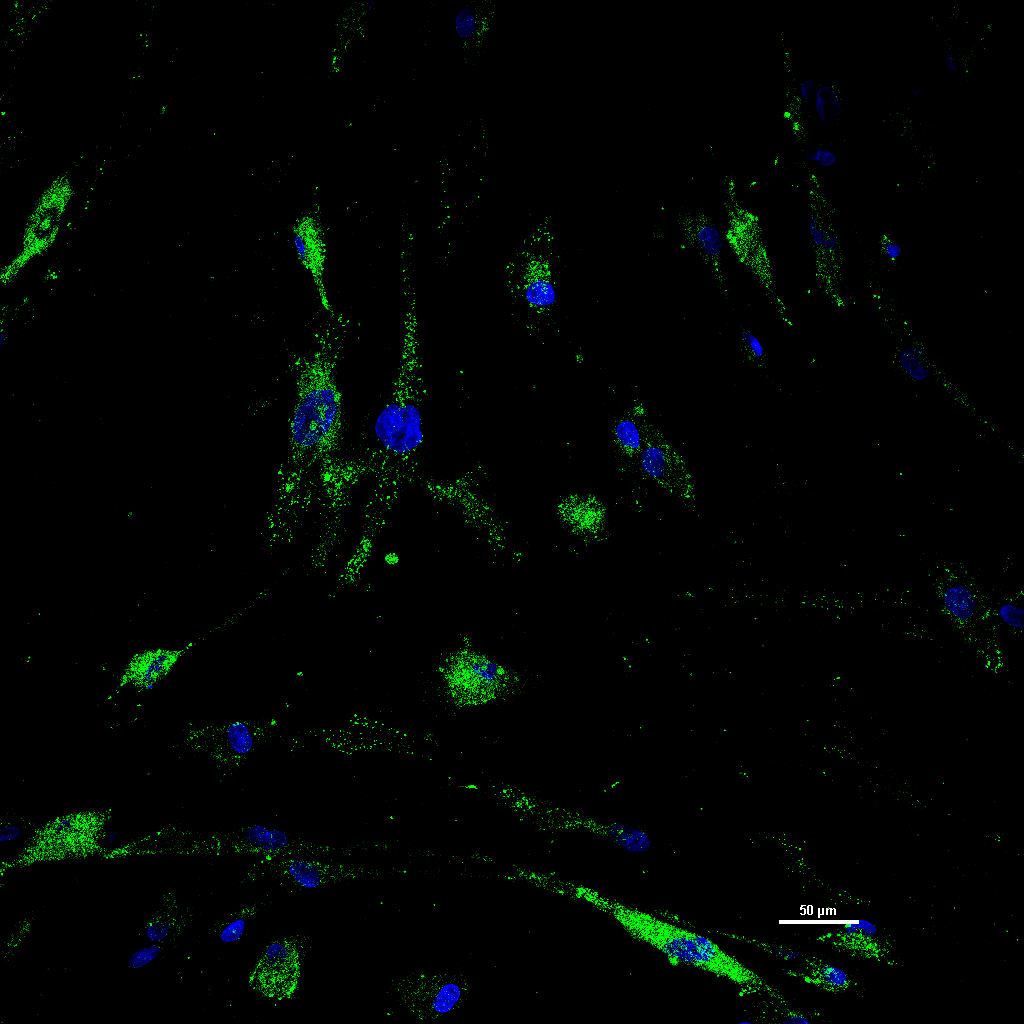

Supplement: Figure 3—source data 1. [file elife-75072-fig3-data1.zip › Figure 3-source data/figure3A/LPS+IL-33 siRNA(6h)/lps 6h+IL-33 siRNA-merge-1.tif]

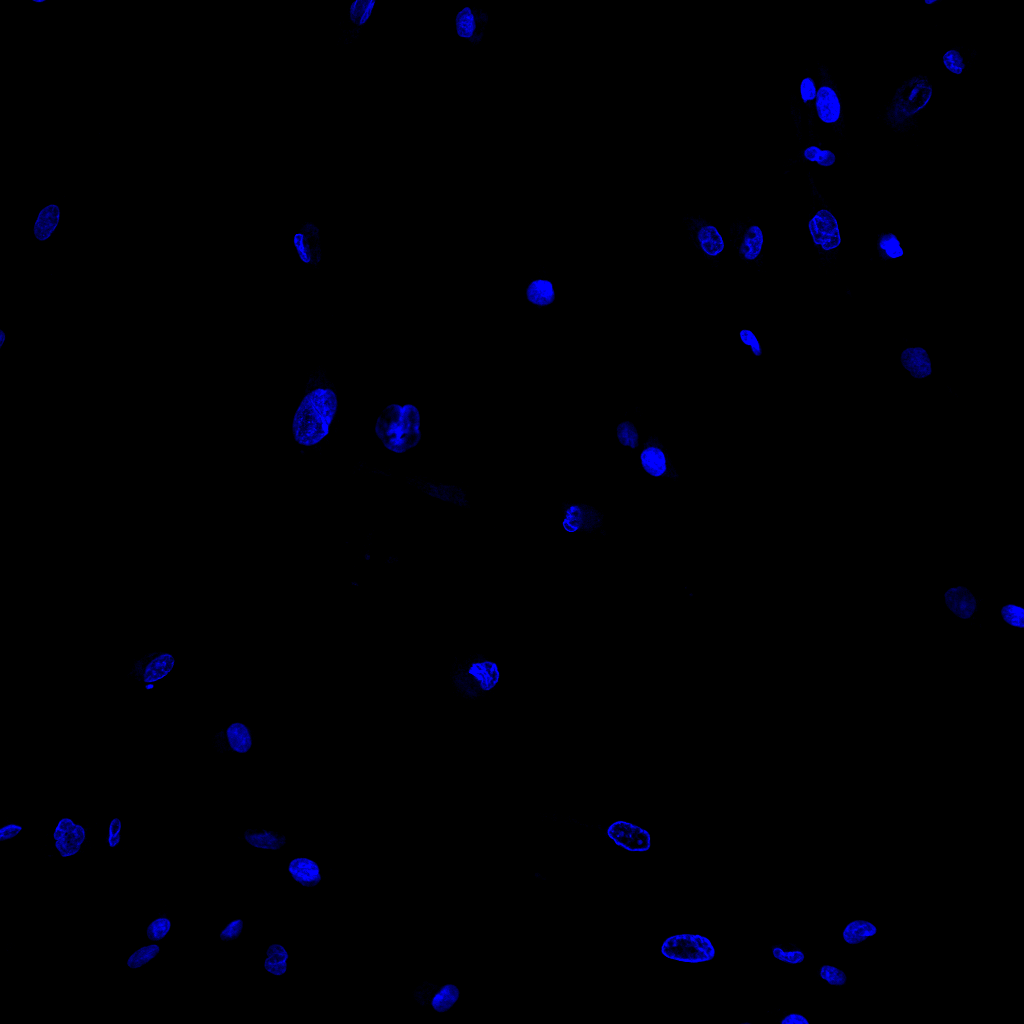

Supplement: Figure 3—source data 1. [file elife-75072-fig3-data1.zip › Figure 3-source data/figure3A/LPS+IL-33 siRNA(6h)/lps 6h+IL-33 siRNA-merge-1c1.tif]

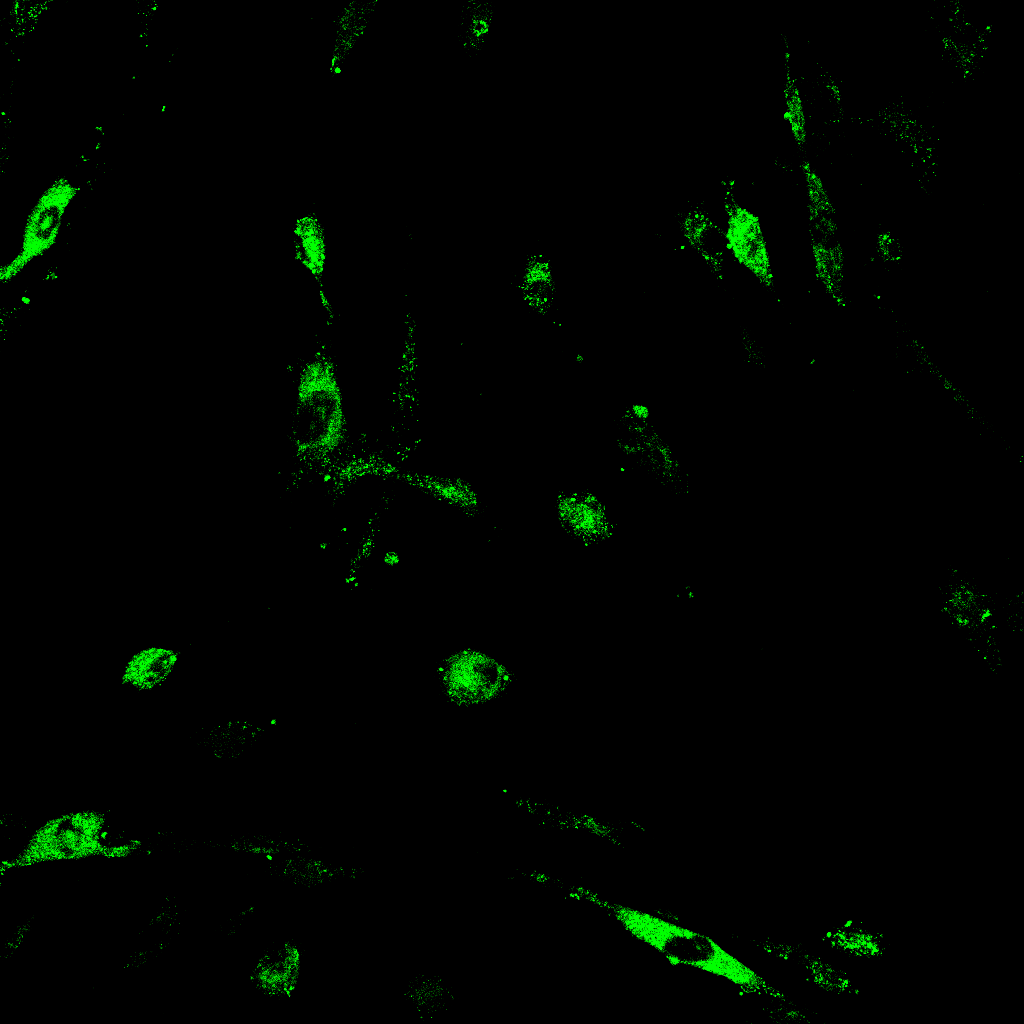

Supplement: Figure 3—source data 1. [file elife-75072-fig3-data1.zip › Figure 3-source data/figure3A/LPS+IL-33 siRNA(6h)/lps 6h+IL-33 siRNA-merge-1c2.tif]

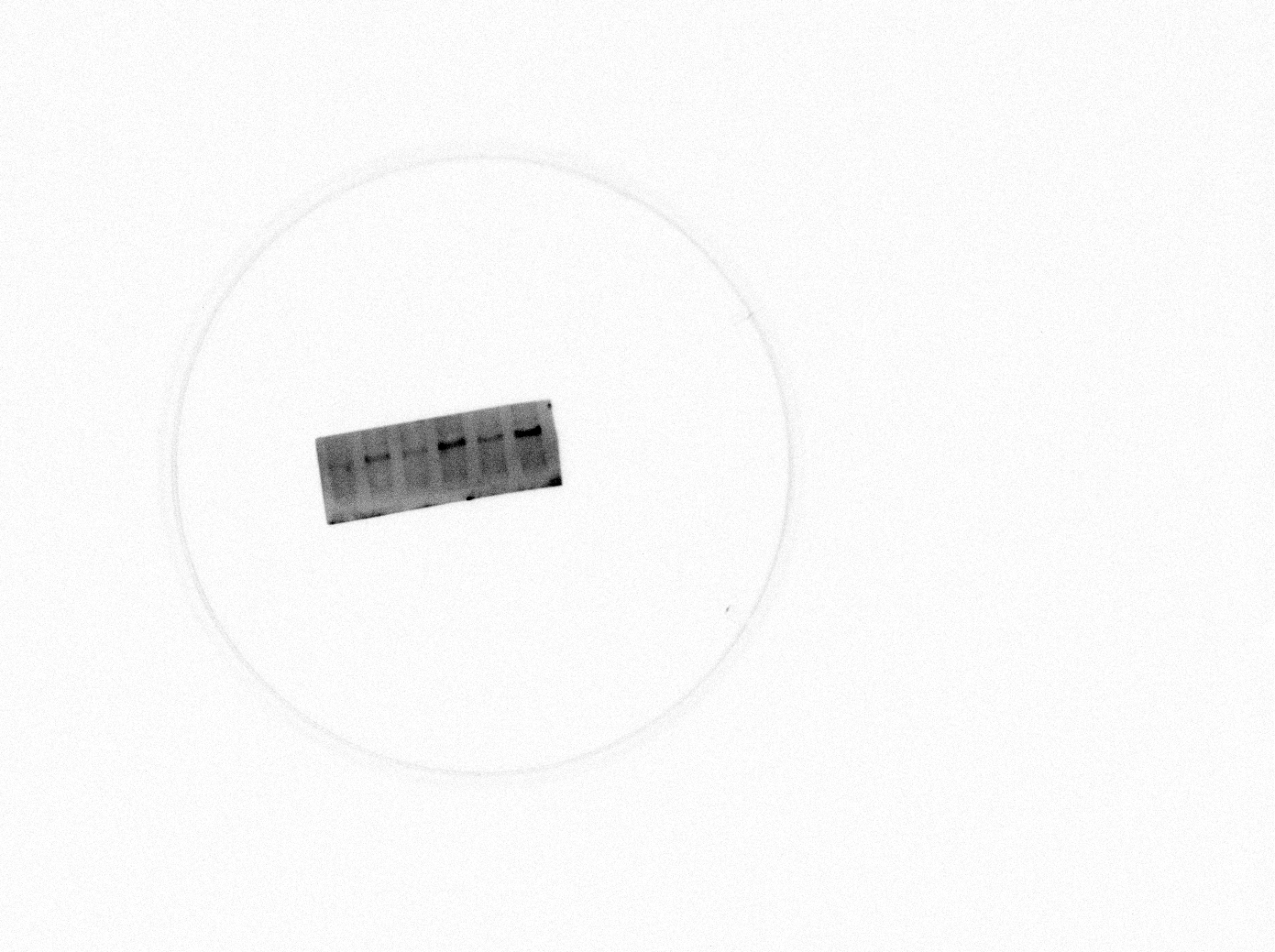

Supplement: Figure 3—source data 1. [file elife-75072-fig3-data1.zip › Figure 3-source data/Figure3B/Cav3.1.tif]

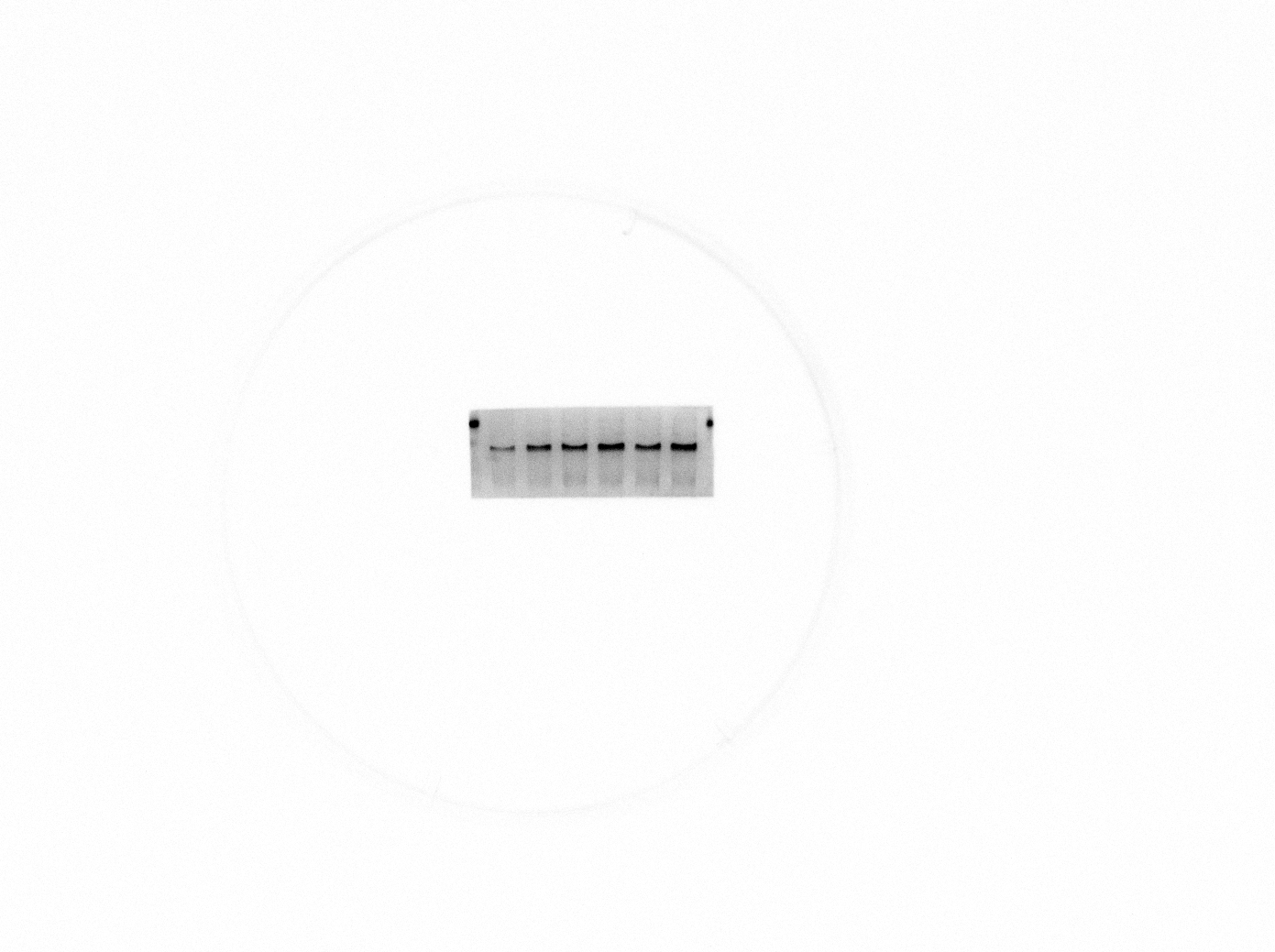

Supplement: Figure 3—source data 1. [file elife-75072-fig3-data1.zip › Figure 3-source data/Figure3B/Cav3.2.tif]

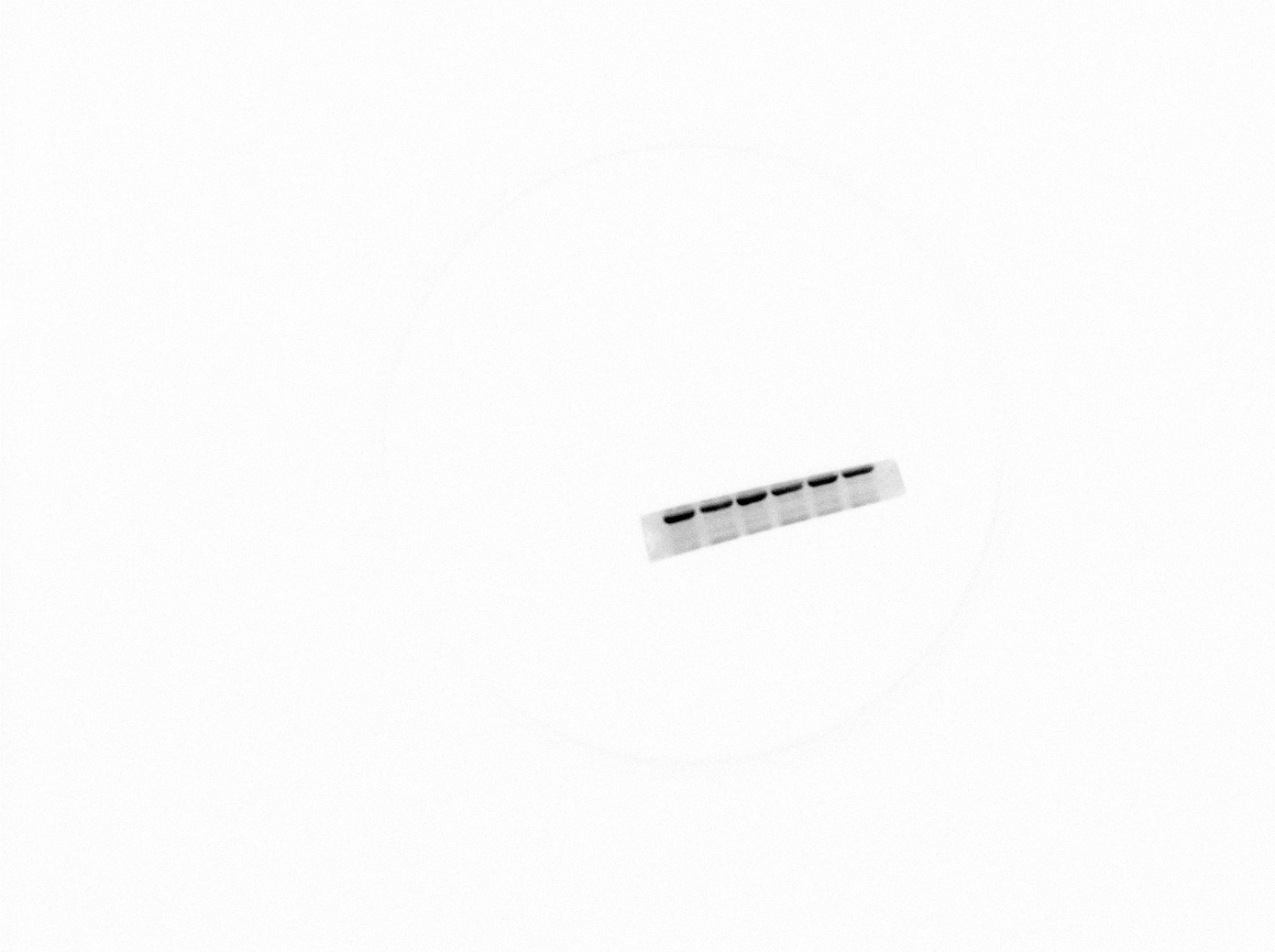

Supplement: Figure 3—source data 1. [file elife-75072-fig3-data1.zip › Figure 3-source data/Figure3B/β-actin.tif]

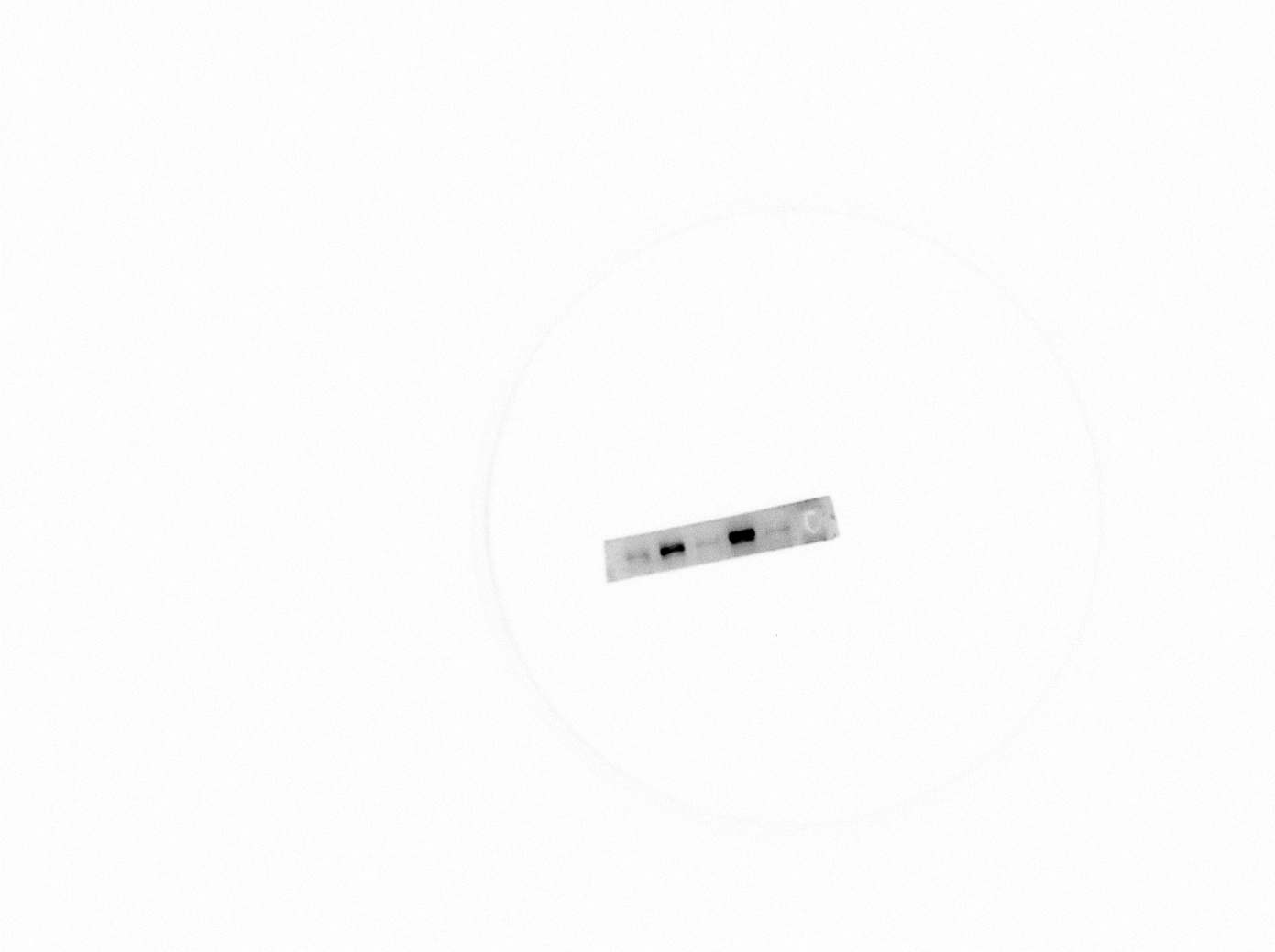

Supplement: Figure 4—source data 1. [file elife-75072-fig4-data1.zip › Figure 4-source data/Figure4A/COX-2.tif]

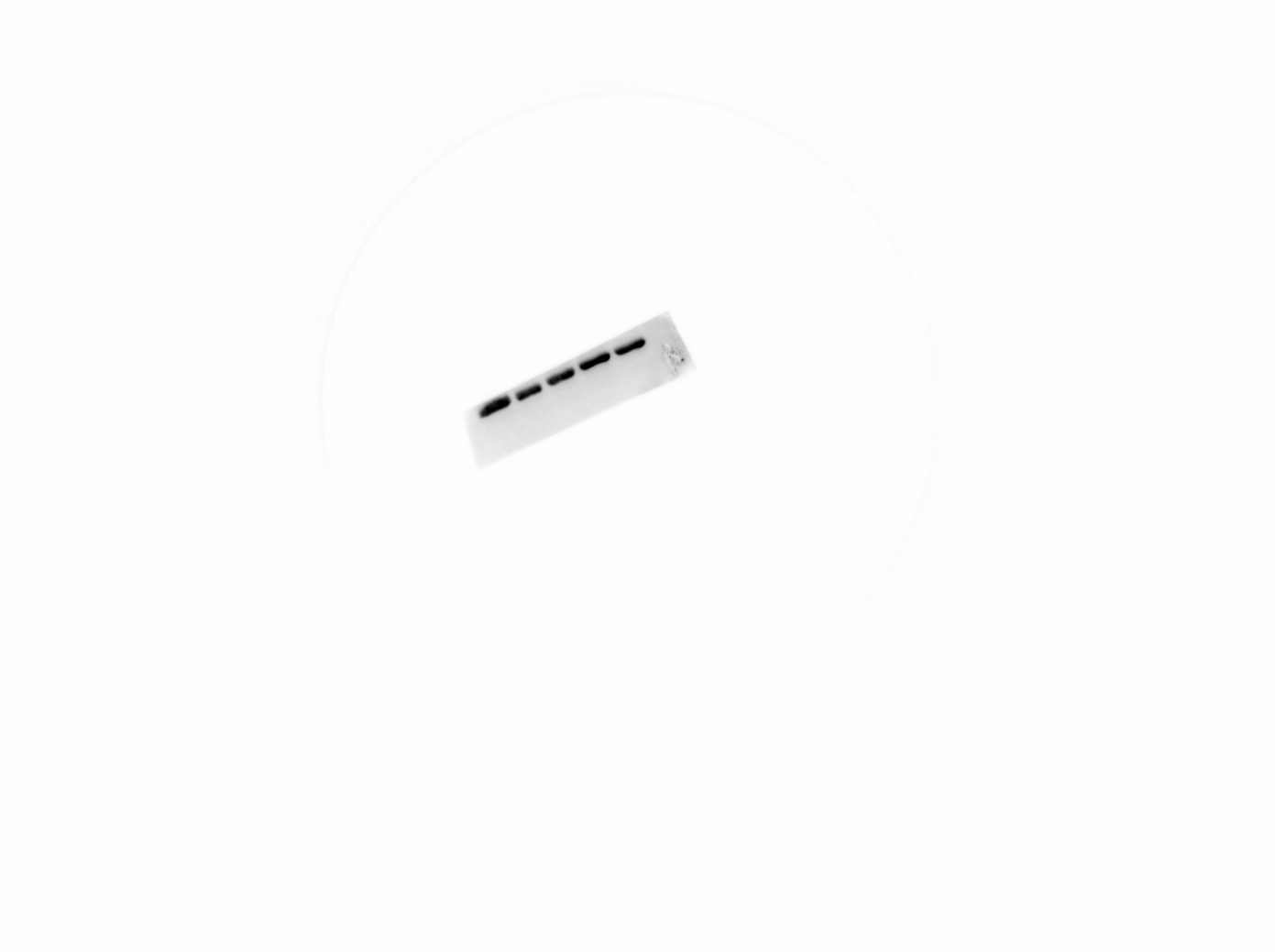

Supplement: Figure 4—source data 1. [file elife-75072-fig4-data1.zip › Figure 4-source data/Figure4A/β-actin.tif]

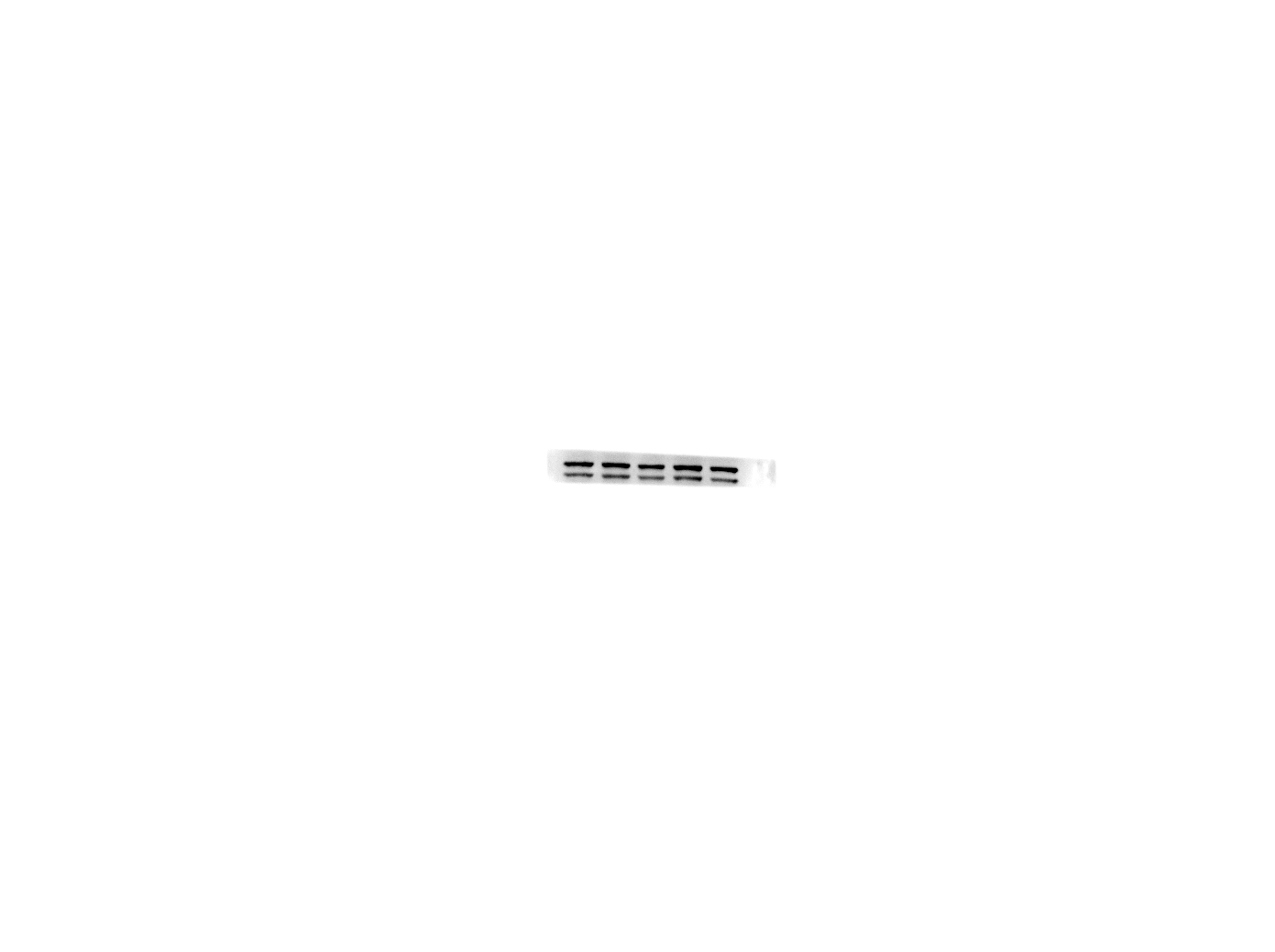

Supplement: Figure 4—source data 1. [file elife-75072-fig4-data1.zip › Figure 4-source data/Figure4B/GRP78.tif]

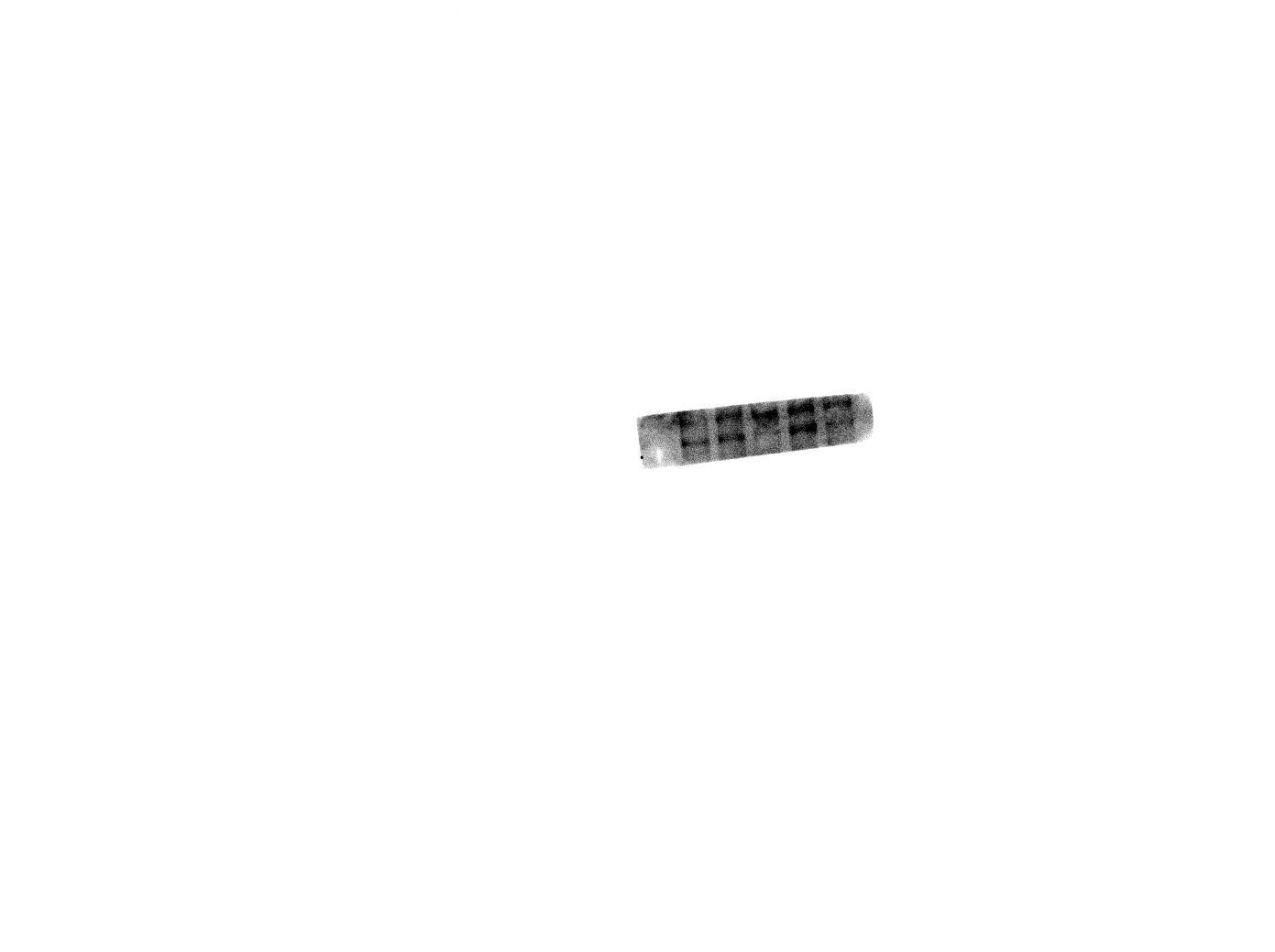

Supplement: Figure 4—source data 1. [file elife-75072-fig4-data1.zip › Figure 4-source data/Figure4B/p-IRE1α.tif]

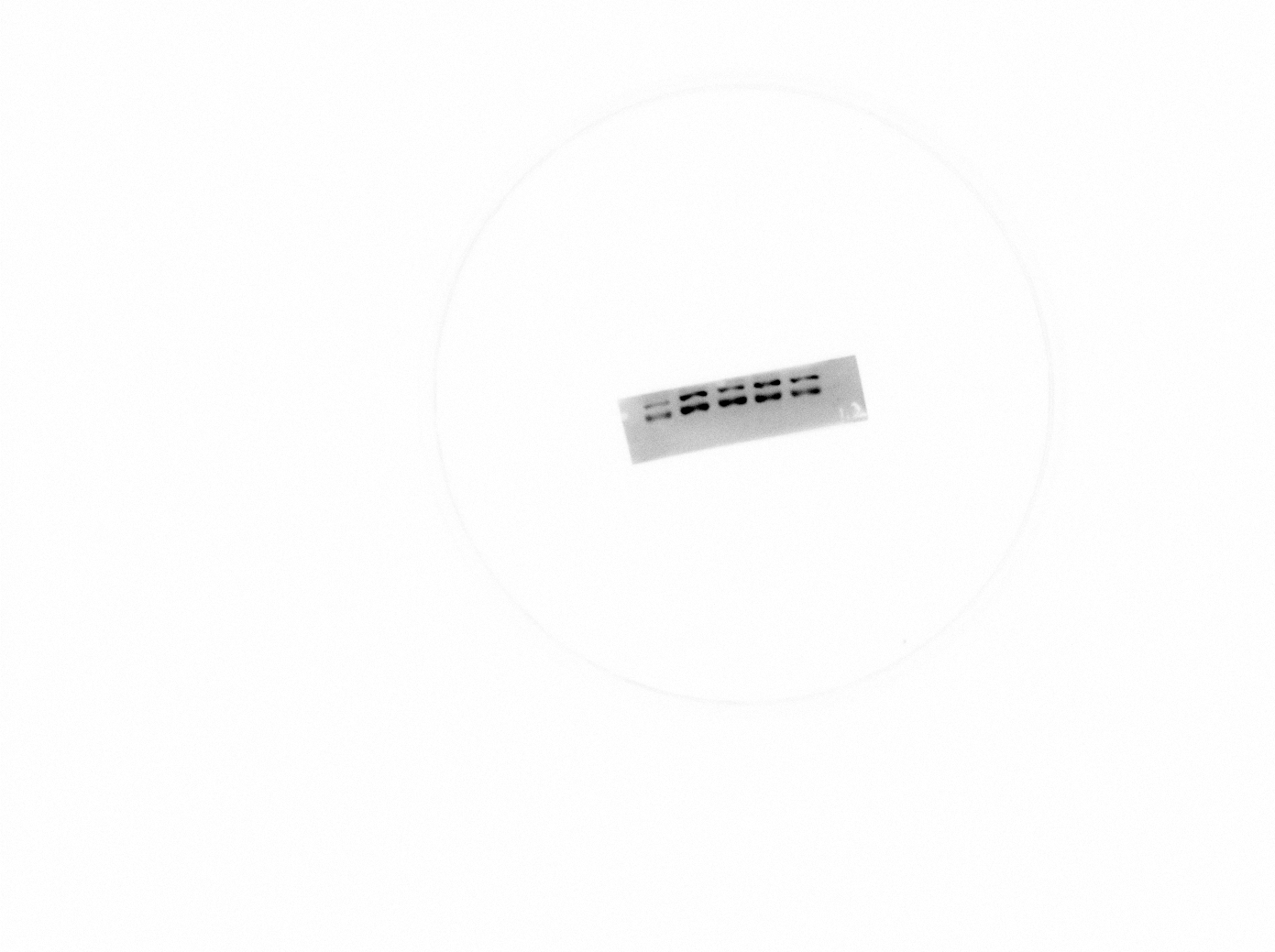

Supplement: Figure 4—source data 1. [file elife-75072-fig4-data1.zip › Figure 4-source data/Figure4B/XBP1s.tif]

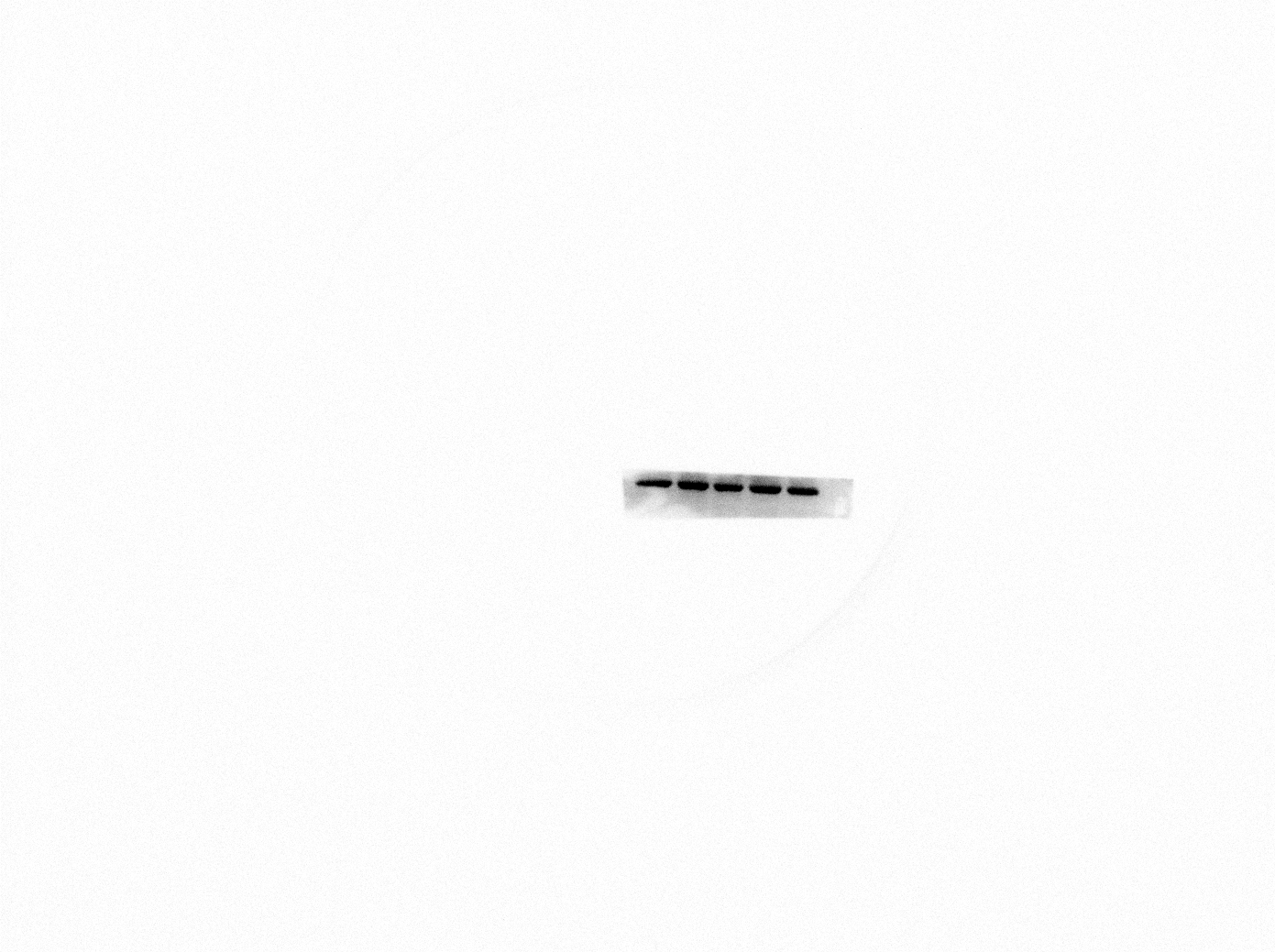

Supplement: Figure 4—source data 1. [file elife-75072-fig4-data1.zip › Figure 4-source data/Figure4B/β-actin.tif]

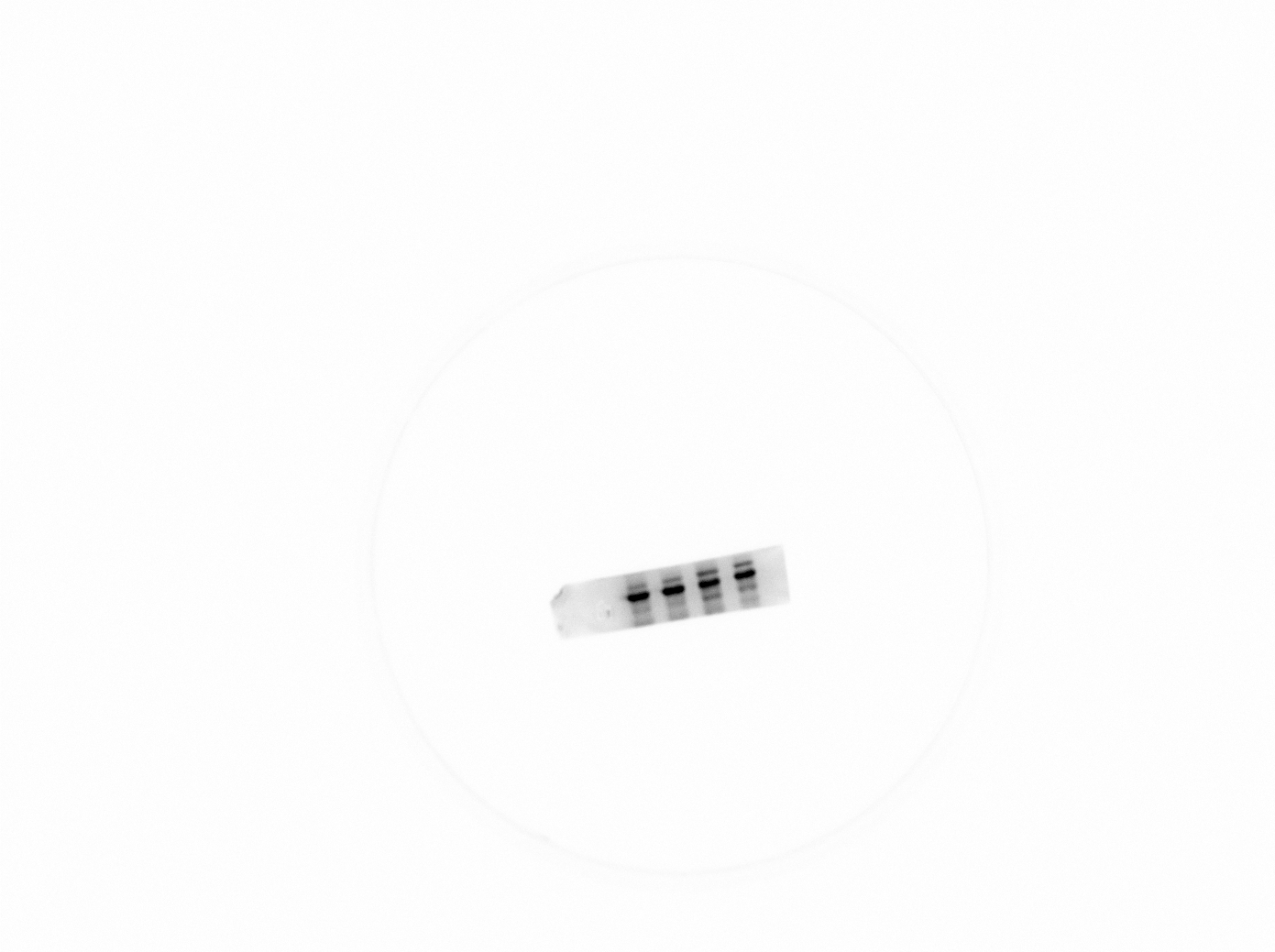

Supplement: Figure 5—source data 1. [file elife-75072-fig5-data1.zip › Figure 5-source data/figure5A/GAPDH.tif]

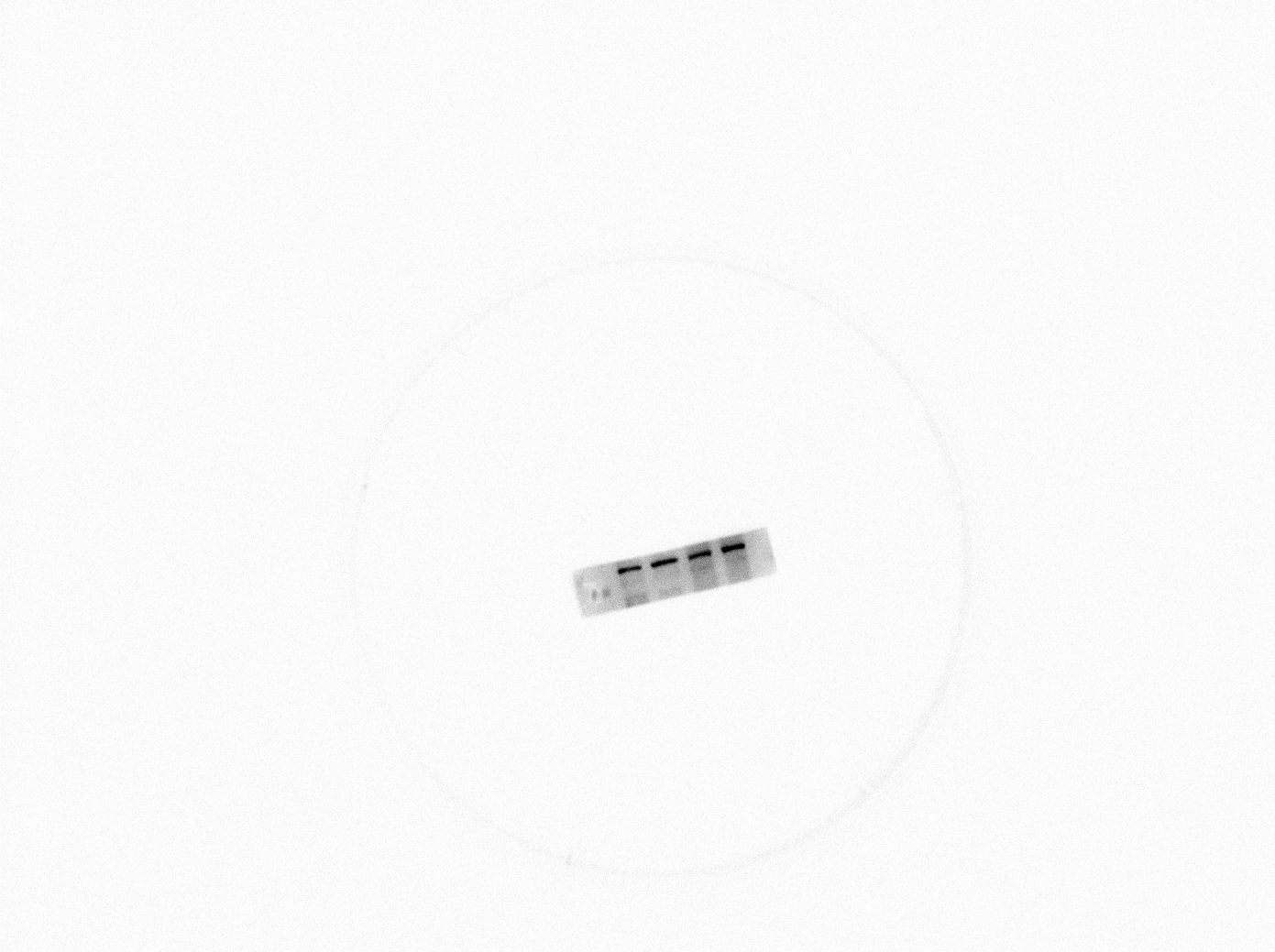

Supplement: Figure 5—source data 1. [file elife-75072-fig5-data1.zip › Figure 5-source data/figure5A/GRP78.tif]

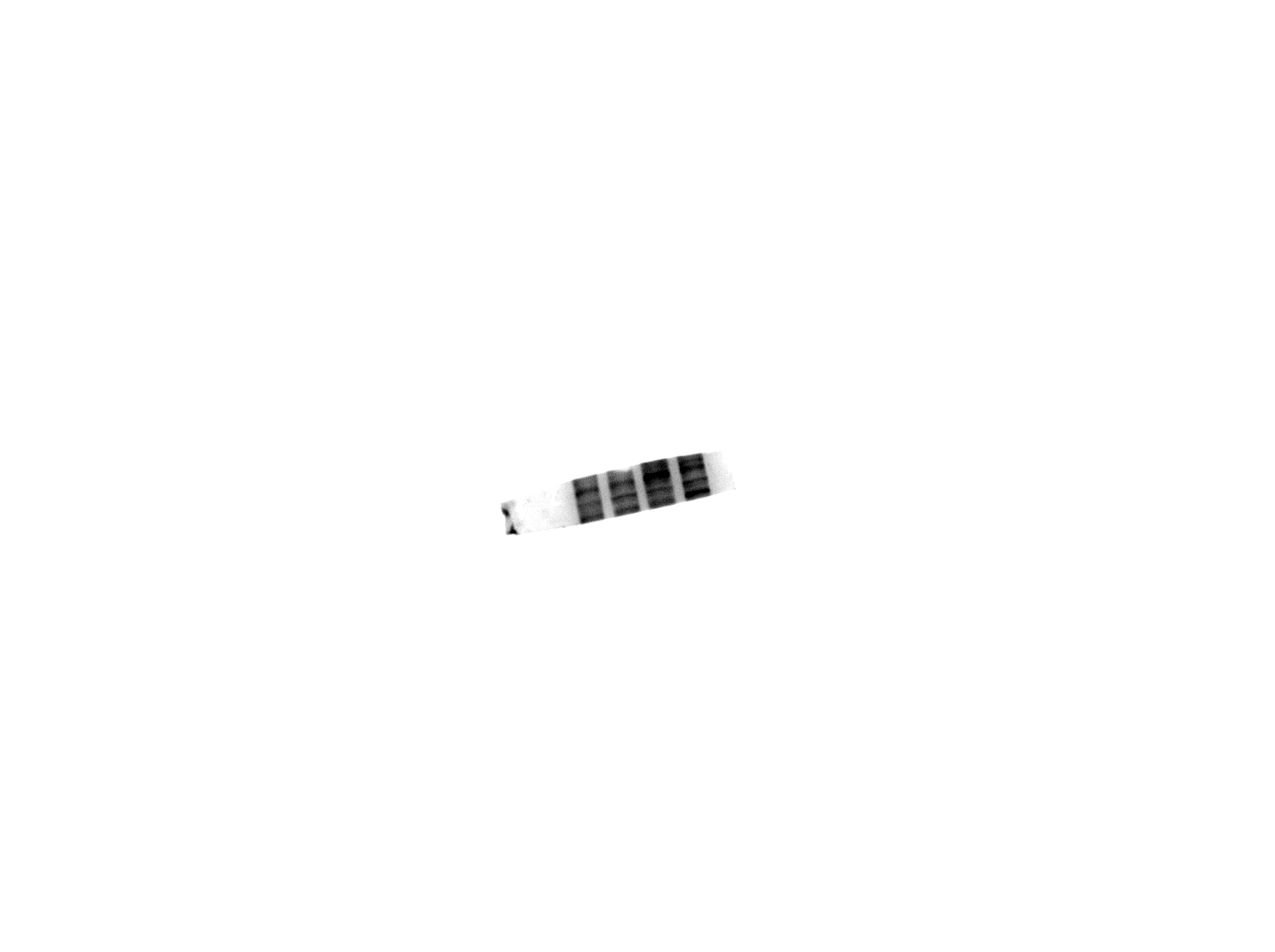

Supplement: Figure 5—source data 1. [file elife-75072-fig5-data1.zip › Figure 5-source data/figure5A/p-IRE1α.tif]

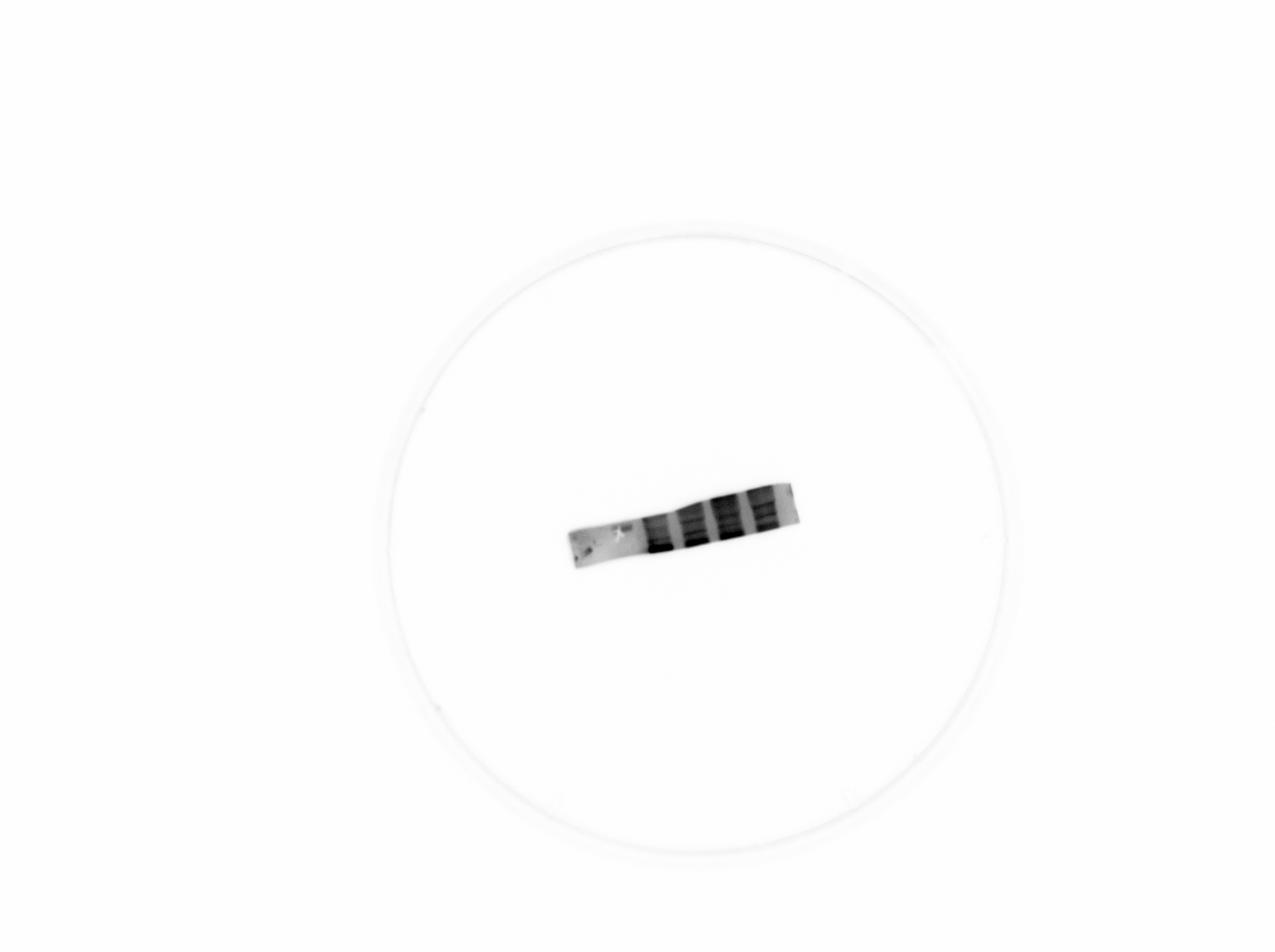

Supplement: Figure 5—source data 1. [file elife-75072-fig5-data1.zip › Figure 5-source data/figure5A/XBP1s.tif]

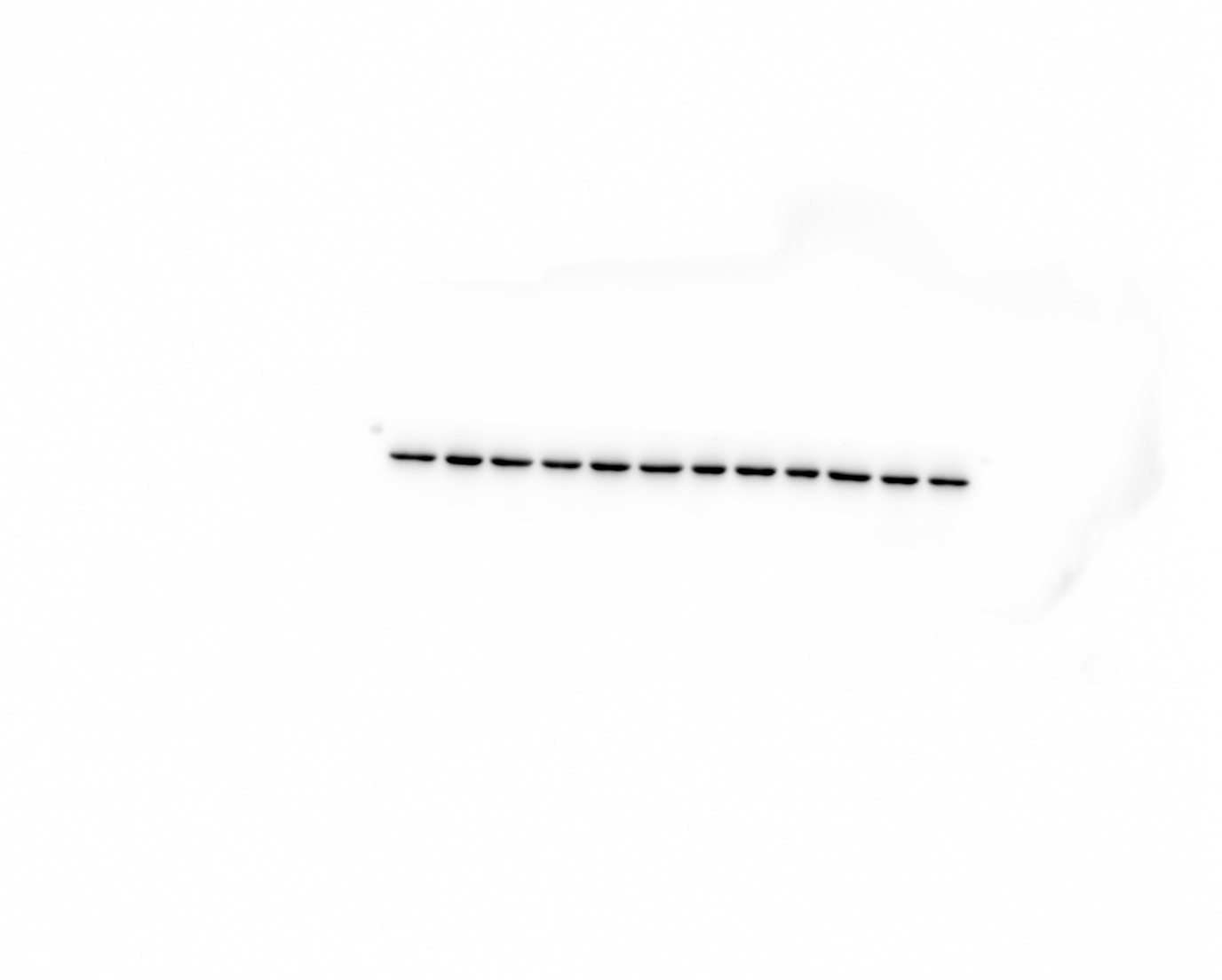

Supplement: Figure 5—source data 1. [file elife-75072-fig5-data1.zip › Figure 5-source data/figure5C/figure5c-repeated/1/GAPDH.Tif]

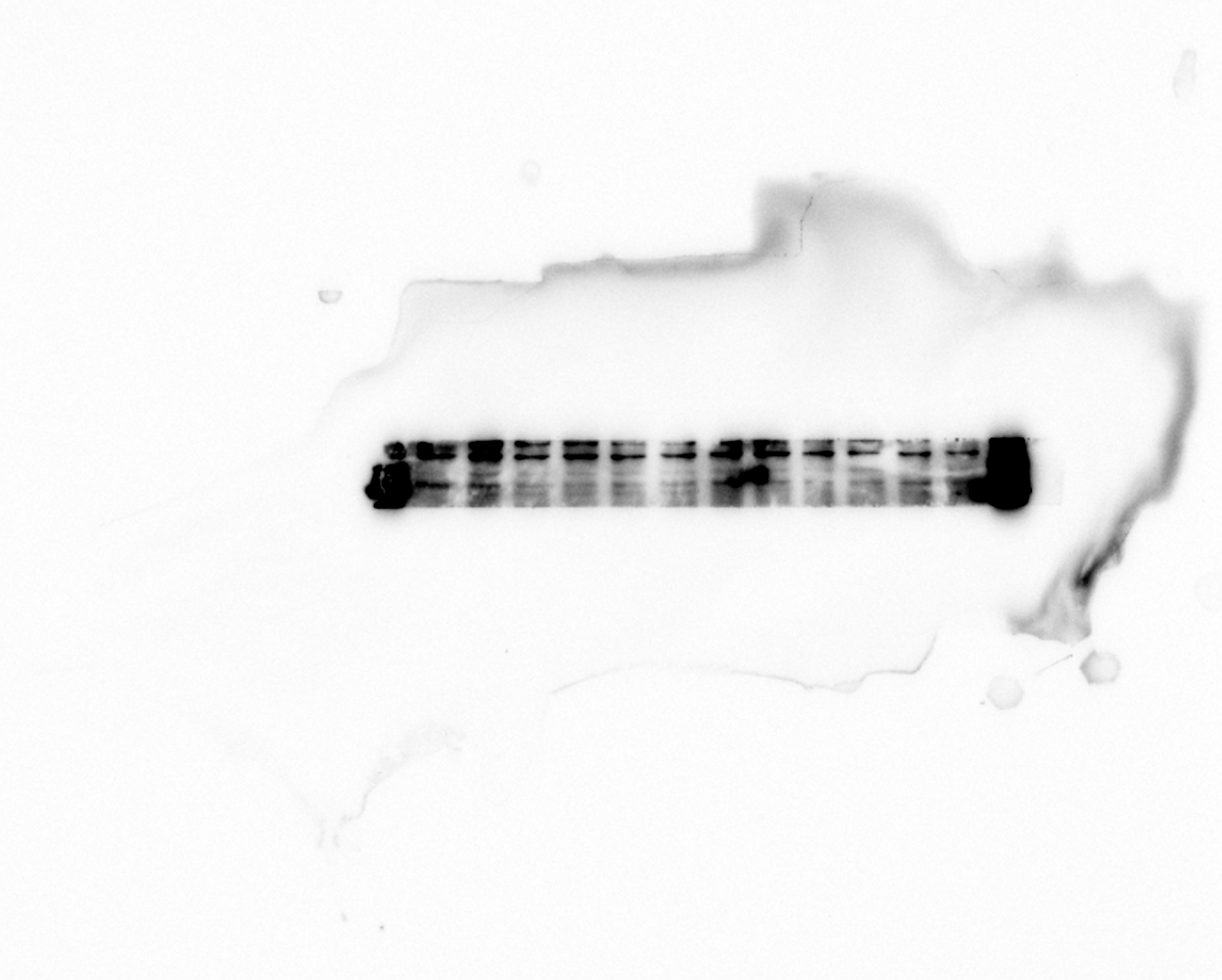

Supplement: Figure 5—source data 1. [file elife-75072-fig5-data1.zip › Figure 5-source data/figure5C/figure5c-repeated/1/p-IRE1α.Tif]

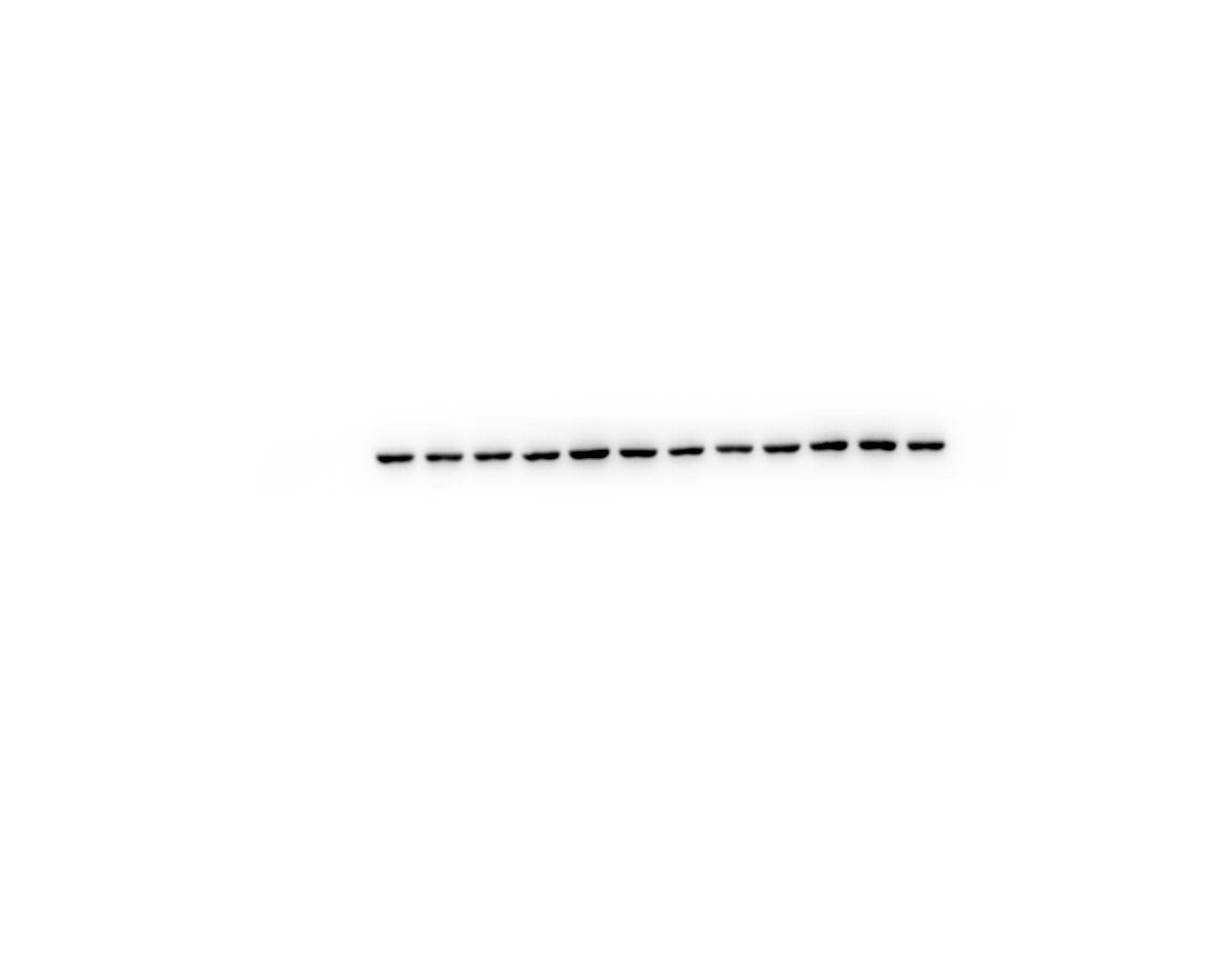

Supplement: Figure 5—source data 1. [file elife-75072-fig5-data1.zip › Figure 5-source data/figure5C/figure5c-repeated/2/GAPDH.Tif]

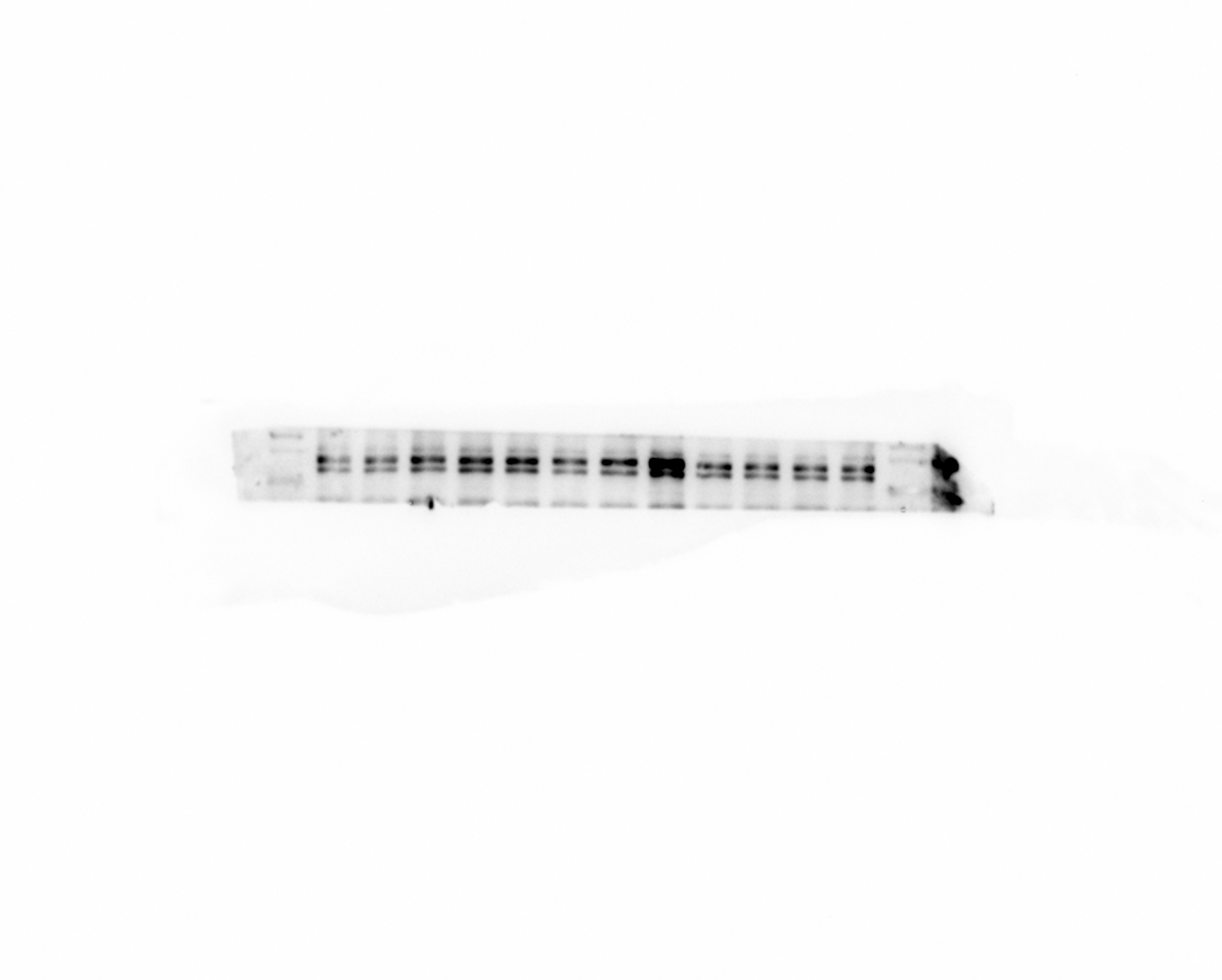

Supplement: Figure 5—source data 1. [file elife-75072-fig5-data1.zip › Figure 5-source data/figure5C/figure5c-repeated/2/p-IRE1α.Tif]

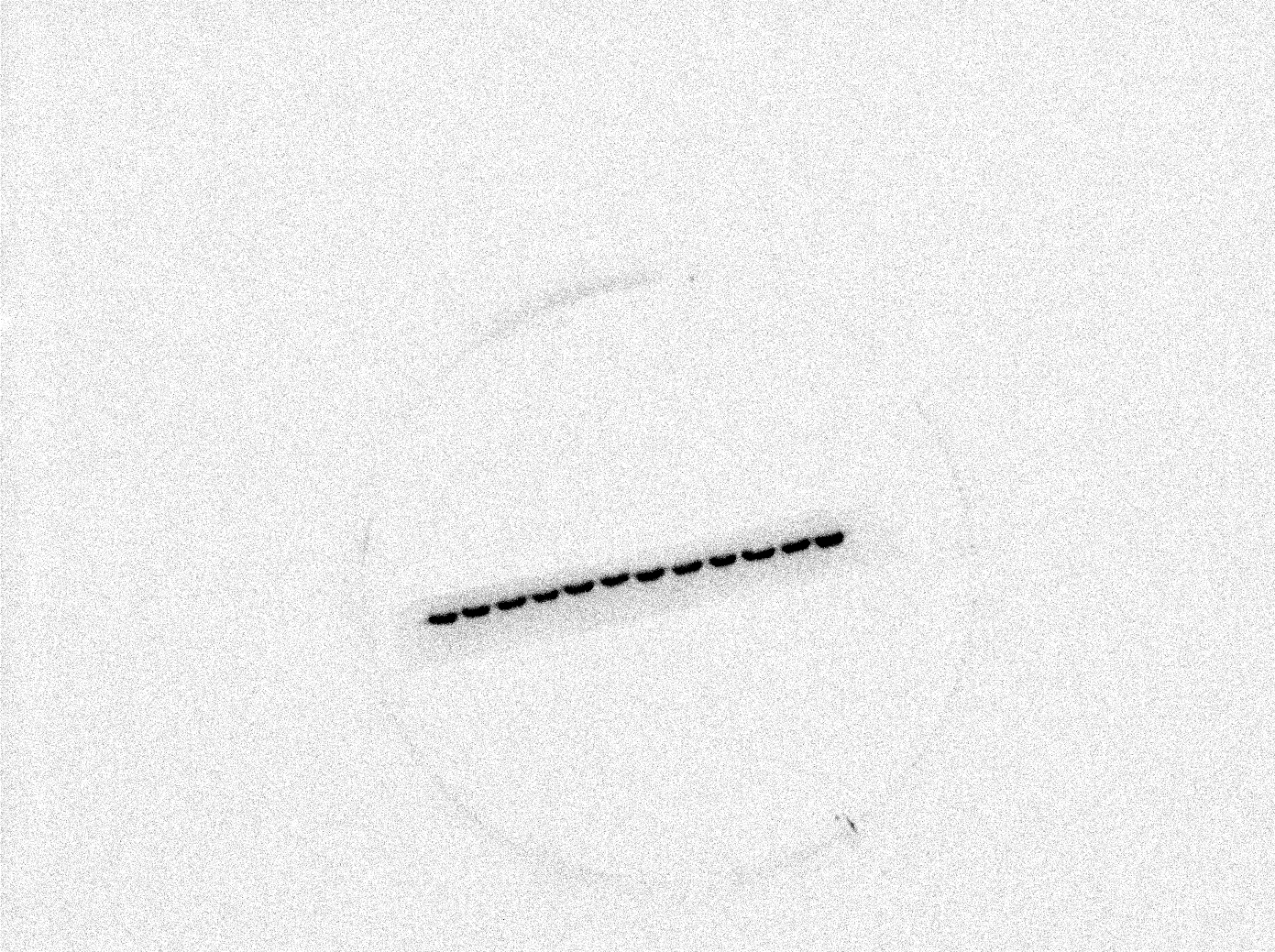

Supplement: Figure 5—source data 1. [file elife-75072-fig5-data1.zip › Figure 5-source data/figure5C/GAPDH.tif]

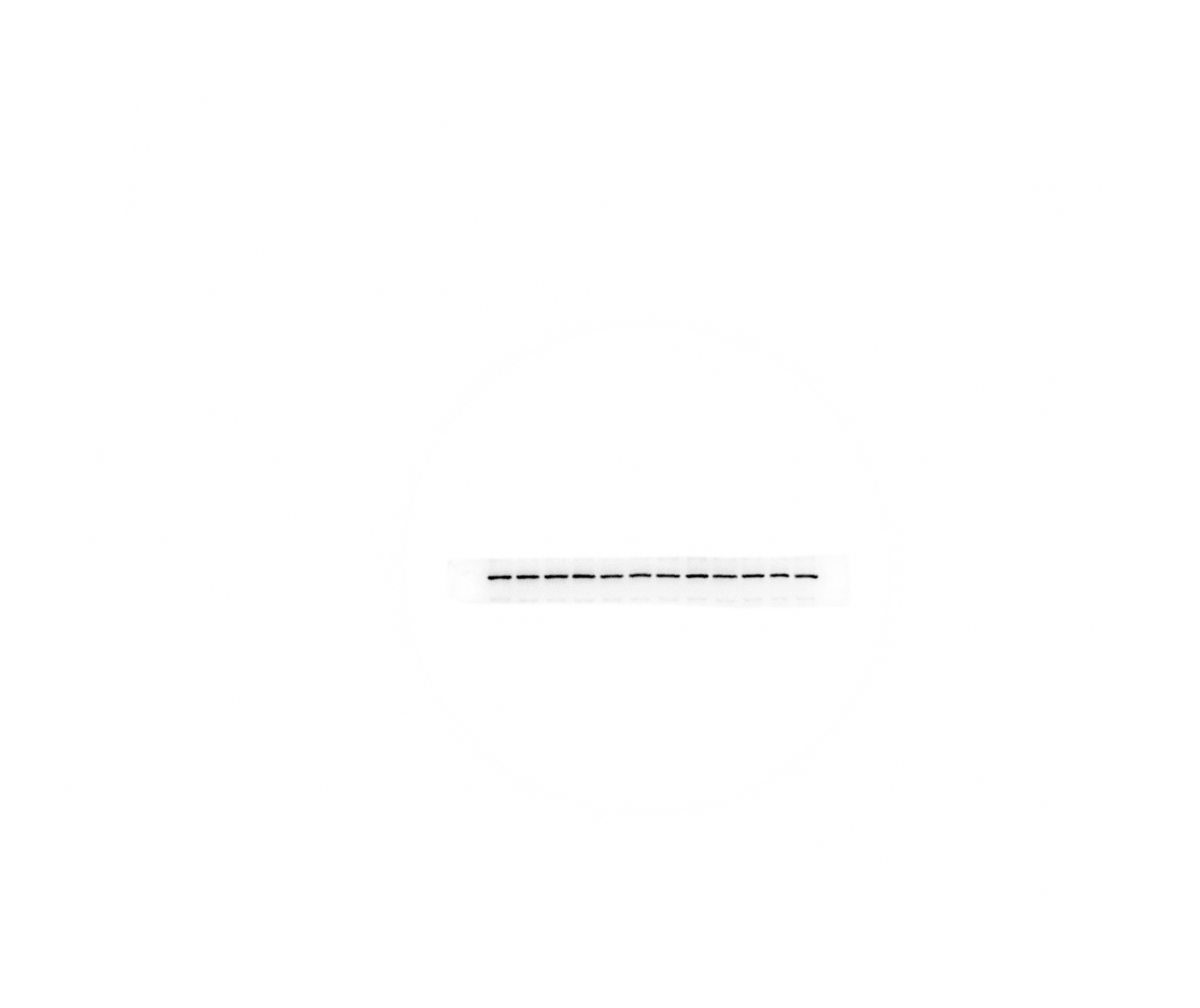

Supplement: Figure 5—source data 1. [file elife-75072-fig5-data1.zip › Figure 5-source data/figure5C/GRP78.png]

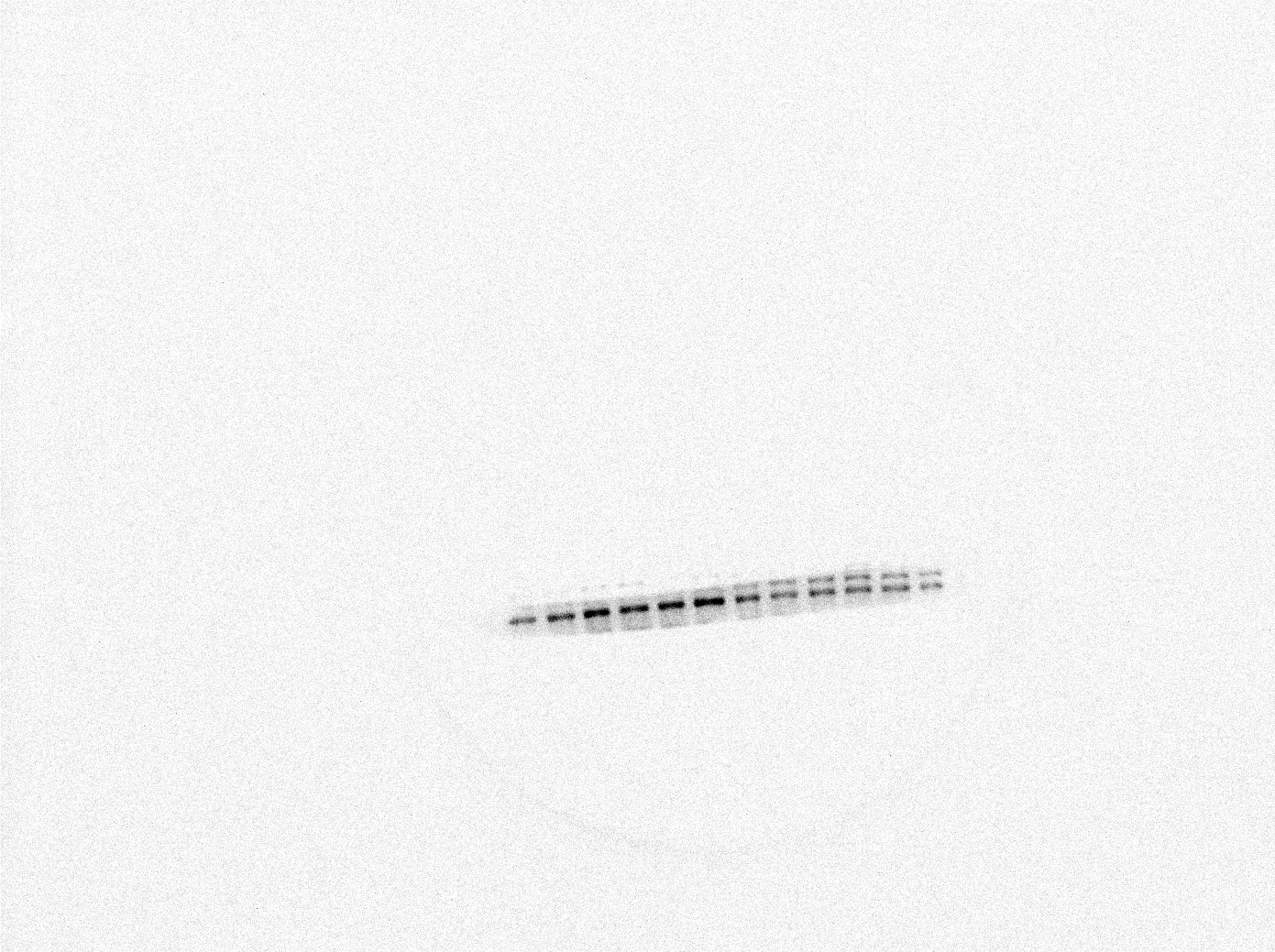

Supplement: Figure 5—source data 1. [file elife-75072-fig5-data1.zip › Figure 5-source data/figure5C/p-IRE1α.tif]
