# Supplementary figures and images for: Interleukin-33 regulates the endoplasmic reticulum stress of human myometrium via an influx of calcium during initiation of labor (part 2 of 2)
Source: eLife. 2022 Aug 23;11:e75072. doi: 10.7554/eLife.75072 (PMC9398448; doi:10.7554/eLife.75072)

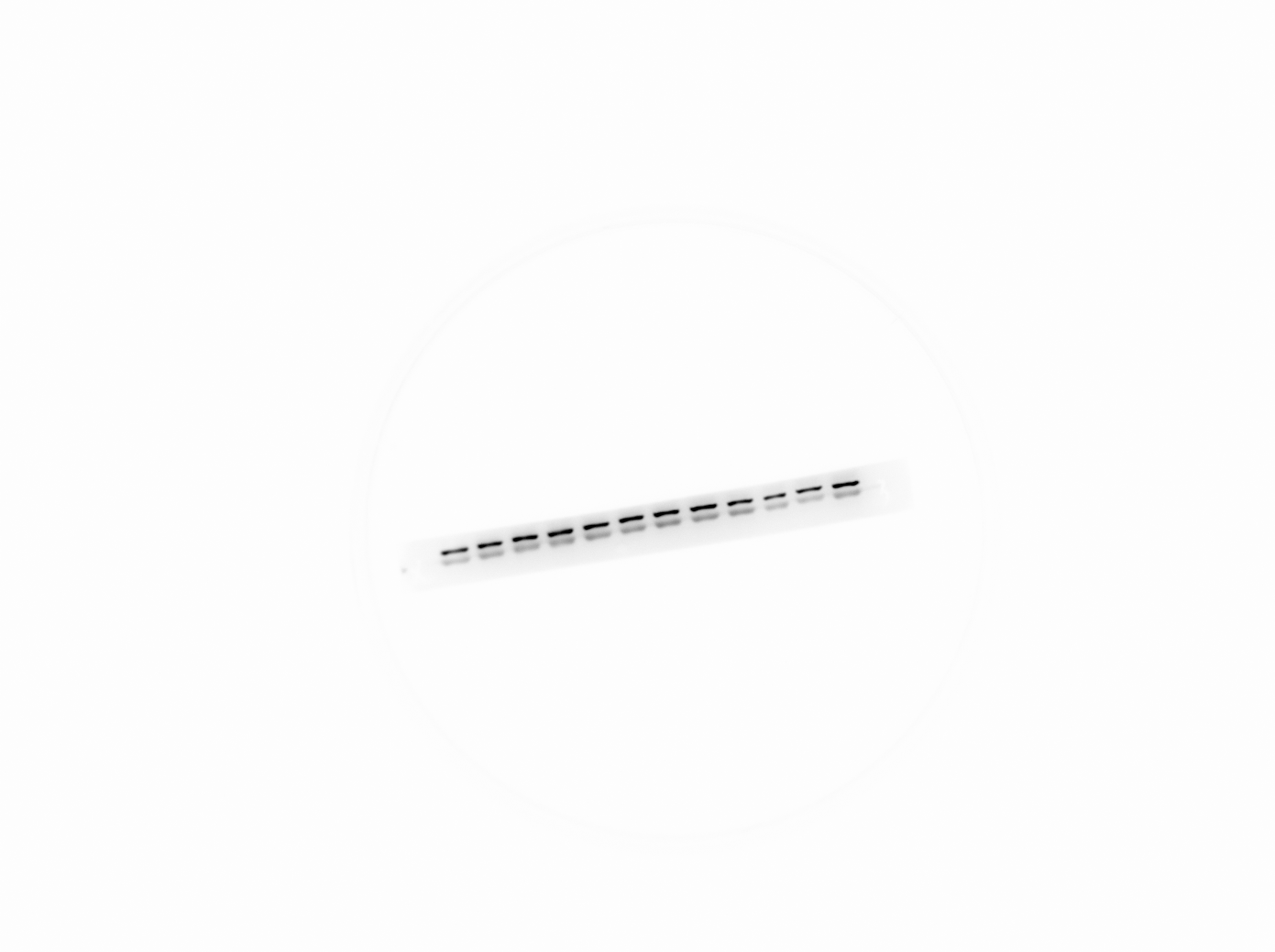

Supplement: Figure 5—source data 1. [file elife-75072-fig5-data1.zip › Figure 5-source data/figure5C/XBP1s.tif]

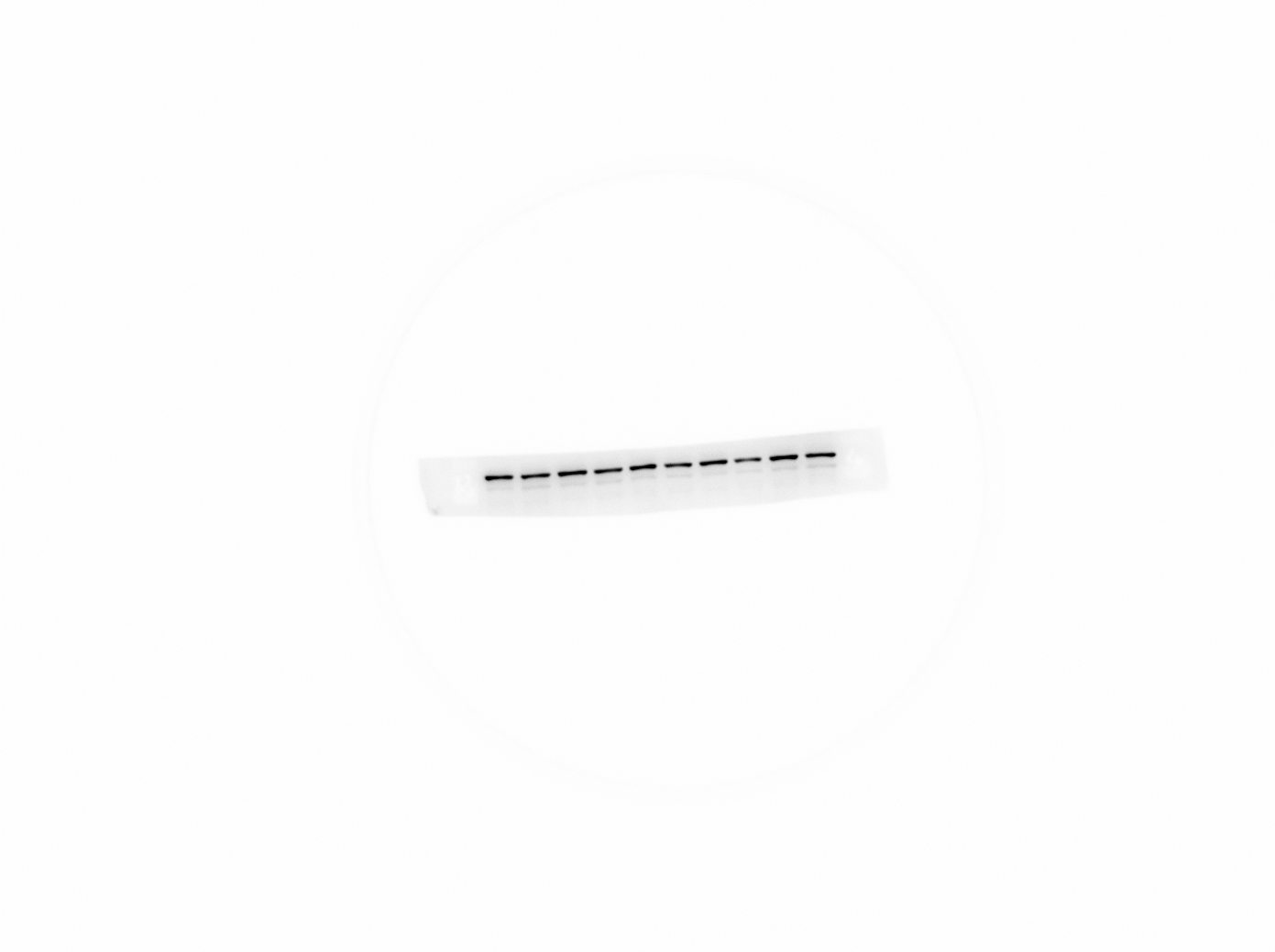

Supplement: Figure 5—source data 1. [file elife-75072-fig5-data1.zip › Figure 5-source data/figure5D/GRP78.tif]

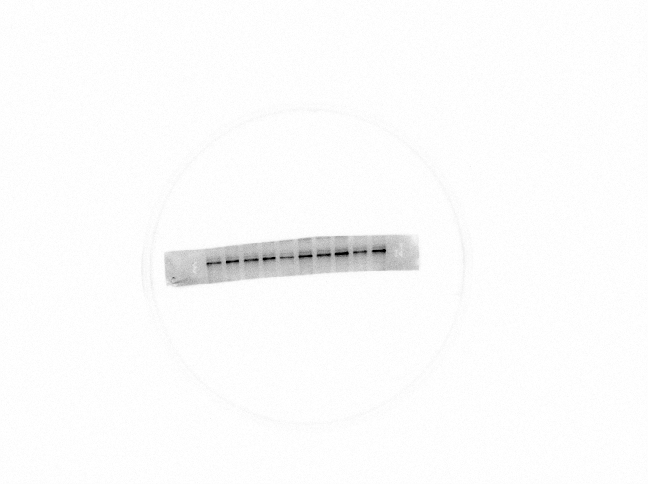

Supplement: Figure 5—source data 1. [file elife-75072-fig5-data1.zip › Figure 5-source data/figure5D/p-IRE1α.png]

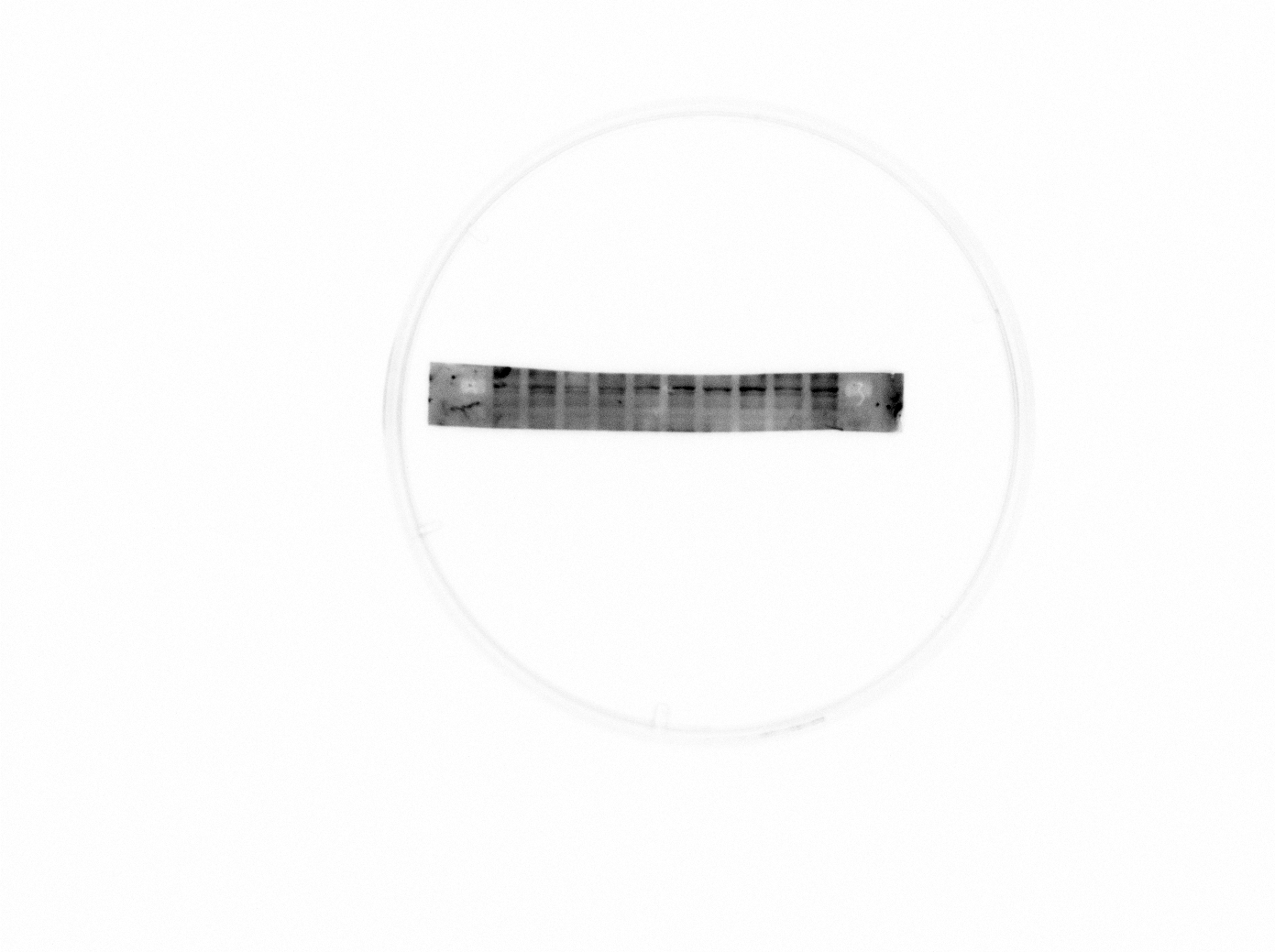

Supplement: Figure 5—source data 1. [file elife-75072-fig5-data1.zip › Figure 5-source data/figure5D/XBP1s.tif]

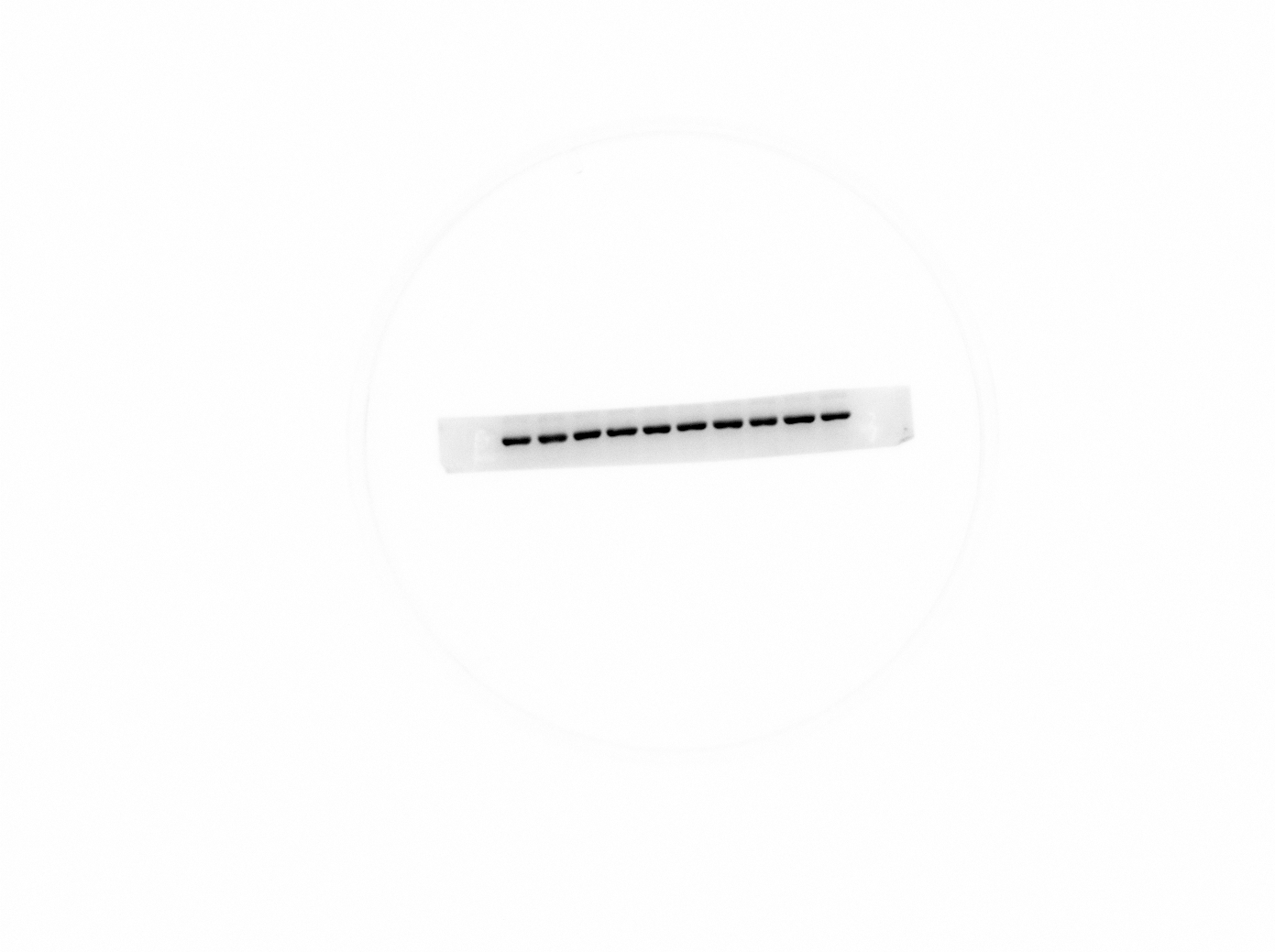

Supplement: Figure 5—source data 1. [file elife-75072-fig5-data1.zip › Figure 5-source data/figure5D/β-actin.tif]

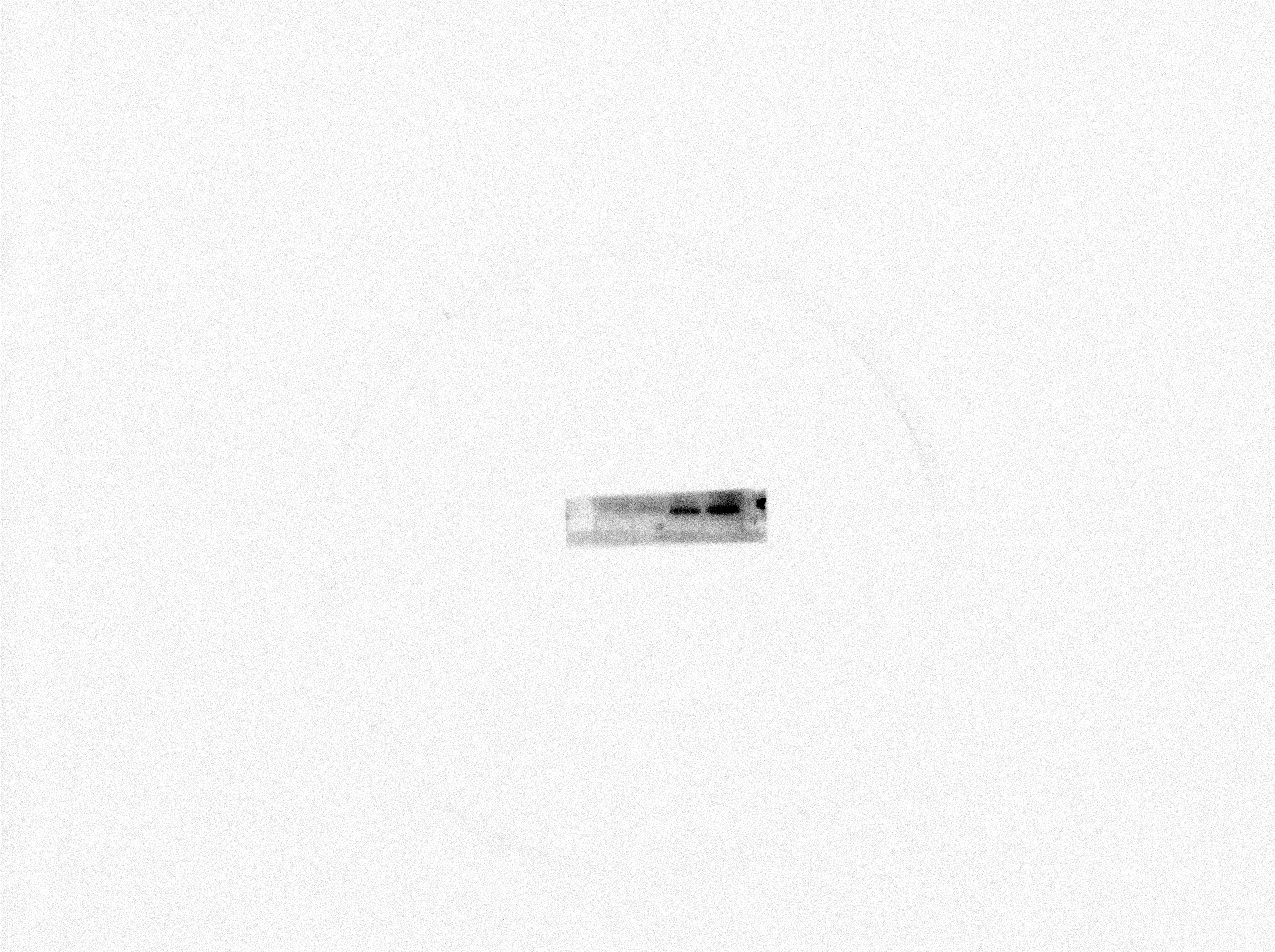

Supplement: Figure 6—source data 1. [file elife-75072-fig6-data1.zip › Figure 6-source data/figure6A/cox-2.tif]

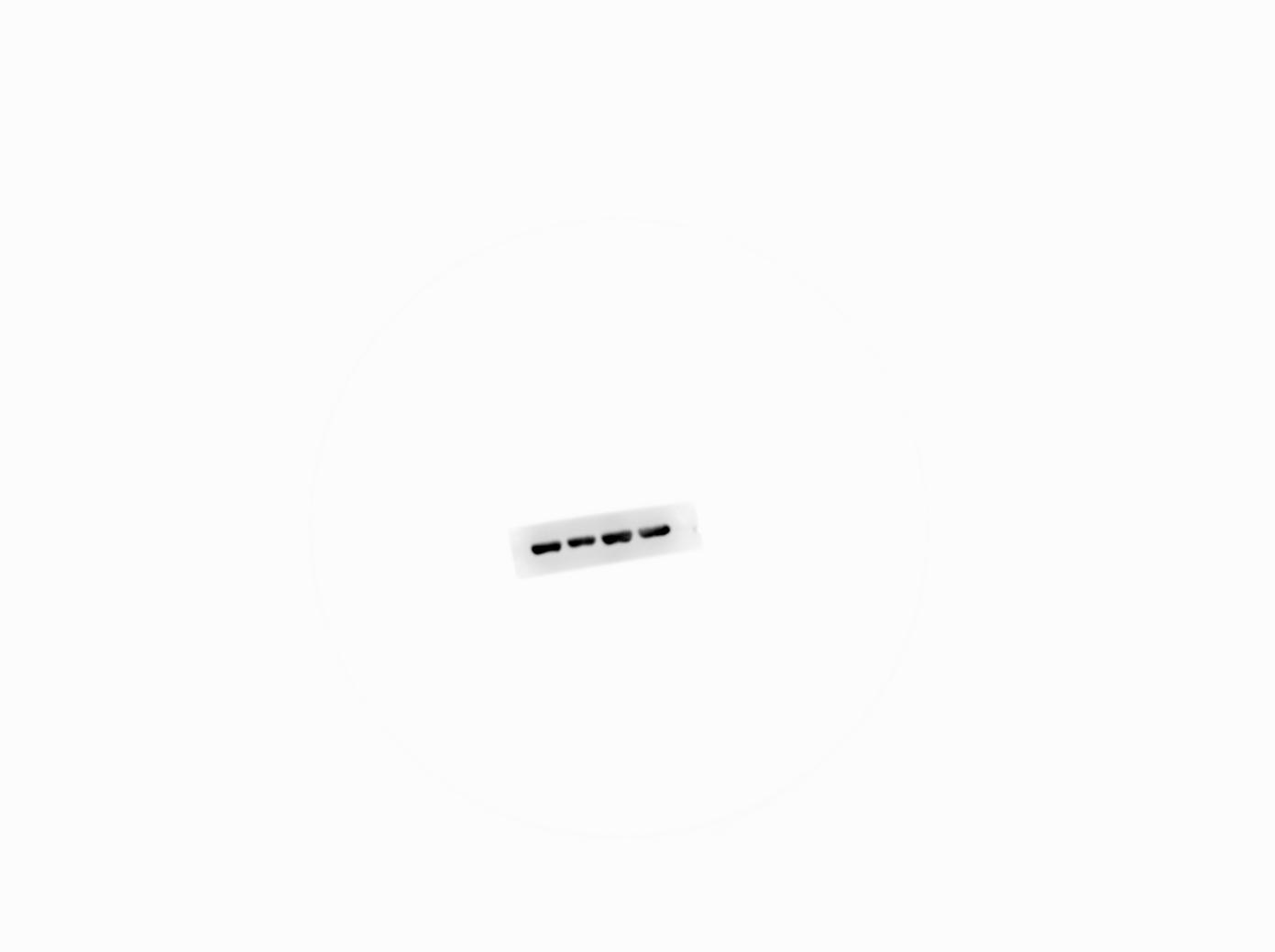

Supplement: Figure 6—source data 1. [file elife-75072-fig6-data1.zip › Figure 6-source data/figure6A/β-actin.tif]

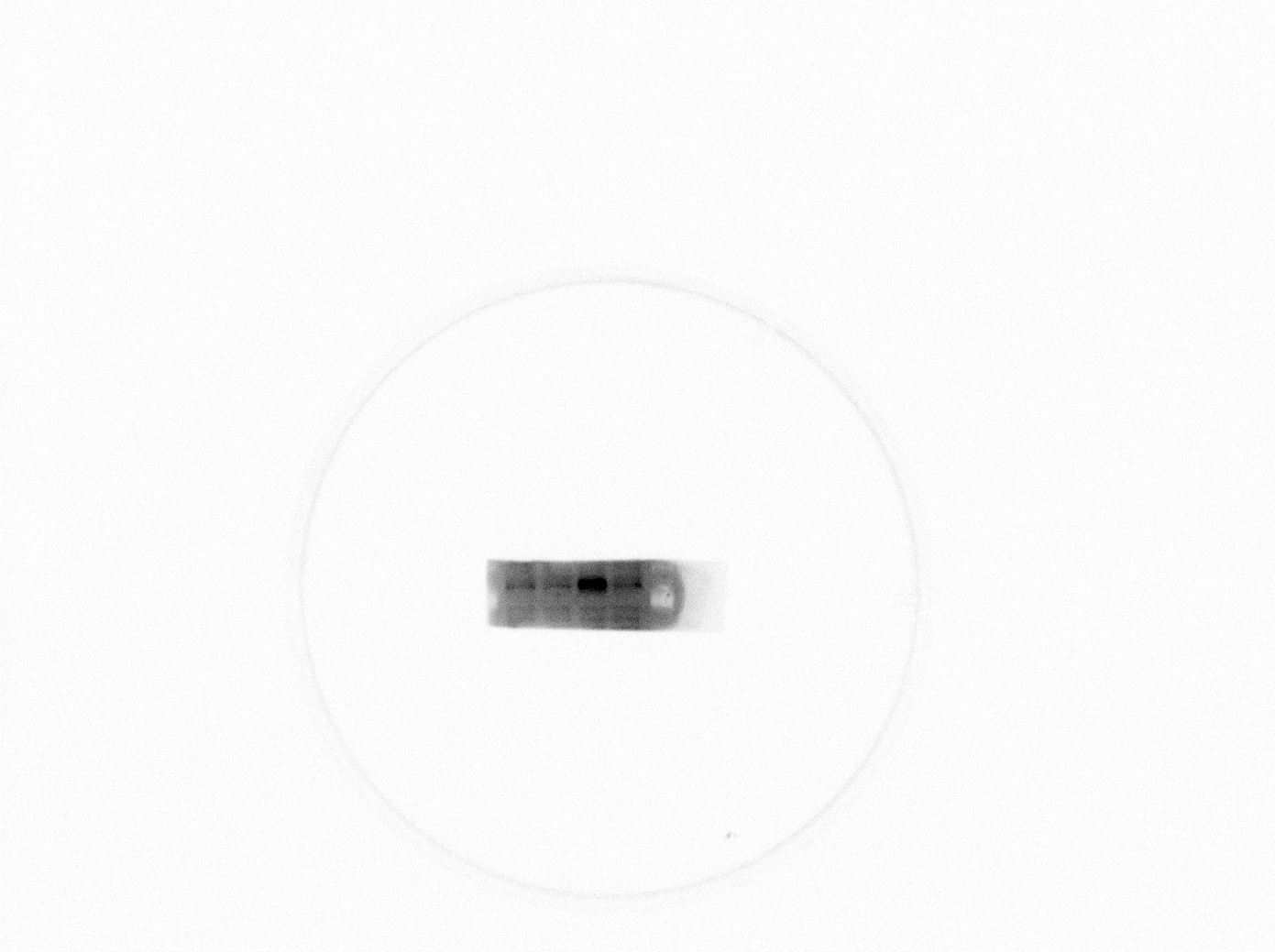

Supplement: Figure 6—source data 1. [file elife-75072-fig6-data1.zip › Figure 6-source data/figure6B/cox-2.tif]

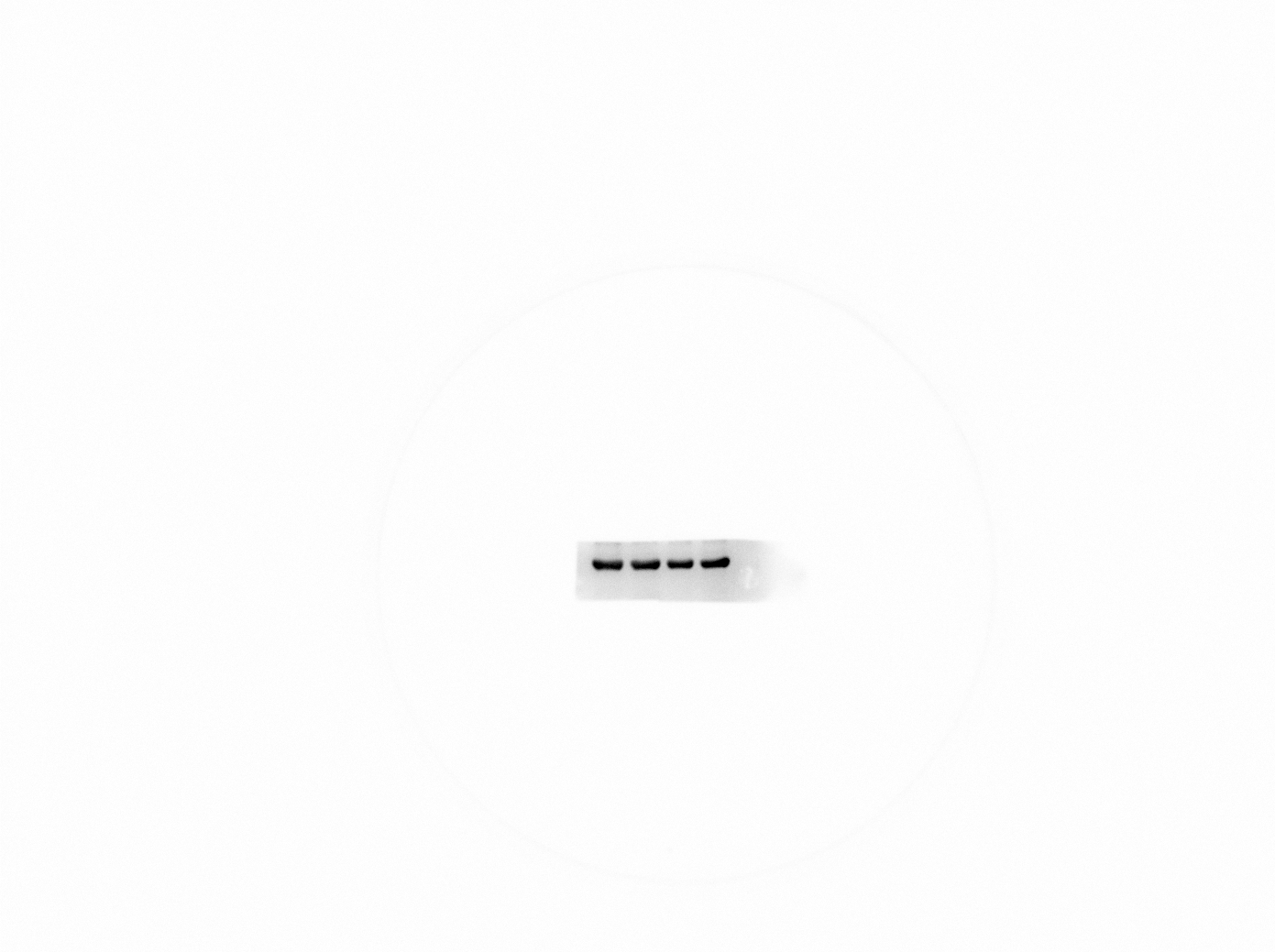

Supplement: Figure 6—source data 1. [file elife-75072-fig6-data1.zip › Figure 6-source data/figure6B/β-actin.tif]

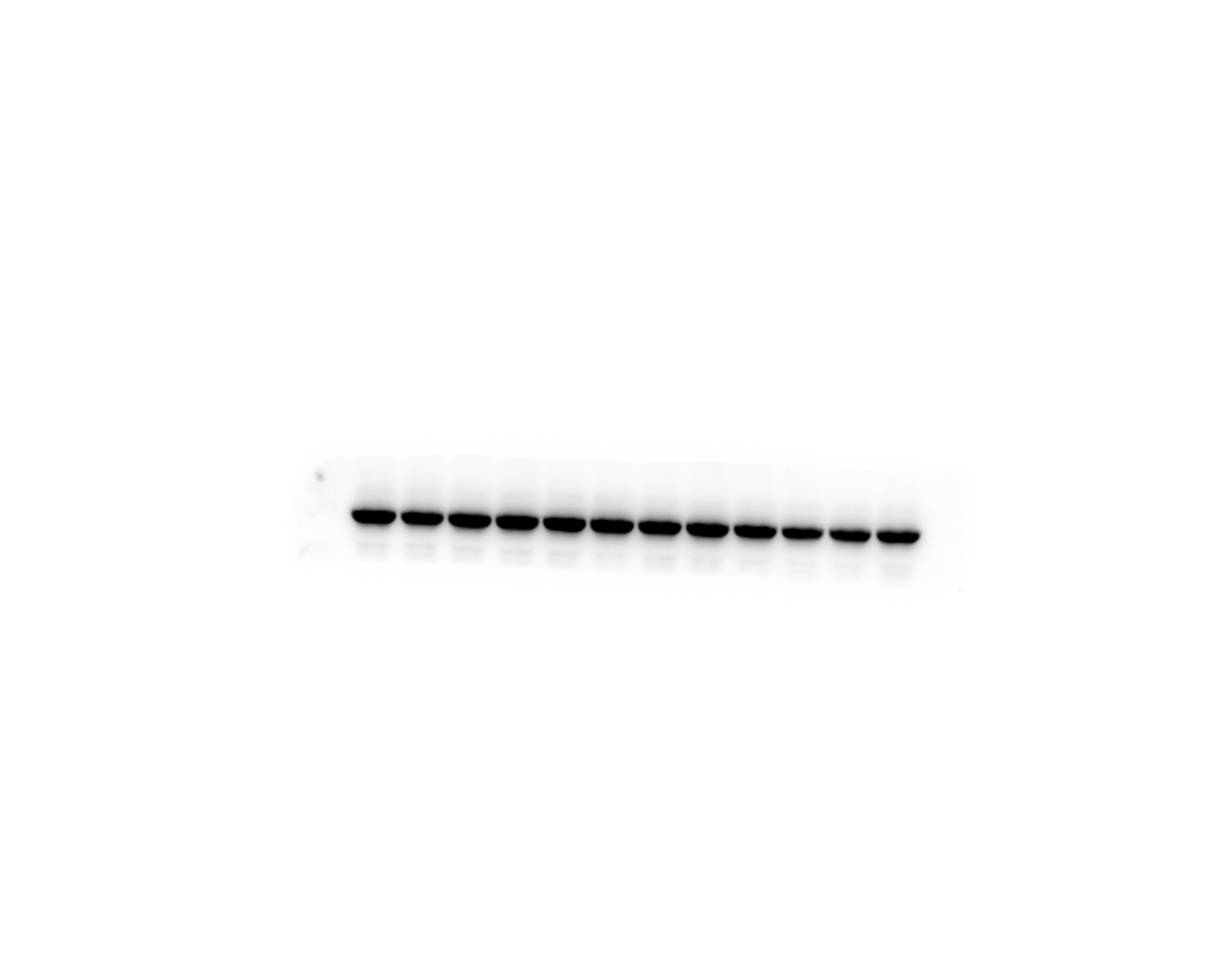

Supplement: Figure 7—source data 1. [file elife-75072-fig7-data1.zip › Figure 7-source data/figure7A/NF-κB.Tif]

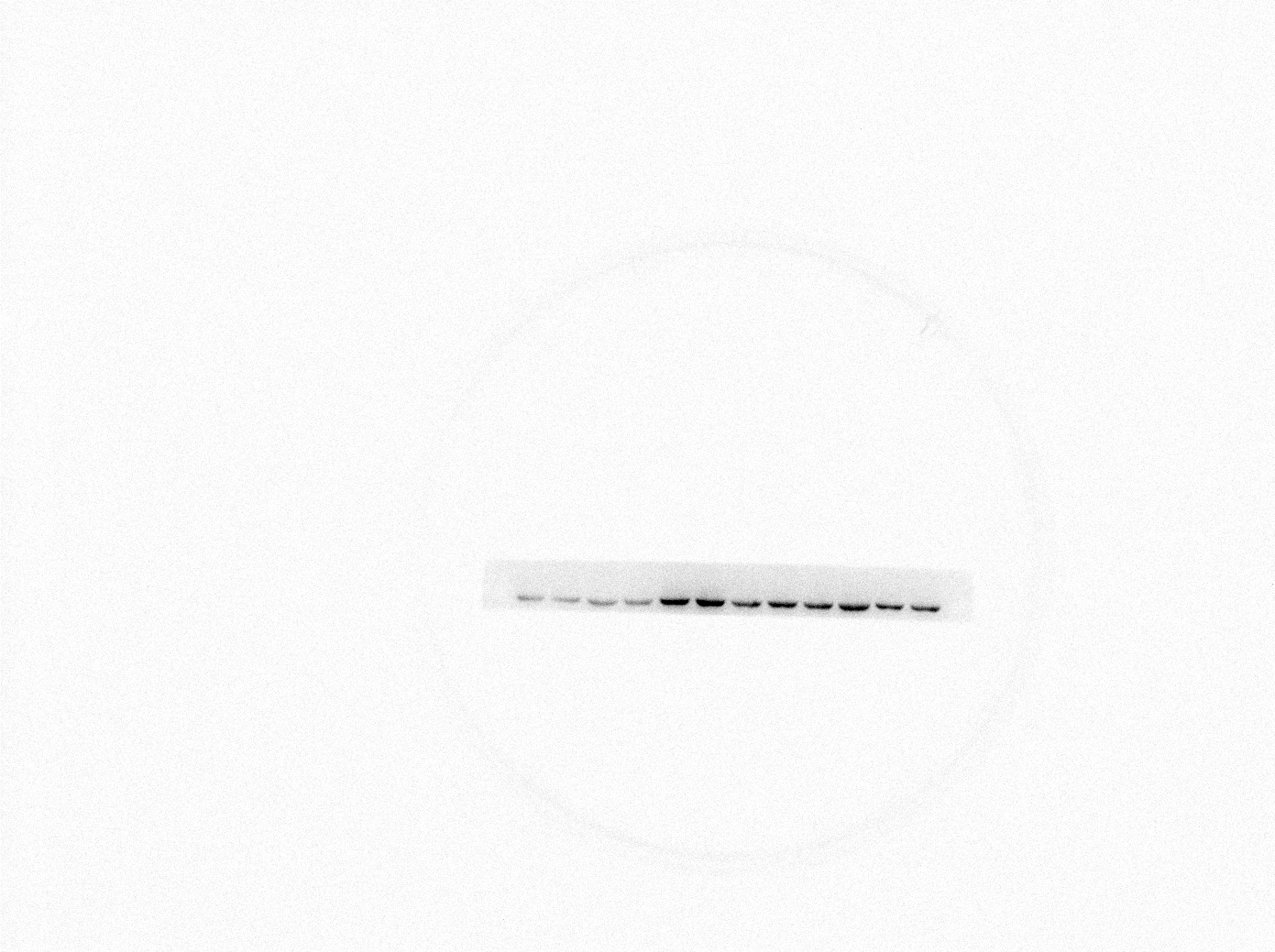

Supplement: Figure 7—source data 1. [file elife-75072-fig7-data1.zip › Figure 7-source data/figure7A/p-NF-κB.tif]

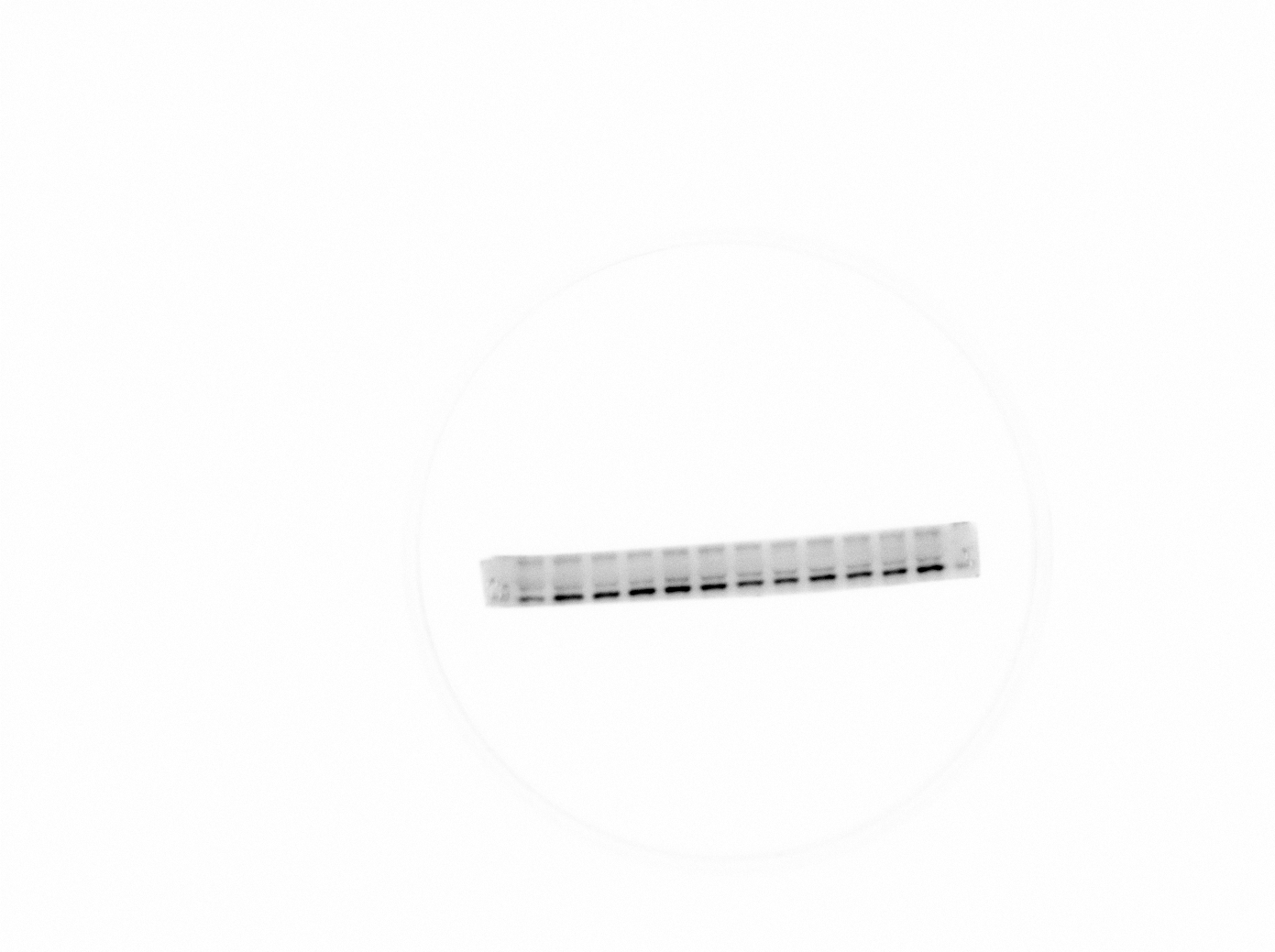

Supplement: Figure 7—source data 1. [file elife-75072-fig7-data1.zip › Figure 7-source data/figure7A/p-P38.tif]

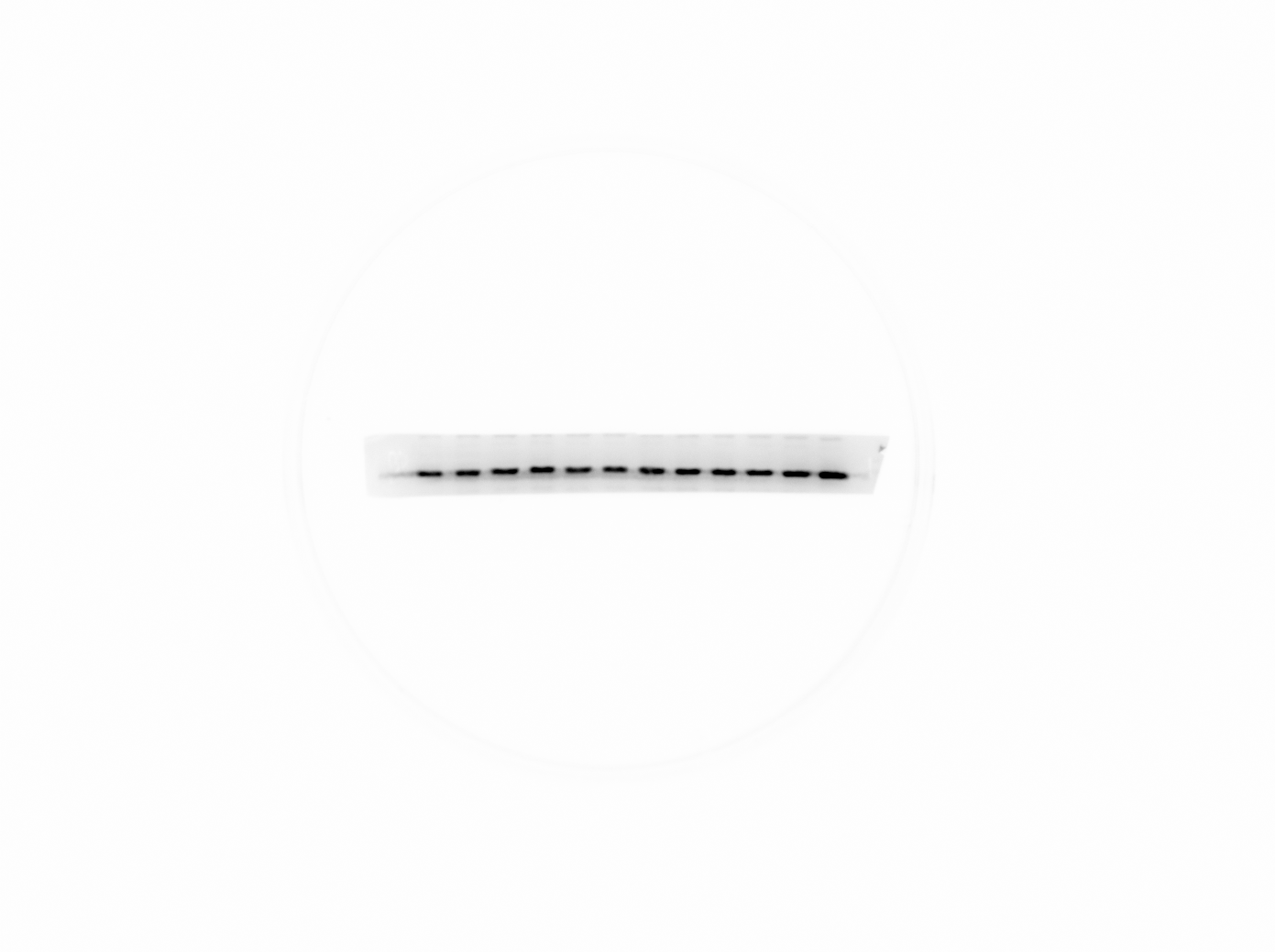

Supplement: Figure 7—source data 1. [file elife-75072-fig7-data1.zip › Figure 7-source data/figure7A/p38.tif]

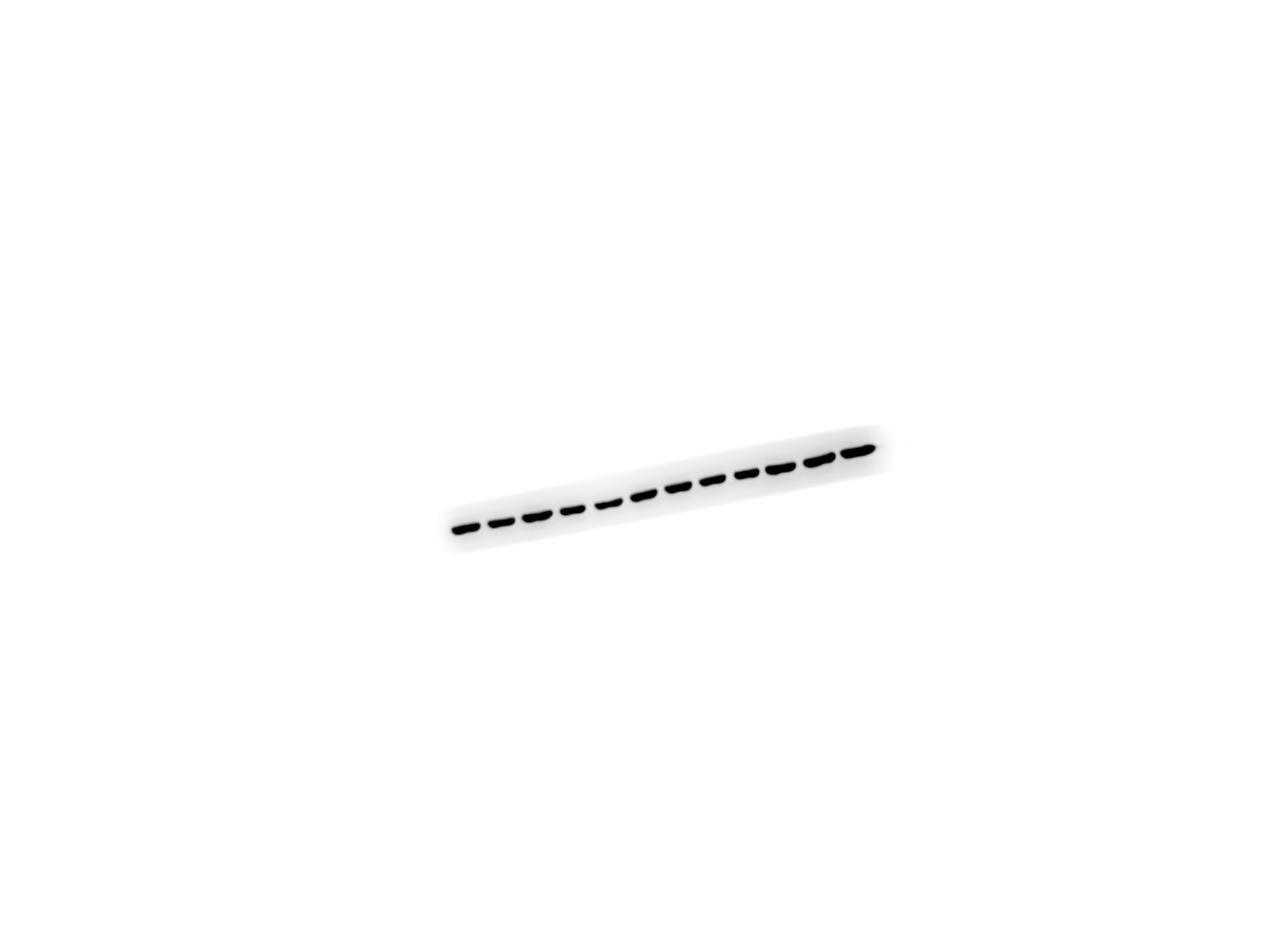

Supplement: Figure 7—source data 1. [file elife-75072-fig7-data1.zip › Figure 7-source data/figure7A/β-actin.tif]

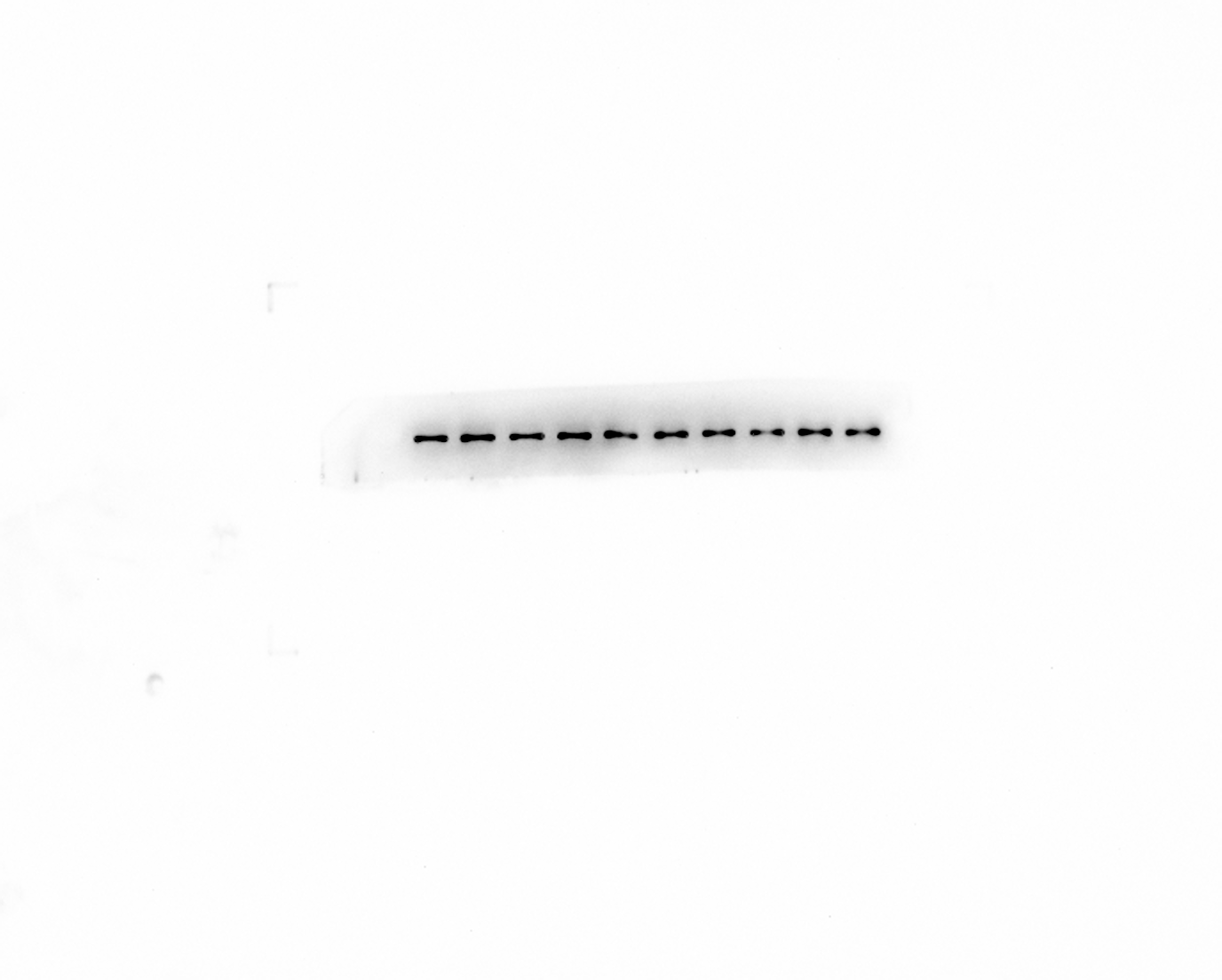

Supplement: Figure 7—source data 1. [file elife-75072-fig7-data1.zip › Figure 7-source data/figure7B/NF-κB/NF-κB.Tif]

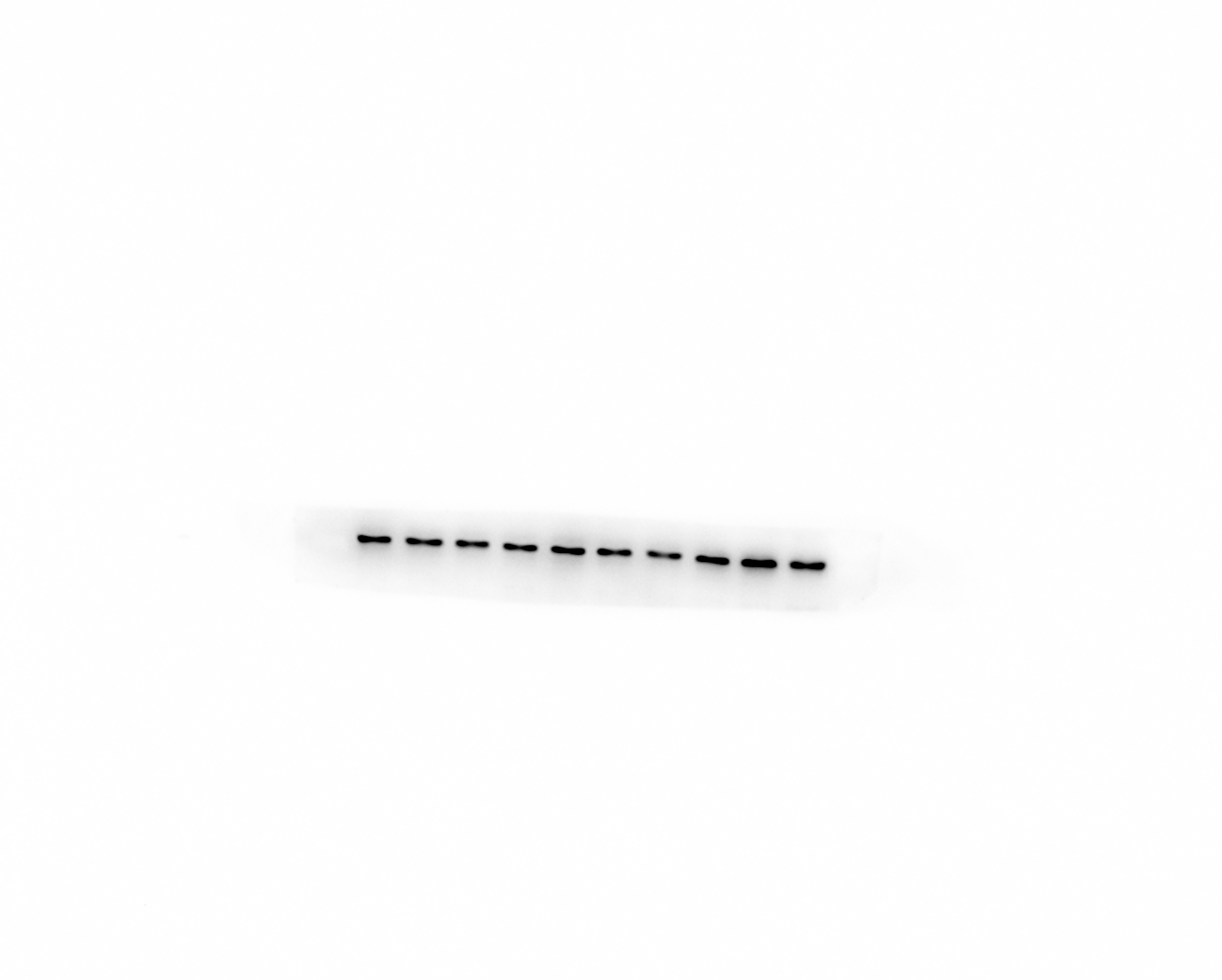

Supplement: Figure 7—source data 1. [file elife-75072-fig7-data1.zip › Figure 7-source data/figure7B/NF-κB/p-NF-κB.Tif]

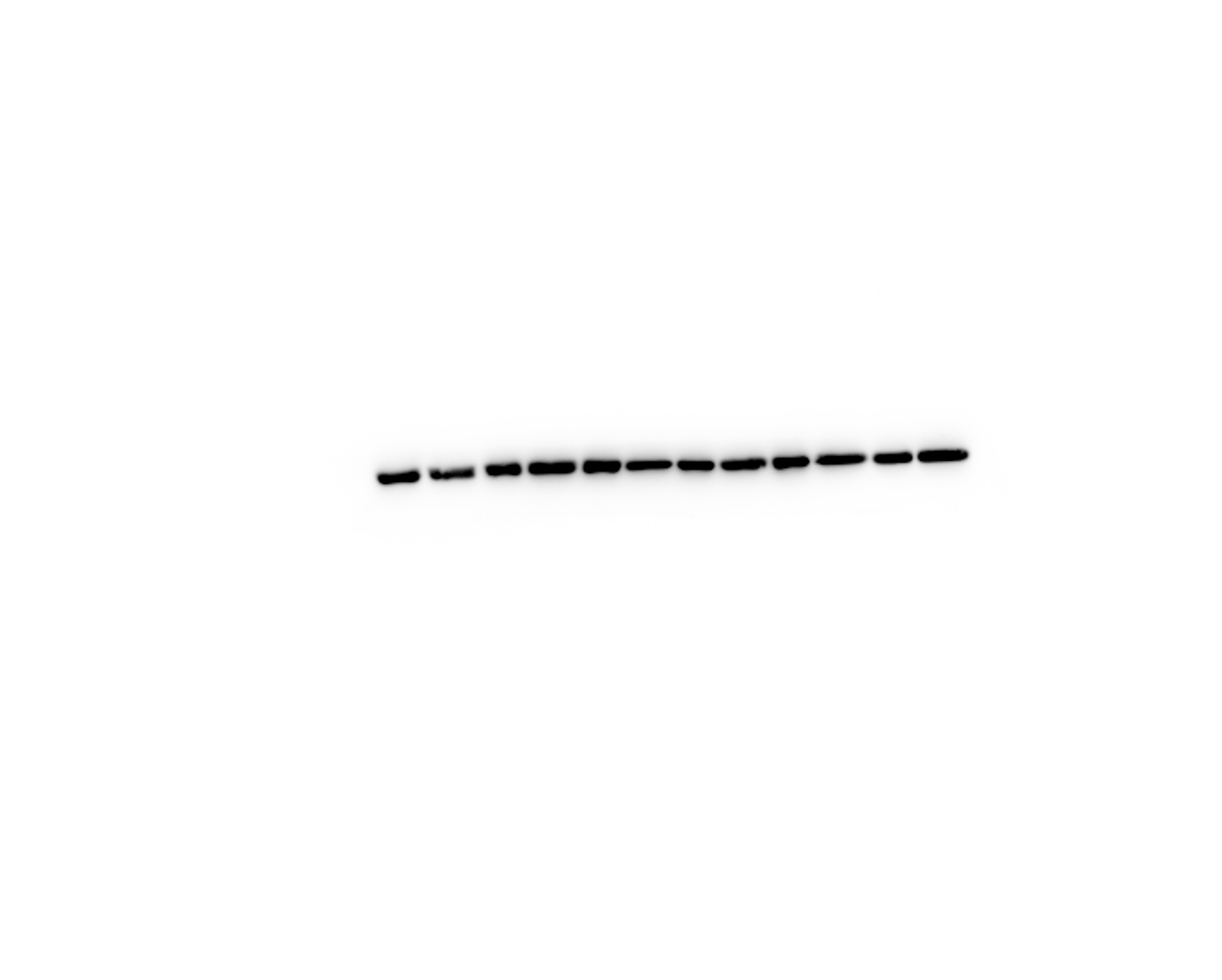

Supplement: Figure 7—source data 1. [file elife-75072-fig7-data1.zip › Figure 7-source data/figure7B/NF-κB/β-actin.Tif]

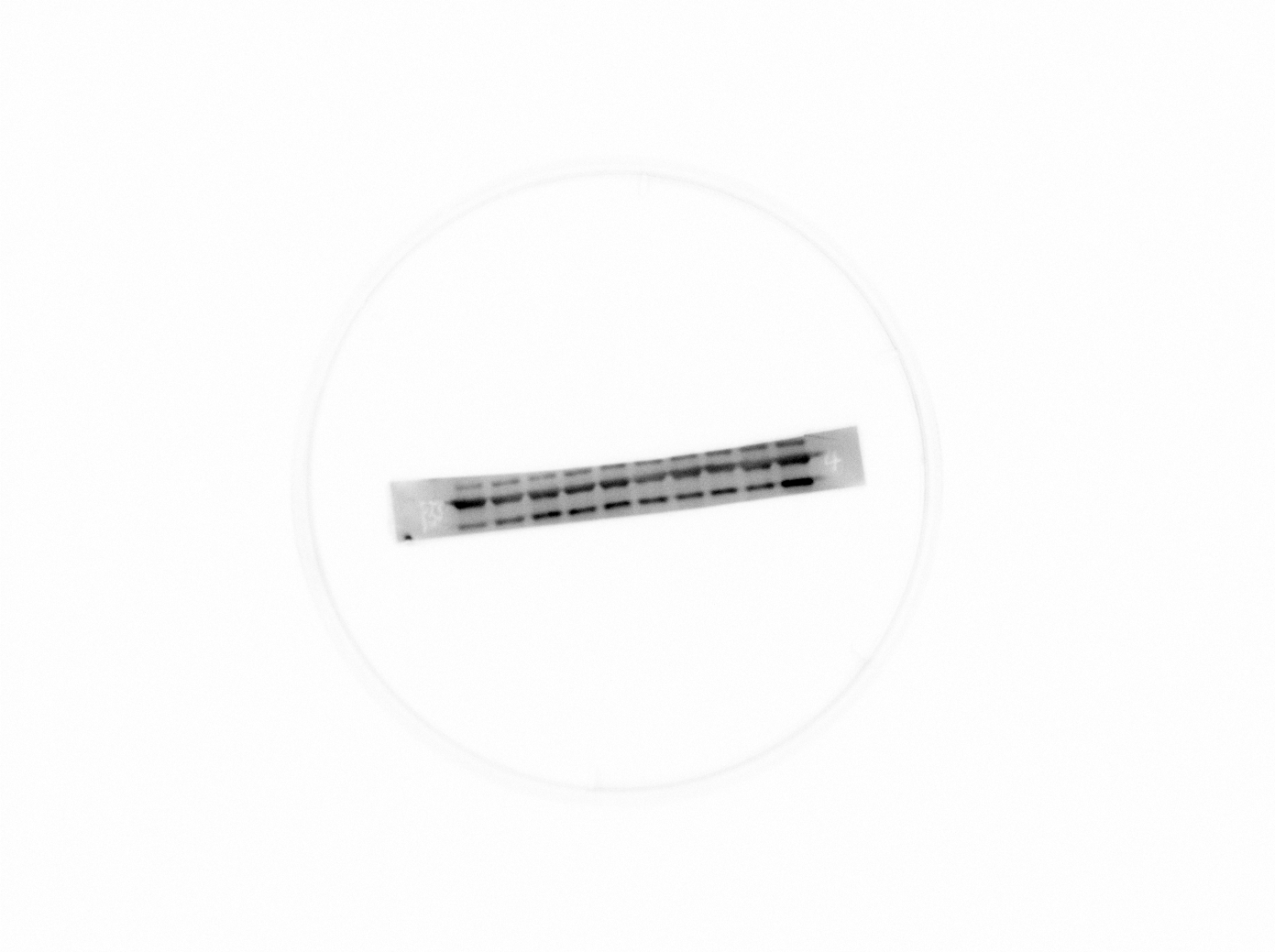

Supplement: Figure 7—source data 1. [file elife-75072-fig7-data1.zip › Figure 7-source data/figure7B/p38/p-P38.tif]

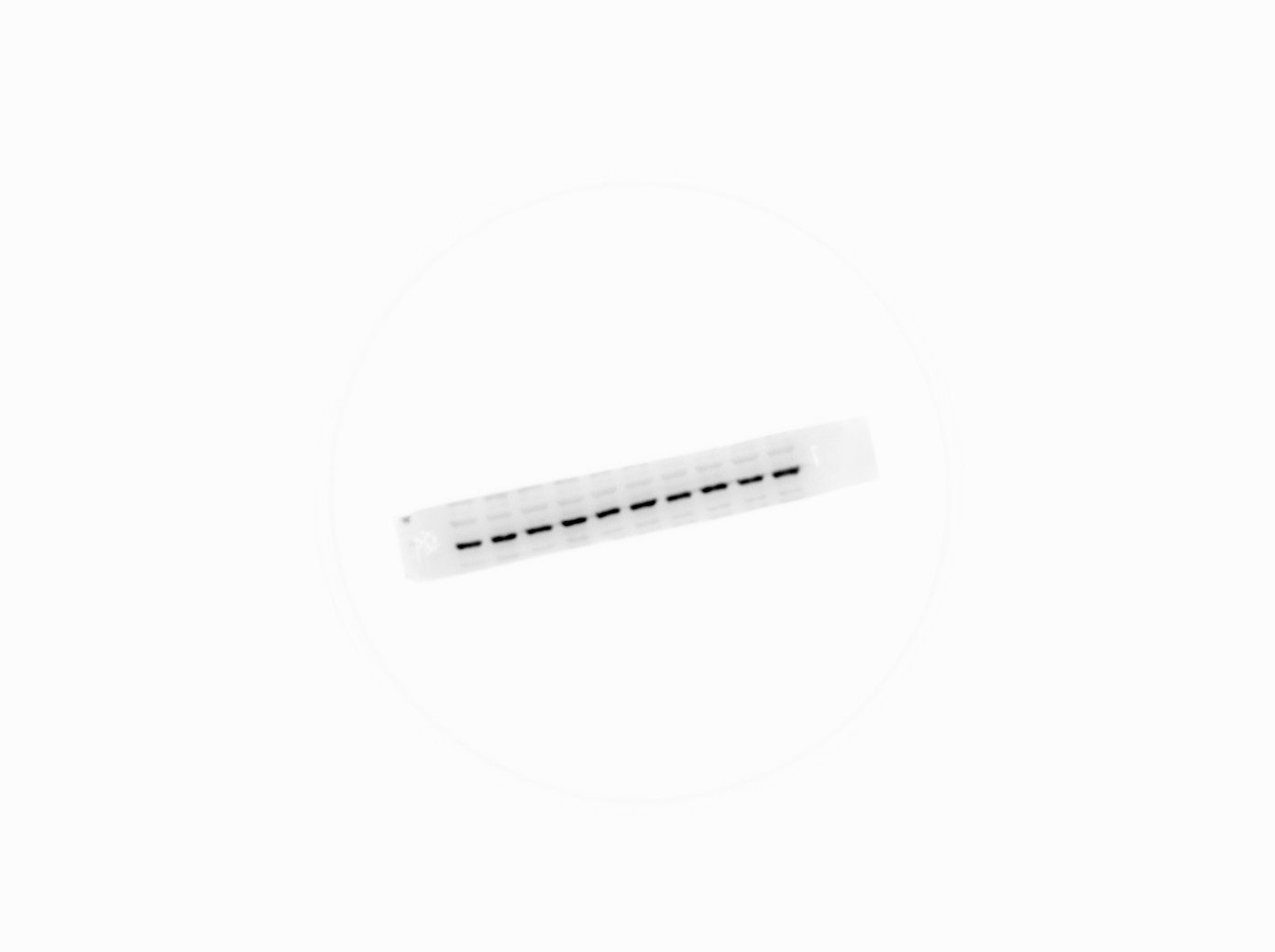

Supplement: Figure 7—source data 1. [file elife-75072-fig7-data1.zip › Figure 7-source data/figure7B/p38/p38.tif]

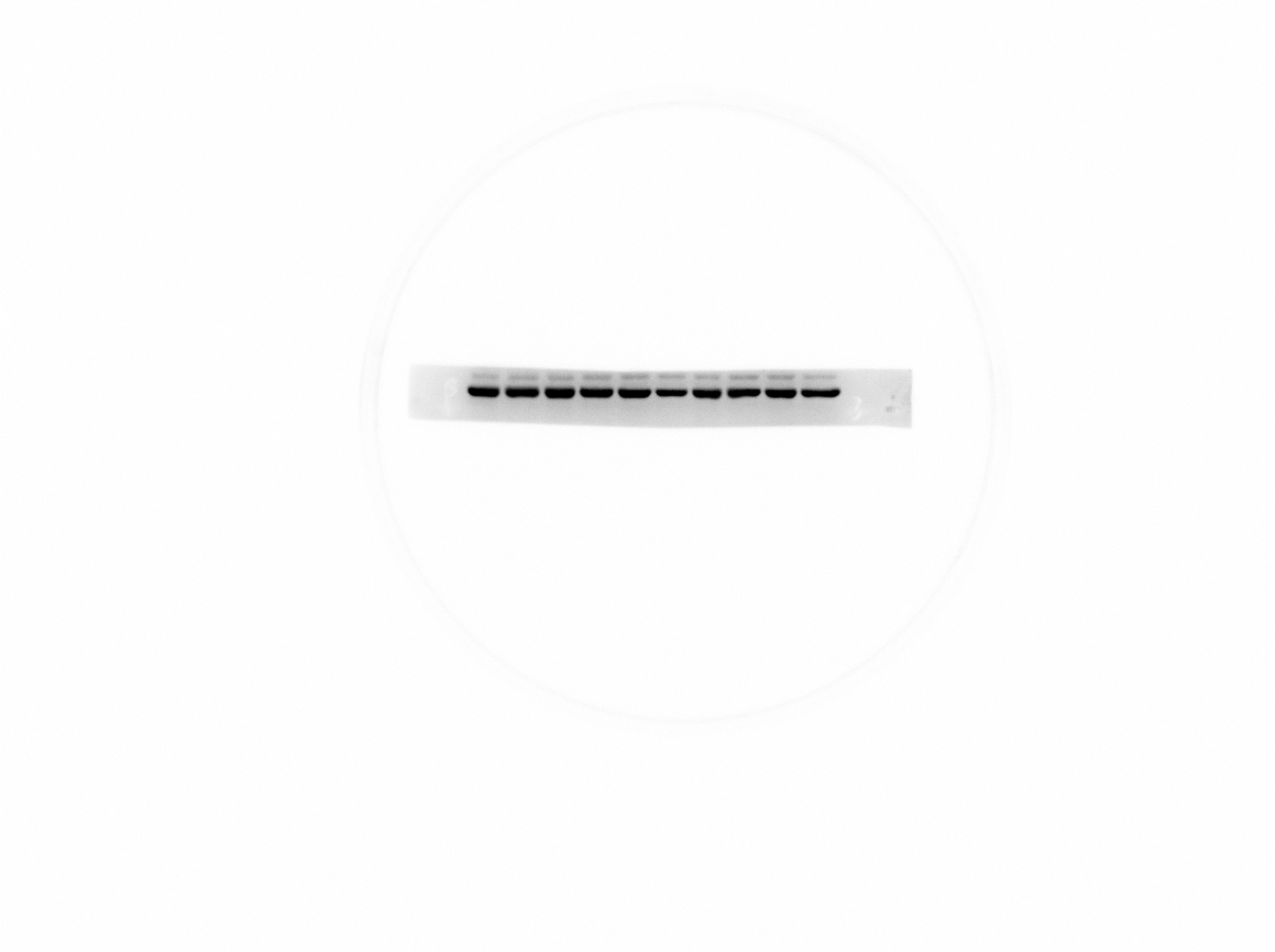

Supplement: Figure 7—source data 1. [file elife-75072-fig7-data1.zip › Figure 7-source data/figure7B/p38/β-actin.tif]

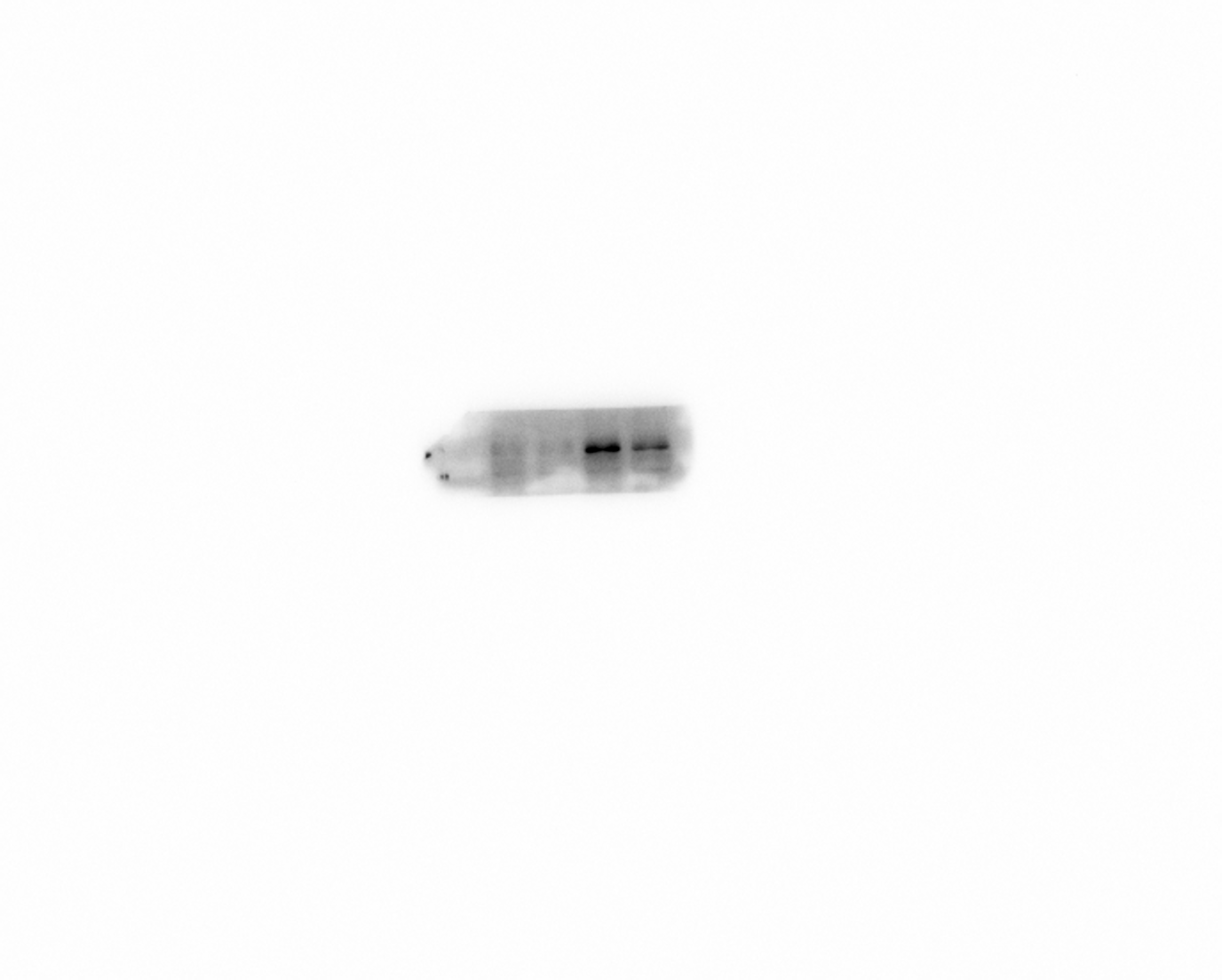

Supplement: Figure 7—source data 1. [file elife-75072-fig7-data1.zip › Figure 7-source data/figure7C/JSH-23/cox-2.Tif]

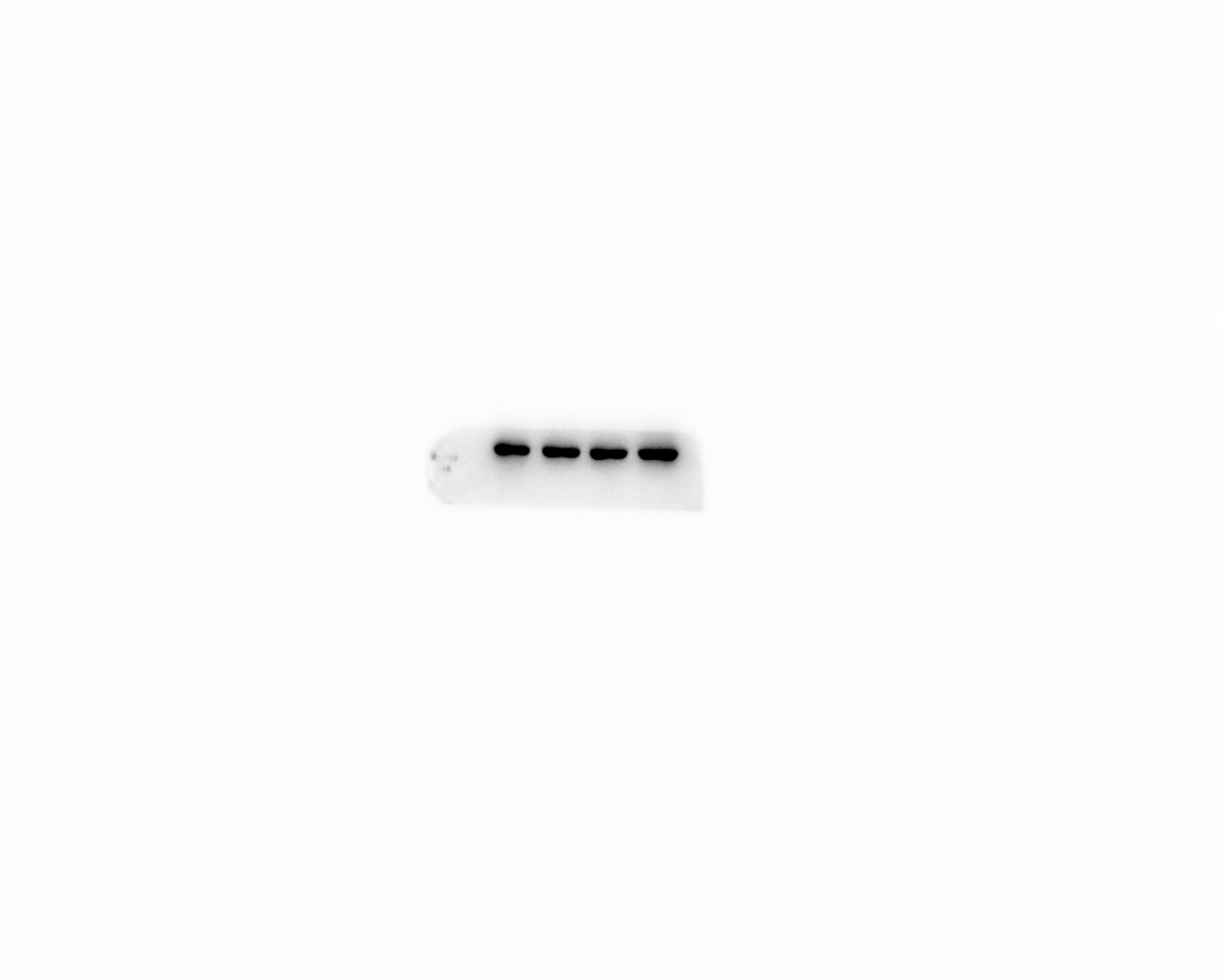

Supplement: Figure 7—source data 1. [file elife-75072-fig7-data1.zip › Figure 7-source data/figure7C/JSH-23/β-actin.Tif]

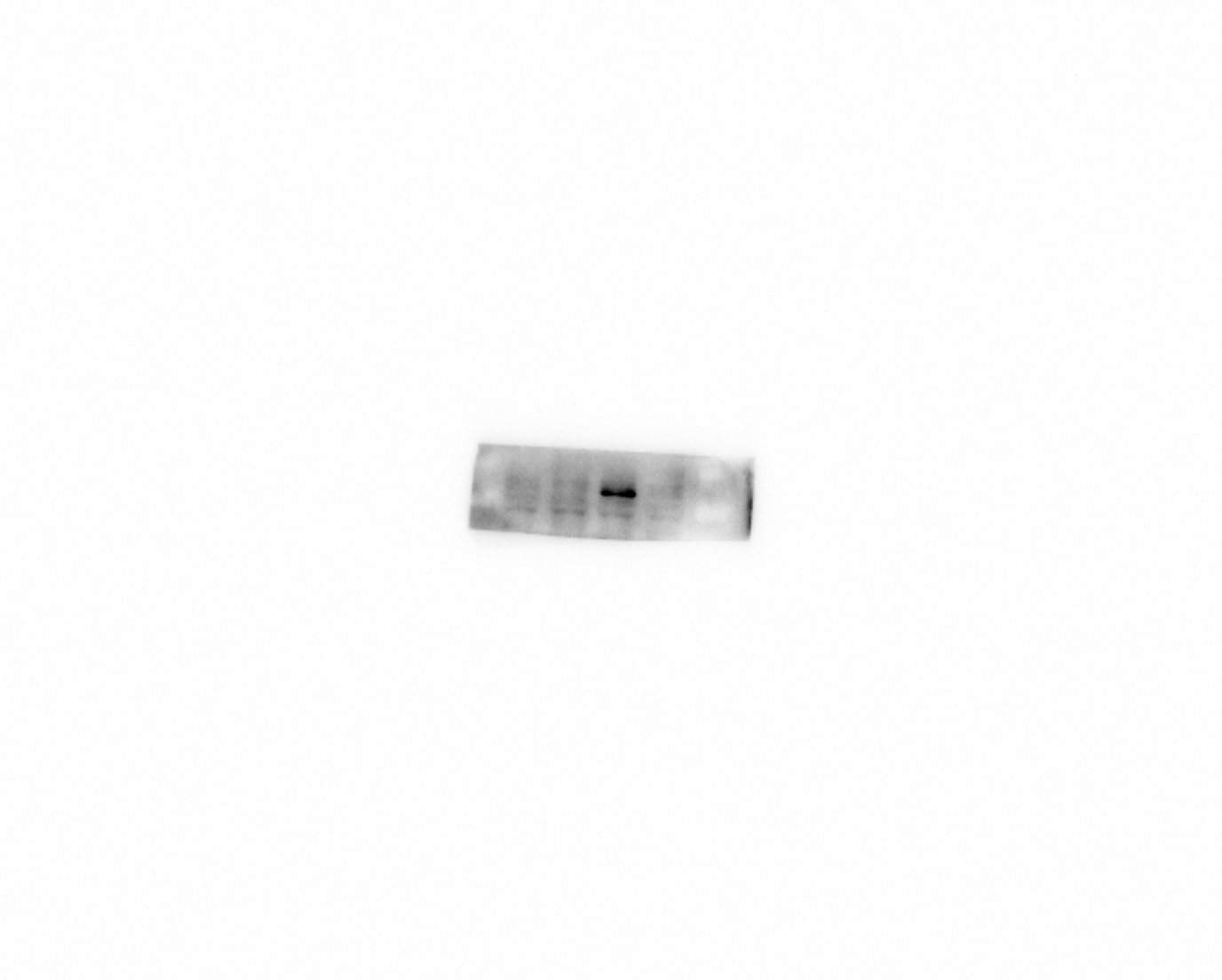

Supplement: Figure 7—source data 1. [file elife-75072-fig7-data1.zip › Figure 7-source data/figure7C/SB-202190/cox-2.Tif]

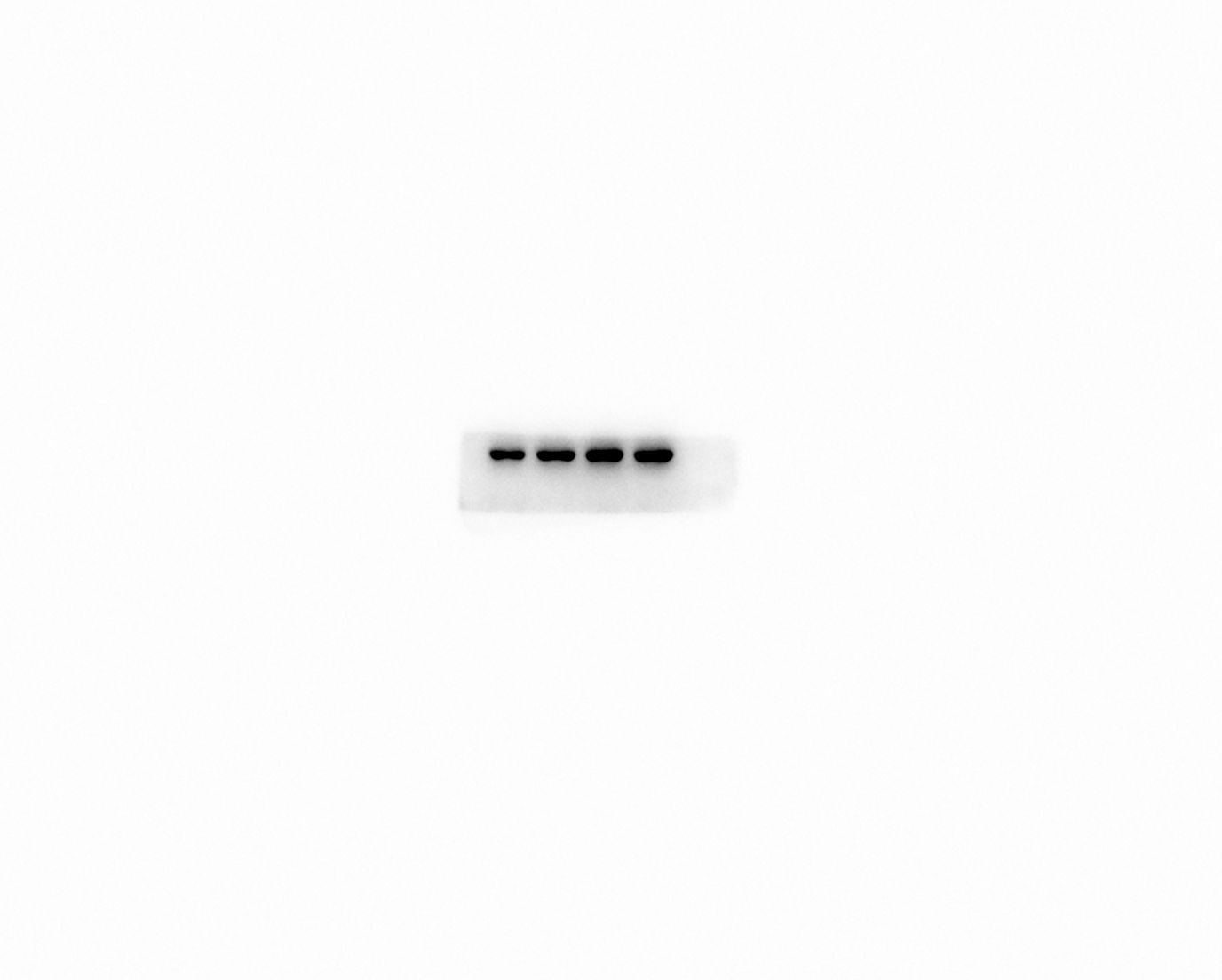

Supplement: Figure 7—source data 1. [file elife-75072-fig7-data1.zip › Figure 7-source data/figure7C/SB-202190/β-actin.Tif]
